# Supplementary material for: Prophylactic antibiotic use for infective endocarditis: a systematic review and meta-analysis
Source: BMJ Open. 2023 Aug 22;13(8):e077026. doi: 10.1136/bmjopen-2023-077026 (PMC10445353; doi:10.1136/bmjopen-2023-077026)
Supplement: Supplementary data [file bmjopen-2023-077026supp001.pdf]

Supplementary Materials

Supplementary Table 1: Patient characteristics with moderate or high risk of infective endocarditis according to the AHA and ESC guidelines

Moderate risk	High risk
Uncorrected congenital cardiac conditions e.g. bicuspid aortic valve, coarctation of the aorta, patent ductus arteriosus, atrial or ventricular septal defects	Prosthetic heart valves or repaired valves using prosthetic material
Acquired valvular diseases e.g. Rheumatic heart disease	Previous history of endocarditis
Hypertrophic cardiomyopathy	Complex cyanotic congenital heart disease
	Systemic pulmonary shunts or conduits constructed via surgery

Supplementary Table 2: Search strategy by databases

**Database**	**Search term**
Medline	("dent*"[All Fields] OR "dontic*"[All Fields]) AND "endocarditis"[All Fields]
Embase	((“dent*” or “*dontic*”) and “endocarditis”).mp.
Scopus	TITLE-ABS-KEY (("dent*" OR "dontic") AND "endocarditis")
Clinicaltrials.gov	Endocarditis

Supplementary Table 3: Inclusion criteria

	**Inclusion criteria**
Population	• Adults age >18 years
Exposure/Intervention	• Any dental procedures 3 months prior to diagnosis of infective endocarditis such as tooth extraction, fillings, scaling, endodontal and periodontal treatment, implant insertion, root canal treatment
Comparison (if available)	• No dental procedure within 3 months of infective endocarditis
Outcome	• Diagnosis of infective endocarditis within 3 months of dental procedure

Supplementary Table 4: Quality assessment of included studies using the Newcastle-Ottawa Scale

**Author, Year, Country**	**Selection**				**Comparability**	**Outcome**			**Total quality score**
	Cohort studies: Representativeness of the exposed cohort	Selection of non-exposed cohort	Ascertainment of exposure	Demonstration that the current outcome of interest was not present at the start of study	Comparability of the cohorts on the basis of design or analysis	Ascertainment of outcome	Was follow up long enough for outcomes to occur	Adequacy of follow up of cohorts	
	Case control studies: Case definition adequate	Representativeness of cases	Selection of controls	Definition of controls	Comparability of cases and controls on the basis of design or analysis	Ascertainment of exposure	Same method of ascertainment for cases and controls	Non-response rate	
Aziz et al., 2010, US	★	★		★		★			4
Ballesta et al., 2022, Spain	★	★	★	★	★★	★	★		8
Carmona et al., 2002, Spain	★	★	★	★		★	★		6
Carmona et al., 2003, Spain	★	★	★	★	★	★	★		7
Chang et al., 2017, Taiwan	★	★	★	★	★★	★	★	★	8
Chen et al., 2015, Taiwan	★	★	★	★	★	★	★	★	8
Chen et al., 2018, Taiwan	★	★	★	★	★★	★	★	★	9

Chirillo et al., 2016, Italy	★	★	★	★		★	★	★	7
Chu et al., 2004, New Zealand	★	★		★		★	★		5
Cukingnan et al., 1983, US	★	★	★	★		★	★	★	7
Delahaye et al., 2016, France	★	★	★	★		★	★	★	7
Dominguez et al., 2016, Spain	★	★	★	★	★★	★	★		8
Durack et al., 1983, US				★			★		2
Duval et al., 2006, France	★	★	★	★	★	★	★	★	8
Duval et al., 2017, France	★	★	★	★	★★		★	★	8
Findler et al., 2014, Israel	★	★	★	★		★	★		7
Grover et al., 1991, India	★	★	★	★		★	★	★	7
Hricak et al., 2013, Slovakia	★	★	★	★		★	★	★	7
Imperiale et al., 1990, US	★	★	★	★	★★	★	★		8

Kim et al., 2019, Korea	★	★	★	★		★	★	★	7
Krcmery et al., 2018, Slovakia	★	★	★	★		★	★	★	7
Lacassin et al., 1995, France	★	★		★	★★		★		6
Littner et al., 1986, Israel	★			★		★	★		4
Loupa et al., 2004, Greece	★	★	★	★		★	★		6
Luk et al., 2014, Canada	★	★	★	★		★	★		6
Martin et al., 2007, UK	★		★	★		★	★		5
Mudhumitha et al., 2018, India	★	★	★	★		★	★	★	7
Porat Ben-Amy et al., 2009, Israel	★	★	★	★	★★	★	★		8
Russo et al., 2000, Italy	★	★		★			★		4
Santinga et al., 1976, US			★	★			★		3
Sett et al., 1993, Canada	★	★	★	★	★★	★	★	★	9

Siegman-Igra et al., 2010, Israel	★	★	★	★		★	★		6
Smith et al., 1976, UK	★			★			★		3
Strom et al., 1998, US	★	★	★	★	★★	★	★		8
Takeda et al., 2005, Japan	★	★	★	★		★	★		6
Tubiana et al., 2017, France	★	★	★	★	★★	★	★	★	9
Tzukert et al., 1986, Israel				★			★		2
Weinberger et al., 1990, Israel	★	★	★	★		★	★		6

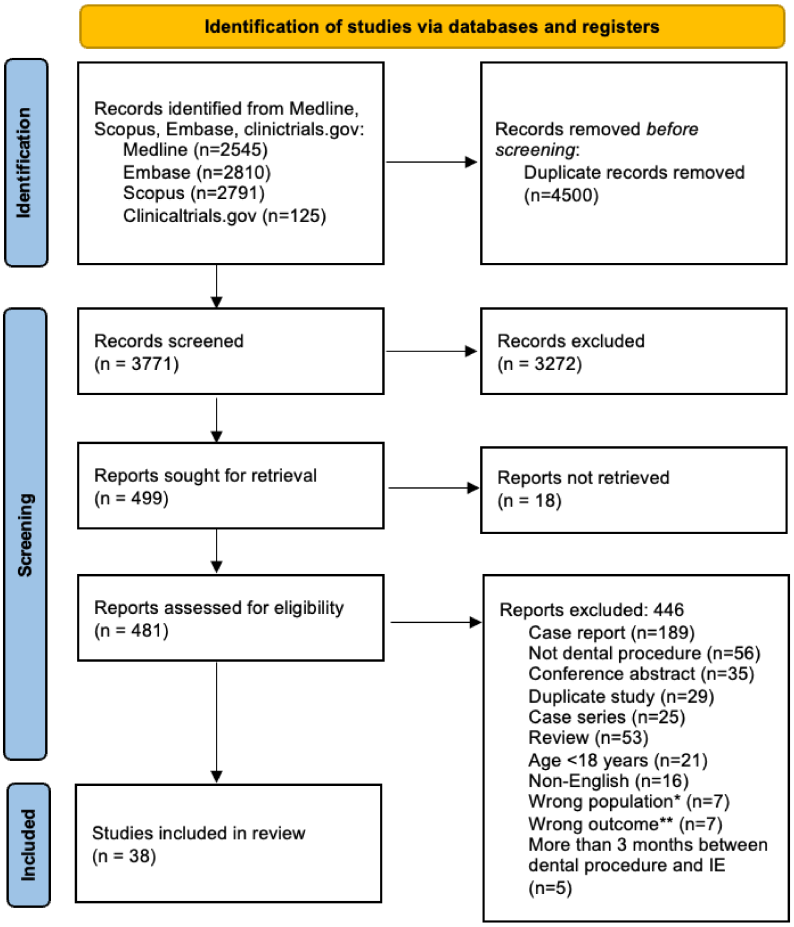

*"Wrong population" refers to studies that did not include patients with IE and dental procedures

**"wrong outcomes" refers to studies that did not report findings relevant to the conclusions of the present systematic review

Supplementary Figure 1. PRISMA flow diagram of the study selection process

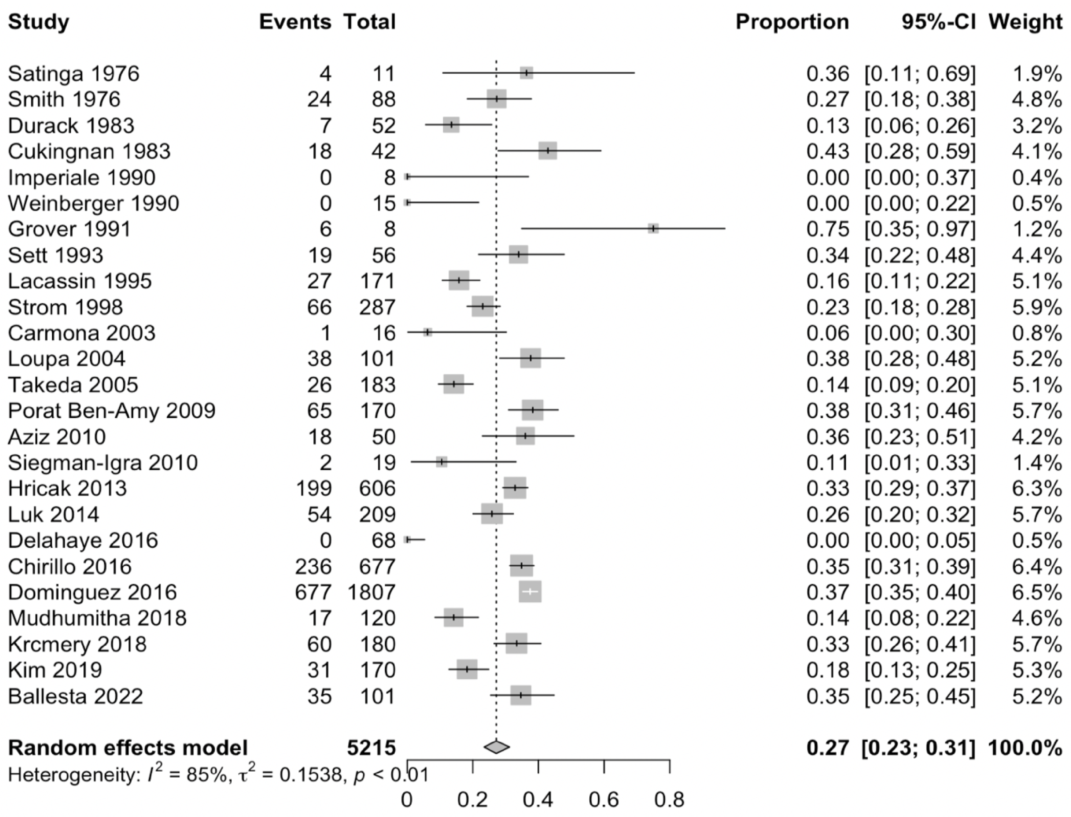

Supplementary Figure 2A. Proportion of *Staphylococcus* bacteraemia

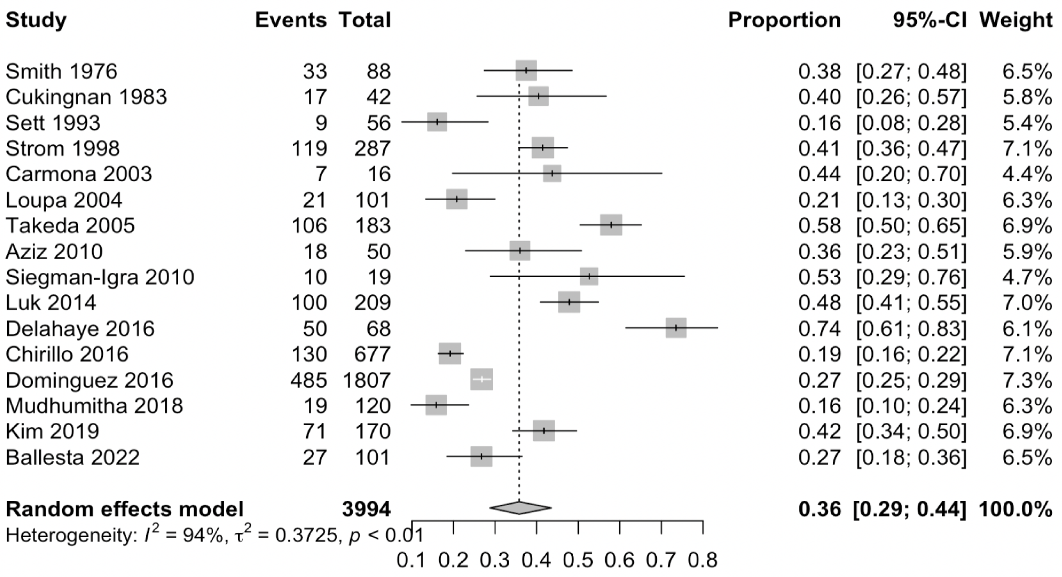

Supplementary Figure 2B. Proportion of *Streptococcus* bacteraemia

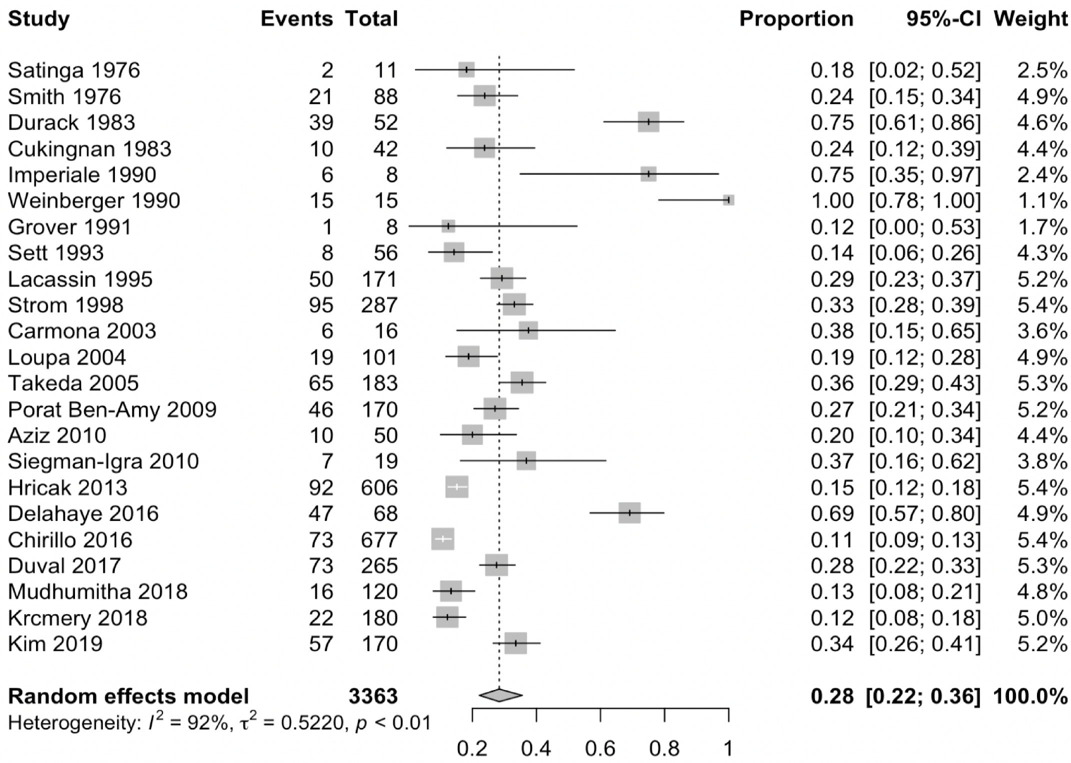

Supplementary Figure 2C. Proportion of *Streptococcus viridans* bacteraemia

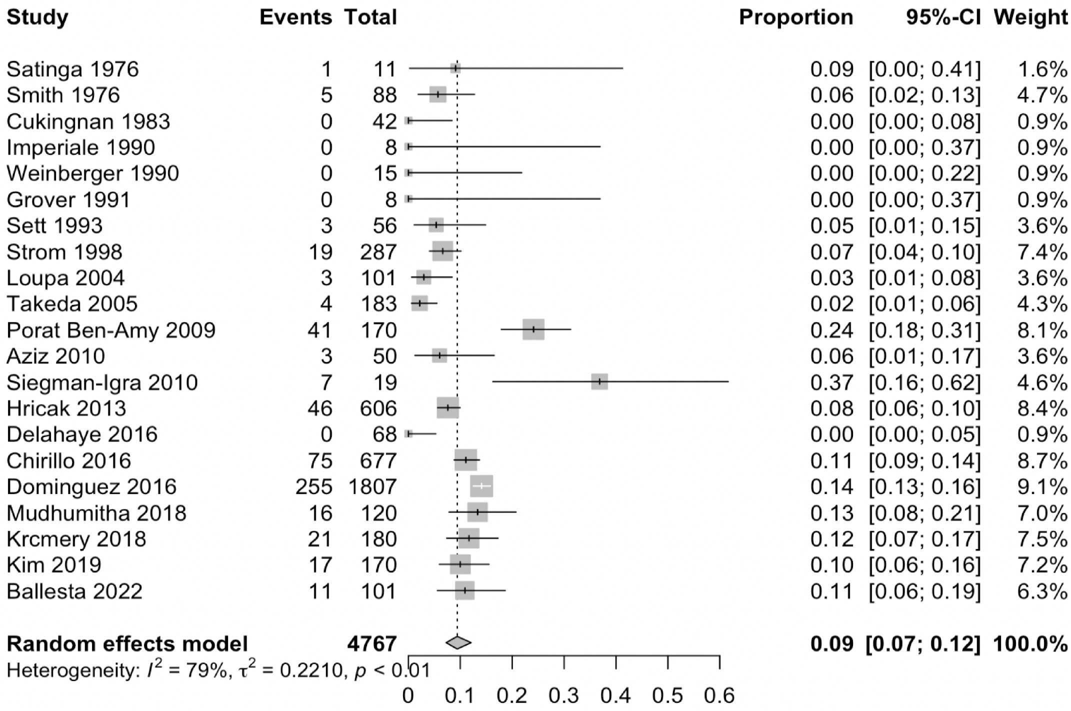

Supplementary Figure 2D. Proportion of *Enterococcus* bacteraemia

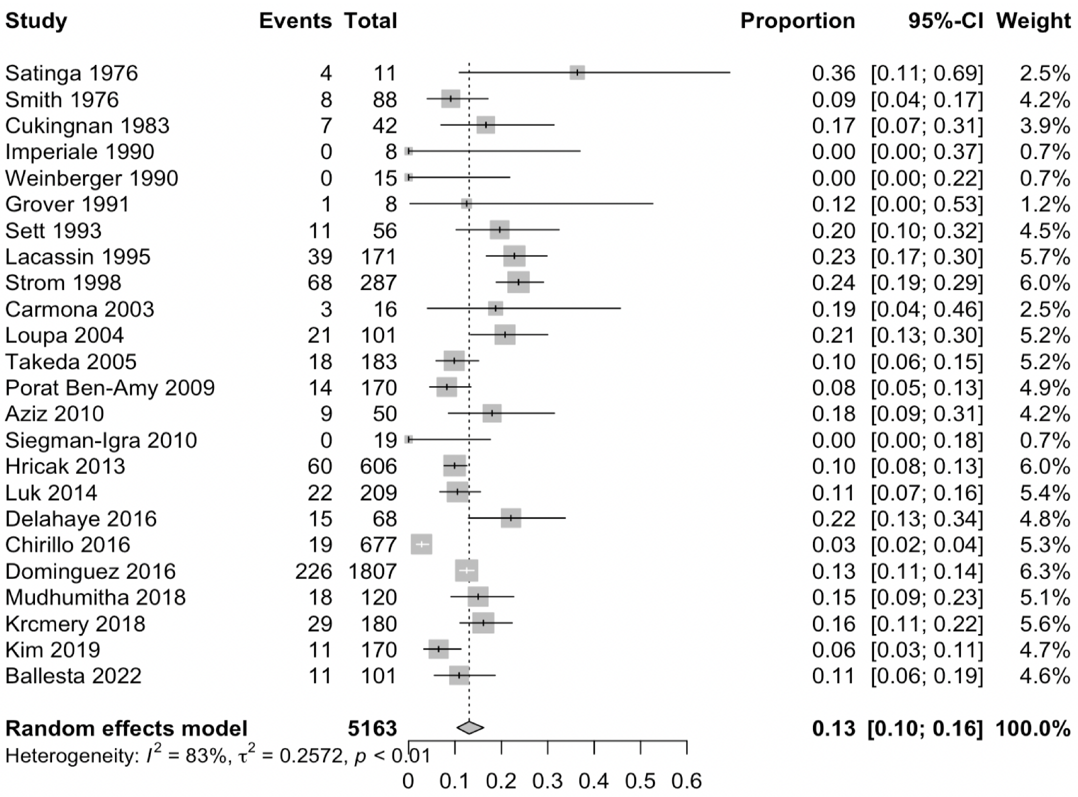

Supplementary Figure 2E. Proportion of HACEK and others bacteraemia

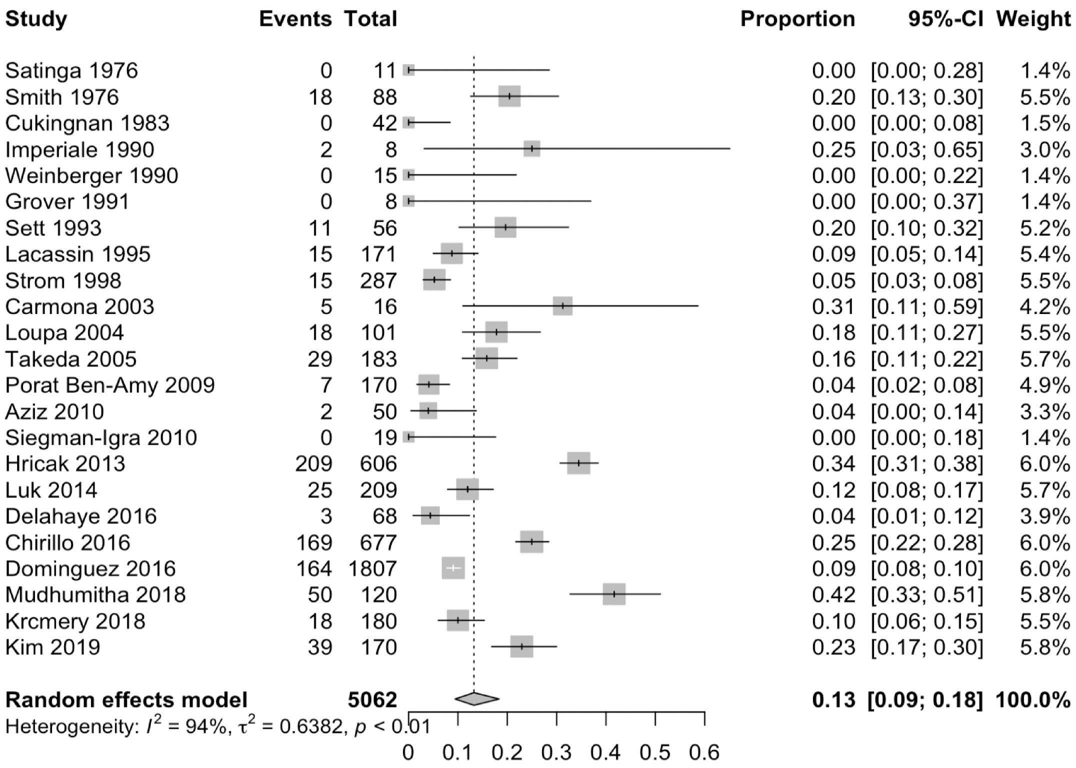

Supplementary Figure 2F. Proportion of culture negative infective endocarditis

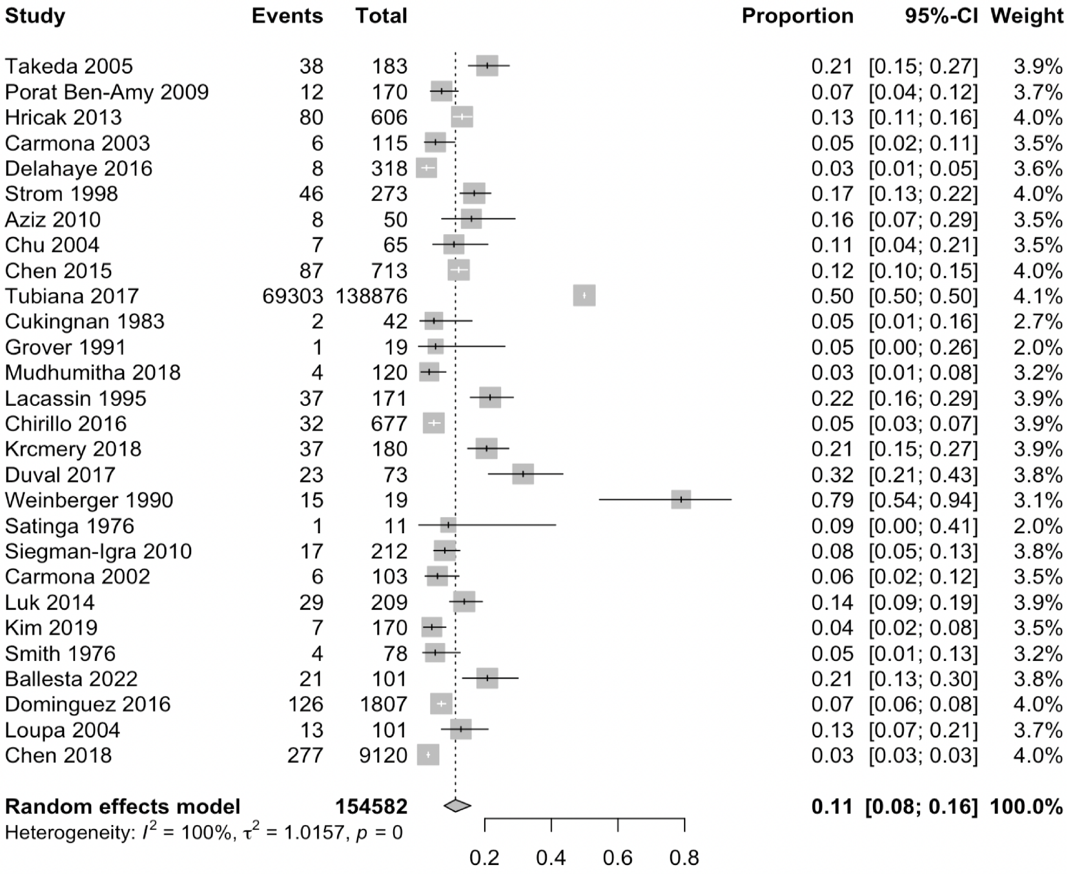

Supplementary Figure 3. Proportion of dental procedures in IE patients

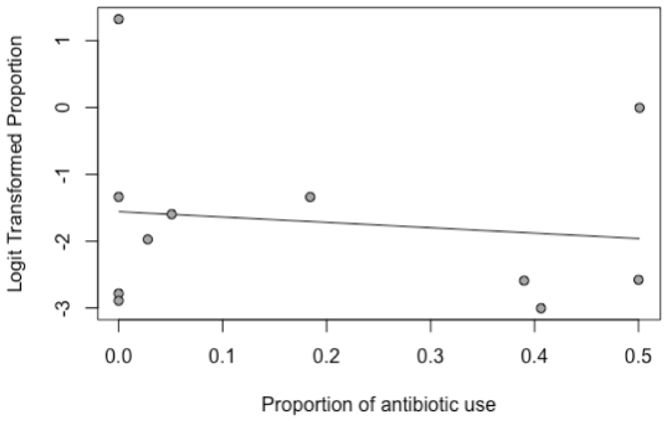

Supplementary Figure 4A: Meta-regression of proportion of infective endocarditis patients with recent dental procedure against proportion of antibiotic use (Intercept coefficient: -0.8091, p=0.6529)

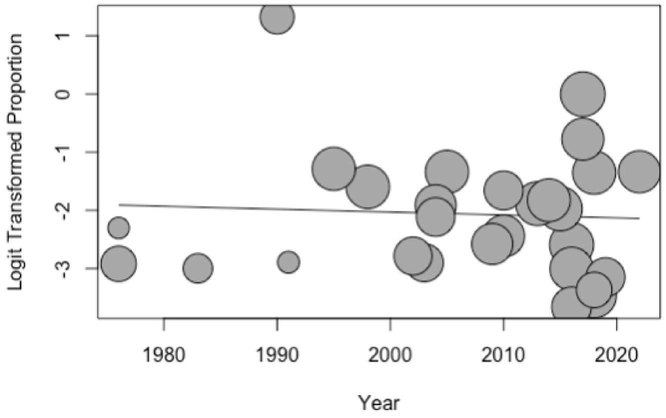

Supplementary Figure 4B: Meta-regression of proportion of infective endocarditis patients with recent dental procedure against the year of publication (Intercept coefficient = -0.005, p=0.7717)

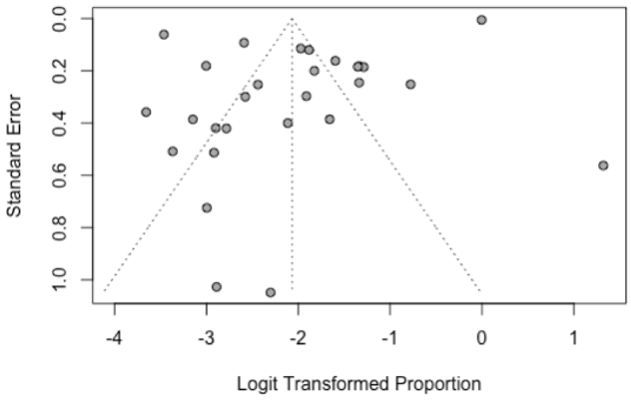

Supplementary Figure 5A: Funnel plot for proportion of IE patients who underwent recent dental procedures (Egger's test: t = -4.46, p = 0.0001)

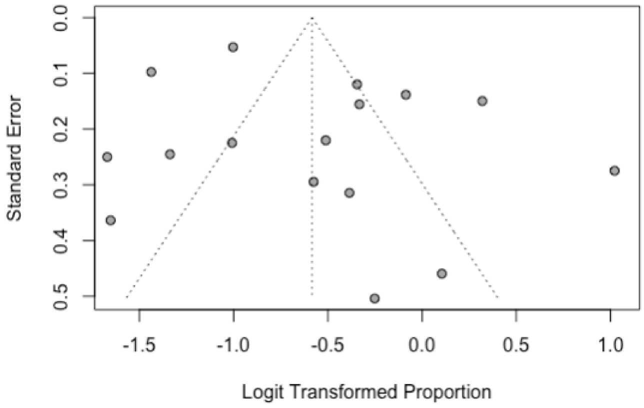

Supplementary Figure 5B: Funnel plot for proportion of Streptococcus (Egger's test: t = 1.36, p = 0.1968)

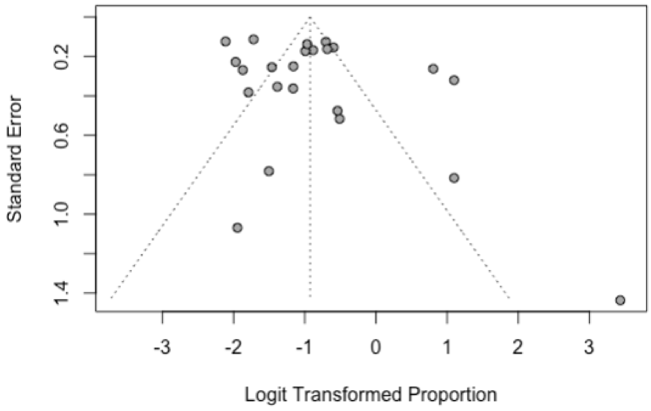

Supplementary Figure 5C: Funnel plot for proportion of Streptococcus Viridans

(Egger's test: t = 1.35, p = 0.1921)

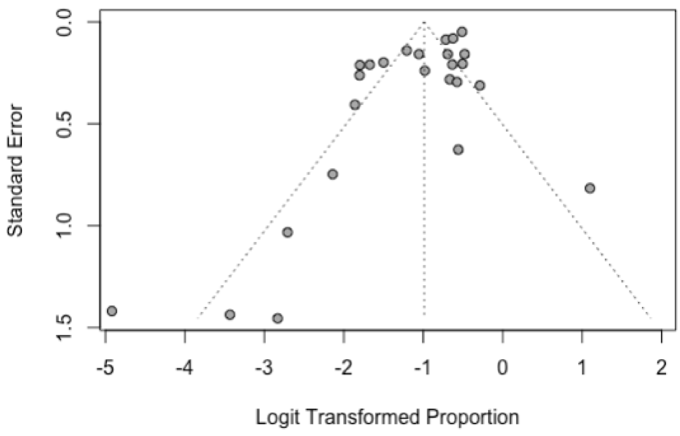

Supplementary Figure 5D: Funnel plot for proportion of Staphylococcus (Egger's test: t = -3.19, p = 0.0040)

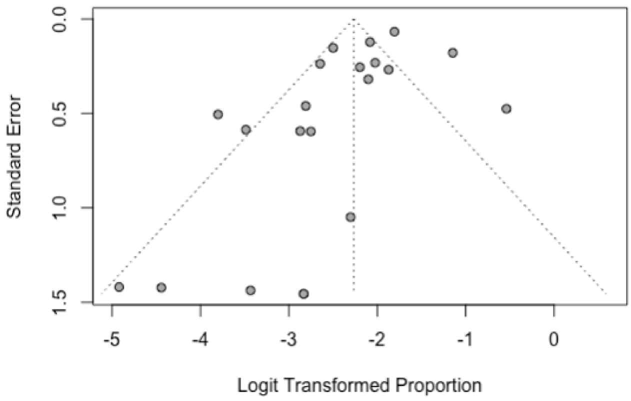

Supplementary Figure 5E: Funnel plot for proportion of Enterococcus (Egger's test: t = -2.40, p-value = 0.0268)

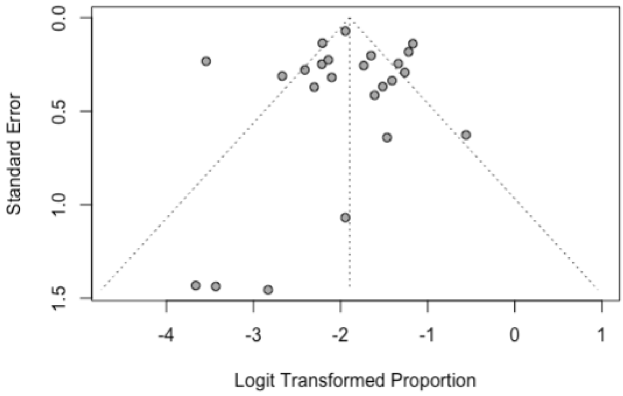

Supplementary Figure 5F: Funnel plot for proportion of HACEK (Egger's test: t = -0.23, p = 0.8239)

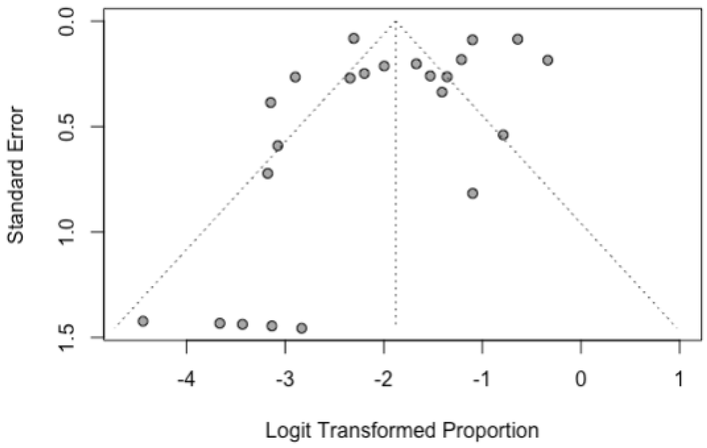

Supplementary Figure 5G: Funnel plot for proportion of culture negative infective endocarditis (Egger's test: t = -1.43, p = 0.1673)
