# Supplementary material for: Prophylactic antibiotic use for infective endocarditis: a systematic review and meta-analysis
Source: BMJ Open. 2023 Aug 22;13(8):e077026. doi: 10.1136/bmjopen-2023-077026 (PMC10445353; doi:10.1136/bmjopen-2023-077026)
Supplement: Author's manuscript [file bmjopen-2023-077026.draft_revisions.pdf]

BMJ Open is committed to open peer review. As part of this commitment we make the peer review history of every article we publish publicly available.

When an article is published we post the peer reviewers' comments and the authors' responses online. We also post the versions of the paper that were used during peer review. These are the versions that the peer review comments apply to.

The versions of the paper that follow are the versions that were submitted during the peer review process. They are not the versions of record or the final published versions. They should not be cited or distributed as the published version of this manuscript.

BMJ Open is an open access journal and the full, final, typeset and author-corrected version of record of the manuscript is available on our site with no access controls, subscription charges or pay-per-view fees (http://bmjopen.bmj.com).

If you have any questions on BMJ Open's open peer review process please email info.bmjopen@bmj.com

Prophylactic antibiotic use for infective endocarditis - a systematic review and meta-analysis

Journal:	*BMJ Open*
Manuscript ID	bmjopen-2023-077026
Article Type:	Original research
Date Submitted by the Author:	26-Jun-2023
Complete List of Authors:	Lean, Sue SH; Wei Gong Memorial Hospital<br>Jou, Eric; University of Cambridge<br>Ho, Jamie; National University Health System<br>Jou, Ernest GL; Wei Gong Memorial Hospital
Keywords:	INFECTIOUS DISEASES, Valvular heart disease < CARDIOLOGY, Cardiology < INTERNAL MEDICINE, IMMUNOLOGY

SCHOLARONE™ Manuscripts

I, the Submitting Author has the right to grant and does grant on behalf of all authors of the Work (as defined in the below author licence), an exclusive licence and/or a non-exclusive licence for contributions from authors who are: i) UK Crown employees; ii) where BMJ has agreed a CC-BY licence shall apply, and/or iii) in accordance with the terms applicable for US Federal Government officers or employees acting as part of their official duties; on a worldwide, perpetual, irrevocable, royalty-free basis to BMJ Publishing Group Ltd ("BMJ") its licensees and where the relevant Journal is co-owned by BMJ to the co-owners of the Journal, to publish the Work in this journal and any other BMJ products and to exploit all rights, as set out in our licence.

The Submitting Author accepts and understands that any supply made under these terms is made by BMJ to the Submitting Author unless you are acting as an employee on behalf of your employer or a postgraduate student of an affiliated institution which is paying any applicable article publishing charge ("APC") for Open Access articles. Where the Submitting Author wishes to make the Work available on an Open Access basis (and intends to pay the relevant APC), the terms of reuse of such Open Access shall be governed by a Creative Commons licence – details of these licences and which Creative Commons licence will apply to this Work are set out in our licence referred to above.

Other than as permitted in any relevant BMJ Author's Self Archiving Policies, I confirm this Work has not been accepted for publication elsewhere, is not being considered for publication elsewhere and does not duplicate material already published. I confirm all authors consent to publication of this Work and authorise the granting of this licence.

For peer review only

Prophylactic antibiotic use for infective endocarditis – a systematic review and meta-analysis

Authors: Sue SH Lean*[,1], Eric Jou*[,2], Jamie SY Ho†[,3], Ernest GL Jou†[,1]

* Both authors contributed equally to this manuscript as co-first authors

† Both authors contributed equally to this manuscript as co-senior authors

[1] Wei Gong Memorial Hospital, Taiwan

[2] Addenbrooke's Hospital, University of Cambridge, United Kingdom

[3] Alexandra Hospital, National University Health System, Singapore

Corresponding author

Dr Eric Jou, MB Bchir (Dist.), MA (Cantab), PhD

Email: ej290@cam.ac.uk

Address: Queens' College Cambridge, CB3 9ET, United Kingdom

Abstract

Objective Infective endocarditis (IE) is a devastating disease with a 50% one-year mortality rate. In recent years, medical authorities across the globe advised stricter criteria for antibiotic prophylaxis in patients with high risk of IE undergoing dental procedures. Whether such recommendations may increase the risk of IE in at-risk patients must be investigated.

Methods and Analysis A systematic search of four databases Medline, Embase, Scopus and Clinictrials.gov was performed to identify eligible studies based on our prospectively registered systematic review and meta-analysis protocol on PROSPERO (CRD42022326664). All studies were assessed by two reviewers independently, and any discrepancies were further resolved through a third researcher.

Results Overall, 11% (95% CI 0.08-0.15, I^2=99.5%) of IE are associated with recent dental procedures. *Streptococcus viridans* accounted for 69% (95% CI 0.46-0.85) of IE in patients who had undergone recent dental procedures, compared to only 21% (95% CI 0.17-0.26) in controls (P=0.003). Subgroup analysis revealed no statistically significant differences in IE associated with dental manipulation across four continents (P=0.99). None of the high risk patients developed IE across all studies where 100% of the patients were treated with prophylactic antibiotics, and IE patients are 12% more likely to have undergone recent dental manipulation compared to matched controls (95% CI 1.00-1.26, P=0.048).

Conclusions Although there is a lack of randomised control trials in the literature on this topic, antibiotic prophylaxis are likely of benefit in reducing the incidence of IE in high-risk patients after dental procedures. Further well-designed randomised controlled trials and high-quality case-controlled studies are required.

For peer review only

Key Words: Infective endocarditis, dental, antibiotic prophylaxis, bacterial endocarditis

Strengths and limitations of this study

- A comprehensive systematic review and meta-analysis interrogating the complex question of the association between dental procedures, infective endocarditis and antibiotic prophylaxis through multiple angles
- The studies included in the present analysis originate from broad geographical locations spanning four continents including North America, Europe, Asia and Australia allowing accurate global representation
- No randomised-controlled trials were included in the present systematic review and meta-analysis due to the lack of such study type in the literature on this important topic

For peer review only

Introduction

Infective endocarditis (IE) is an infection of the inner linings or valves of the heart with a near 100% mortality rate before the advent of antibiotics (1). Mortality remains high despite modern advances in medicine, estimated to be around 25% during in-hospital admissions and almost 50% at one year follow-up (2). Symptoms of IE include pyrexia, chills, rigours, headaches, dyspnoea, cough, myalgia, arthralgia and general fatigue, and in addition to antibiotics up to 50% of patients may require surgical treatment (3).

Mechanistically, IE occurs due to injury to the heart lining or valve, allowing pathogens transiently travelling in the blood to lodge. Patients at high risk of IE include those with previous or active heart diseases which increases the susceptibility of pathogen colonisation on injured heart tissue, in accordance with the American Heart Association (AHA) and European Society of Cardiology (ESC) guidelines (4). Importantly, dental procedures are associated with bacteraemia (5, 6), and antibiotic prophylaxis have long been used for dental procedures across the world in at-risk patients to reduce IE incidence. Nevertheless, there has been uncertainties on the efficacy of antibiotic prophylaxis (7), and in 2008 the United Kingdom (UK) National Institute for Health and Care Excellence (NICE) clinical guidelines were updated advising against their routine use in the prevention of IE in patients undergoing dental procedures. This is in contrast to most countries globally including the United States (US) and many European countries where antibiotic prophylaxis remains in-use, although there is a tendency towards stricter criteria over the years (8, 9). On one hand, withholding antibiotic prophylaxis where there are no foreseeable benefits is cost effective and can prevent overtreatment and side-effects. However, given that IE carries significant morbidity and mortality, it remains vital to ascertain whether measures to reduce or completely

For peer review only

withhold antibiotic prophylaxis may lead to a higher incidence of IE secondary to dental procedures.

Despite the long history of antibiotic prophylaxis in IE prevention, there remains to be no clear evidence to support or refute their use due to the lack of high-quality study or trials (7, 10, 11). This is further complicated by most studies utilising bacteraemia rather than IE as an endpoint as highlighted in recent systematic reviews (12). In this article, we perform a systematic review and meta-analysis of the literature exploring the aetiology and underlying microbial causes of dental procedure-related IE, followed by assessing the association between IE incidence and dental manipulation. Focus is placed on analysing the prevalence of recent preceding dental manipulation in patients diagnosed with IE in relation to prophylactic antibiotic use. Furthermore, studies reporting IE incidence and outcome in high risk patients whom had undergone recent dental procedures are also interrogated. Finally, based on our findings we comment on the rationale and efficacy of prophylactic antibiotic use in high-risk patients in the prevention of IE.

Materials and Methods

The systematic review and meta-analysis protocol was prospectively registered on PROSPERO (CRD42022326664). Patients or the public WERE NOT involved in the design, or conduct, or reporting, or dissemination plans of our research. The main clinical question interrogated in this systematic review and meta-analysis is to assess the potential association of dental procedures and infective endocarditis, and secondarily whether prophylactic

For peer review only

antibiotic use may be of benefit in IE prevention relating to dental procedures. Due to the lack of randomised-controlled trials in the literature, we attempted to answer this pressing question through utilising a multifaceted approach.: Firstly, we assessed the potential association between IE and recent dental procedures through the inclusion of studies where incidence of recent dental procedures were reported in IE patients. Additionally, assessments comparing the causative organisms of IE associated with recent dental procedures to general IE cases were also performed in studies where such data is reported. Furthermore, studies where patients at risk of IE were treated with prophylactic antibiotics for dental procedures were also interrogated to assess for any association between antibiotic prophylaxis and IE prevention.

Study selection

A systematic search of four databases namely Medline, Embase, Scopus and Clinictrials.gov was performed from the date of inception to 23 May 2022, with the search terms "dent*" OR "*dontic*" AND "endocarditis". References of included studies and relevant reviews were hand searched for additional articles. Inclusion criteria were primary studies involving adults, reporting infective endocarditis within 3 months of dental procedures. Studies that reported dental sources, but not specifically dental procedures were excluded. Conference abstracts, reviews, case reports and case series involving fewer than 10 cases and non-English studies

For peer review only

were excluded. Title and abstracts were screened by two reviewers independently, and full texts were subsequently reviewed for included studies and those with discrepancies. Conflicts were resolved by discussion and involvement of a third researcher. Search strategy (Supplementary Table 1) and quality assessment of included studies (Supplementary Table 2) are detailed in the supplementary materials accordingly.

Data extraction

Data were extracted onto a standardized form by two researchers independently, which included the country of publication, study design, population, type of dental procedure, proportion of infective endocarditis, organism identified on blood culture, use of prophylactic antibiotics and outcomes.

Statistical analysis

Statistical tests were performed using R (R Core Team (2021)), based on the guidelines outlined in the Cochrane Handbook. Meta-analysis of IE and dental procedure proportions was performed using the random-effects model, and subgroup analysis was performed based on the region of publication, year of publication and proportion of patients with antibiotic prophylaxis. The type of bacterial organisms isolated from blood cultures were also analysed by meta-analysis of proportions. Between-study heterogeneity was presented using the I^2 statistic. A p value of <0.05 was considered statistically significant.

Results

For peer review only

A total of 3771 articles were identified after removal of duplicates, 3272 articles were excluded at the title and abstract screening stage as they were not relevant to the topic, and a final total of 38 articles satisfied the inclusion criteria and were included in this review (Supplemental Figure 1).

Infective endocarditis aetiology and causative organisms

The development of infective endocarditis follows a two-step process whereby initial damage to the endocardium provides a nidus allowing pathogen settlement and colonisation following a period of transient bacteraemia or fungemia. Studies indicate that bacteria are the most common pathogen class responsible for IE (13, 14). Common oral commensals or pathogens that cause infective endocarditis include *Streptococcus viridans* (15) and those of the HACEK (*Haemophilus*, *Aggregatibacter*, *Cardiobacterium*, *Eikenella*, and *Kingella*) group (16).

In this systematic review, 26 studies reported the microbial species responsible for the IE cases (Table 1). Pooled analysis revealed that *Staphylococcus* species accounted for 23% (95% CI 0.18 to 0.30, I^2 = 83.2%) of IE cases overall (Supplementary Figure 2A), while *Streptococcus* species accounted for 36% (95% CI 0.28 to 0.44, I^2 = 93.7%) (Supplementary Figure 2B). *Streptococcus viridans* and *Enterococcus* are responsible for 30% (95% CI 0.22 to 0.40, I^2 = 91.7%) (Supplementary Figure 2C) and 7% (95% CI 0.05 to 0.11, I^2 = 76.6%) (Supplementary Figure 2D) of total IE cases respectively. 12% (95% CI 0.10 to 0.16, I^2 = 82.5%) of all IE cases are due to HACEK and other organisms (Supplementary Figure 2E), while in another 11% (95% CI 0.07 to 0.16, I^2 = 93.6%) (Supplementary Figure 2F) the causative organism could not be identified.

For peer review only

20 of the 26 studies presented pooled data from all IE patients regardless of cause, while the other six reported exclusively the causative microorganism in IE patients who received recent dental procedures (Table 1). The majority of the 20 studies with pooled data (12 out of 20; 60%) found *Staphylococcus* to be the most common cause of IE, ranging from 14% to 75% of patients. This is closely followed by *Streptococcus* species collectively being the dominant cause in seven out of the 20 pooled studies (35%), ranging from 27.5% to 57%. Of the *Streptococcus* species found in these studies, *Streptococcus viridans* are the most common and are likely associated with the subgroup that received prior dental treatment. Other *Streptococcus* species identified include *Streptococcus bovis* and *Strepnococcus pneumonia* which account for the remaining cases. Of the six studies where the causative organisms of IE were portrayed specifically in patients whom received prior dental procedures, *Streptococcus* species were the most common (Table 1). *Streptococcus viridans* accounted for the majority, ranging from 23.9% and up to 100% of the total IE cases. HACEK and other organisms tend to be the next most common, while *Staphylococcus* is rare unlike in the general population. Subgroup meta-analysis comparing studies reporting microbial causes of IE exclusively in patients who had undergone recent dental procedures compared to general IE cases revealed important differences in the underlying cause of IE (Table 1). In those with prior dental procedures, *Streptococcus viridans* are responsible for a significantly higher proportion at 69% (95% CI 0.46 to 0.85; $I^2 = 63.7\%$) of cases, compared to 21% (95% CI 0.17 to 0.26; $I^2 = 89.0\%$) in general IE patients ($P = 0.003$). Conversely, those with recent dental procedures are less likely ($P = 0.002$) to suffer from *Staphylococcus* IE at only 3% (95% CI 0.01 to 0.14, $I^2 = 0\%$) compared to 29% in general IE patients (95% CI 0.25 to 0.34, $I^2 = 85.9\%$).

For peer review only

Proportion of infective endocarditis cases associated with dental procedures

33 studies assessed the proportion of IE patients who had recently undergone dental procedures within three months (Table 2), with cohort sizes ranging from 11 to 138,876 patients (17, 18). The most common dental procedures reported include tooth extraction, fillings, scaling and oral surgeries, although most studies did not specify (21 out of 33, 64%). The proportion of patients whom had undergone recent dental procedures, which are considered to be the most likely cause of the IE in these patients, varied widely between studies (2.5% to 49.9%). This amounted to an overall 11% (95% CI 0.08 to 0.15, $I^2 = 99.5\%$) (Supplementary Figure 3) incidence of IE patients whom had received recent dental procedures. Subgroup analysis by continent including North America, Europe, Asia and Australia revealed no differences in the proportion of IE patients with recent dental procedures (P = 0.99) (Table 1).

Proportion of patients that received dental procedures in case control studies

We further analysed the included case-control studies to assess for incidence of recent dental treatment in IE patients compared to controls without IE, which amounted to a total of six eligible studies including both conventional case-control along with case-crossover studies. Intriguingly, whilst none of the individual studies showed a statistically significant difference in the proportion of recent dental procedures comparing IE cases to controls, pooled meta-analysis revealed that IE patients are more likely to have received recent (within three months) dental treatment compared to matched controls (Relative Risk (RR) = 1.12; 95% CI 1.00 to 1.26, $I^2 = 0\%$, P = 0.048) (Figure 1). These findings support the notion that recent dental procedures may be associated with higher IE incidence.

For peer review only

Infective endocarditis incidence in patients who underwent dental procedures

Five eligible studies investigated IE incidence in patients after receiving recent dental procedures (Table 3). All of these are cohort studies, with three being retrospective while two prospective. One retrospective study with 12,102 systemic lupus erythematosis(SLE) patients from Taiwan did not mention the proportion of patients that received prophylactic antibiotics (19). In that study, Cox multivariate proportional hazard analysis showed that recent dental procedures is an independent risk factor for IE (HR = 36.8, P < 0.001). The remaining four studies each reported that 100% of their patients received prophylactic antibiotics, consistent with their study design as all patients were at high-risk of IE (Figure 2). In an Italian prospective cohort study of 104 patients with prosthetic heart valves, no patients developed IE three months after the dental surgery (20). Another prospective cohort study (Israel) involving 90 patients with prosthetic heart valve or rheumatic heart disease similarly found no cases of IE after dental procedures (scaling, fillings, extraction, root canal, crowns and bridges) (21). In that study, the striking zero IE cases observed following an optimal protocol of administering appropriate prophylactic antibiotics proven to be efficacious against *Streptococcus viridans*, is significantly lower than what would be expected statistically. This suggests that appropriate antibiotic prophylaxis may reduce IE incidence. Similarly, the remaining two retrospective cohort studies both found no cases of IE following dental procedures in high-risk patients (22, 23). In one study, 13 patients received dental implants with no cases of IE at two-year follow-up (22). In the other study where 206 patients were assessed, whilst none of them developed endocarditis, a minority (13.1%) developed mild side effects from prophylactic amoxicillin administration for dental procedures (extraction, abscess drainage, oral surgery, root canal, scaling, crowns and bridges) (23). In that study, the most common side effects were gastrointestinal, including abdominal discomfort (11 patients; 5.3%), nausea (6 patients; 2.9%) and constipation (5 patients; 2.4%). Overall, the severity of

For peer review only

these side effects was classified as very mild, and patients showed a high degree of compliance to the prophylactic treatment. Collectively, these studies suggest that whilst recent dental procedures are independently associated with IE risk, prophylactic antibiotic use may be beneficial in reducing IE incidence.

Discussion

In this systematic review and meta-analysis, we address the critical question of whether current antibiotic prophylaxis regimes are effective and of benefit in reducing IE incidence in high-risk patients. This is particularly crucial in an era where infective endocarditis incidence continues to rise globally (24).

We found *Streptococcus* species to be the most common cause of IE followed by *Staphylococcus* upon pooled analysis of 28 eligible studies with suitable data. This differs from the current consensus where modern studies found *Staphylococcus* to be the leading cause of IE in the general population (25). The overrepresentation of *Streptococcus* IE in our systematic review is likely a reflection of the included studies typically involving dental procedures. Accordingly, our meta-analysis found that microbial causes of IE is significantly different in IE patients who had undergone recent dental procedures compared to general IE cases, with a striking almost 70% prevalence of *Streptococcus viridans* IE in the former group. Conversely, we found that patients who received prior dental manipulation are much less likely to suffer from *Staphylococcus* IE at only 3% compared to 29% of the general IE patients. Other studies have similar found *Staphylococcus aureus* to account for approximately 30% of overall IE cases (26). The association of *Streptococcus viridans* with post dental procedure IE observed here is consistent with the former being a common cause

For peer review only

of odontogenic diseases (27). These findings suggest that any prophylactic antibiotic use for dental procedures should be tailored to best protect against *Streptococcus viridans* to ensure maximal efficacy. Importantly, whilst amoxicillin is commonly used for antibiotic prophylaxis prior to dental procedures in at-risk patients, studies have shown *Streptococcus viridans* to be resistant to amoxicillin to differing degrees, and may contribute to the lack of efficacy of prophylactic antibiotics found in some studies (28, 29).

Gram negative organisms, in particular the oral commensals of the HACEK group, remain as important causes of IE despite accounting for only a small proportion of cases compared to gram positive cocci *Streptococcus* and *Staphylococcus* (16). Our systematic review showed that many studies in the literature do not report HACEK organisms as a separate categorical cause of IE to other miscellaneous pathogens. Future studies segregating HACEK organisms from other bacterial causes of IE will shed insight on whether prophylactic antibiotics use is efficacious in preventing HACEK IE specifically.

We found that segregating studies into two groups defined as above or below average use of prophylactic antibiotics for dental procedures revealed that increased prophylactic antibiotic use may reduce IE from 18% to 11.5% although this was not statistically significant (P = 0.49). Our findings cannot rule out antibiotics as important prophylactic treatment in reducing IE incidence in high-risk patients, as only a total of 11 eligible studies had suitable data for analysis, and as such our meta-analysis is likely underpowered. Furthermore, most studies did not report the proportion of patients that are deemed high-risk in their respective cohorts, and therefore studies with low prophylactic antibiotic usage may indeed have covered for all high-risk patients. In fact, our meta-analysis on all eligible studies investigating IE incidence

For peer review only

after dental treatment with appropriate antibiotic cover in 100% of patients revealed a striking 0% incidence of IE. More studies are required to increase the sample size which will allow future meta-analyses to reach sufficient power to draw further conclusions.

Our meta-analysis of the six eligible studies with suitable data eliciting a pooled total of 11,095 cases and the same number of matched controls showed that IE patients are significantly more likely to have undergone recent dental procedures (RR = 1.12, 95% CI 1,00 to 1.26, $I^2 = 0\%$). We have selected 3 months as the cut off for recent dental procedures due to the relatively long incubation period and prolonged duration of symptoms of IE, in accordance with the majority of previously published studies (Table 2). These findings support the notion that dental procedures may increase the risk of IE and argue for antibiotic prophylaxis. As many of these studies utilised a case-crossover design (self-controls), this is unlikely to be due to other existing comorbidities. Indeed, studies assessing the incidence of IE before and after the 2007 American Heart Association (AHA) guideline changes found the sharp decline in prophylactic antibiotic use to be associated with a significant increase in IE incidence post dental procedures (30). Dental procedures such as tooth extraction have been shown to cause bacteraemia in 100% of adults (5, 6). Studies in the UK interrogating the incidence of IE after the 2008 NICE guideline changes which advised against routine prophylactic antibiotic use for dental procedures in at-risk patients similarly found an increase in incidence of oral streptococcus IE but not overall IE (31). This is consistent with our findings where oral streptococcus, mainly consisting of *Streptococcus viridans* group, are the major cause of IE after dental procedures whereas *Staphylococcus* species are the more common cause in general IE patients.

For peer review only

Recently, three important studies investigating IE, dental procedures and antibiotic prophylaxis were published (32, 33, 34) after the inclusion deadline of the present systematic review and meta-analysis as per prospectively registered on PROSPERO. In a US case-crossover study of 7,951,972 patients with employer-provided health cover, a strong temporal association was found between invasive dental procedures in the preceding four weeks and IE in high-risk patients, with an odds ratio (OR) of 2.00 (95% CI 1.59 to 2.52, P = 0.002) (32). The association was strongest with certain dental procedures including dental extractions (OR = 11.08, 95% CI 7.34 to 16.74, P < 0.0001) and oral-surgical procedures (OR = 50.77, 95% CI 20.79 to 123.98, P < 0.0001). Strikingly, in that study the author also found that prophylactic antibiotic administration reduced IE incidence after dental procedures (OR = 0.49, 95% CI 0.29 to 0.85, P = 0.01) including dental extractions (OR = 0.13, 95% CI 0.03 to 0.34, P < 0.0001) and oral-surgical procedures (OR = 0.09, 95% CI 0.01 to 0.35, P = 0.002), particularly in high-risk as opposed to moderate or low-risk patients (32). In another elegant case-crossover study of 1,678,190 Medicaid patients (basic medical and dental cover), similar findings were observed where recent dental extractions (OR = 14.17, 95% CI 5.40 to 52.11, P < 0.0001) or oral surgery (OR = 29.98, 95% CI 9.62 to 119.34, P < 0.0001) within 30 days is associated with increased IE incidence, and that prophylactic antibiotic administration for dental procedure reduced IE incidence (OR = 0.20, 95% CI 0.06 to 0.53, P < 0.0001) (33). The number needed to treat with antibiotic prophylaxis to prevent one case of IE was estimated to range between 71 to 244 depending on the type of dental procedure. Finally, in a UK-based study of 14,731 patients by the same group investigating various different procedures and IE, dental procedures including extraction and surgical tooth removal were found to be associated with increased IE risk (OR = 2.14, 95% CI 1.22 to 3.76, P = 0.047) (34). Overall, these findings are largely consistent with our systematic review and meta-analysis, where IE patients are associated with increased risk of having undergone recent

For peer review only

dental procedures in the preceding three months (RR = 1.12; 95% CI 1.00 to 1.26, I^2 = 0%, P = 0.048), and that across all the studies where 100% of the patients received antibiotic prophyalxis, none of the high risk patients developed IE. These results indicate that there is an association between dental procedures and IE, and support the aforementioned AHA and ESC guidelines advocating the use of antibiotic prophylaxis in high-risk patients for dental procedures.

Limitations

Whilst our systematic review and meta-analysis provide an important appraisal and synthesis of all the available evidence on the association of dental procedures and IE in the context of prophylactic antibiotic use, there are important limitations. These include the differing patient characteristics between studies which may contribute to inter-study heterogeneity. Due to the update in AHA and ESC guidelines in 2007 and 2009 respectively, the use of prophylactic antibiotics may differ between studies performed in different years, which may confound the association of invasive dental procedures and IE. Further limitations include the lack of randomised controlled trials available in the literature and therefore studies included in this systematic review and meta-analysis mainly consist of observational cohort studies and surveys. Due to the rarity of IE, a large sample size is required to allow sufficient statistical power to detect any potential association, if any, between prophylactic antibiotic use for recent dental procedures and IE incidence. Indeed, whilst none of the individual included studies found a statistically significant difference in the incidence of recent dental procedures in IE patients compared to controls likely due to being underpowered, our meta-analysis demonstrated that IE patients are more likely to have undergone recent dental manipulation. The efficacy of prophylactic antibiotics, shown by some studies to be 100% in our meta-

For peer review only

analysis, may further hide the association between IE and recent dental procedures. Another potential limitation is that studies performed in different times may record the casual organism differently, due to changes in classification of some types of bacteria, particularly enterococci and HACEK organisms. Furthermore, whilst some studies directly assessed dental records when gathering data on dental procedures, others relied on self-report by patients which is prone to recall bias. Similarly for data on prophylactic antibiotic use, some studies utilised patient records while others may rely on patient recollection or simply assume that all high-risk patients would have been offered antibiotic prophylaxis, therefore may be difficult to accurately verify.

Conclusions

Overall, our findings in this systematic review and meta-analysis indicate that prophylactic antibiotics for dental procedures may be of benefit in preventing IE in high-risk patients. Given the logistic difficulties of conducting randomised control trials for prophylactic antibiotic use and IE, further well-designed cohort studies will be invaluable to address this very important issue.

For peer review only

Declarations

Funding

Not applicable.

Conflicts of interest/Competing interests

The authors declare no conflicting interest.

Ethics approval

Not applicable.

Consent to Participate

Not applicable.

Consent for publication

Not applicable.

Author contributions

S.S.H.L, E.J, J.S.Y.H, and E.G.L.J were involved in the conceptualization of the study, designed the study, collected and analysed the data. All authors contributed to writing the manuscript, and approved for the final version of the paper to be published.

For peer review only

Outcome	Number of studies	N	Proportion (95% CI)	I^2 value	P-value
**Dental procedures in IE patients**	**28**	**154582**	**0.11 (0.08-0.15)**	**99.5%**	**-**
Year of patient recruitment					0.372
Before 2007	19		0.12 (0.09-0.17)	80.1%	
After 2007	9		0.09 (0.04-0.18)	99.8%	
Continent of publication					0.989
Asia	9		0.10 (0.04-0.22)	97.7%	
North America	6		0.12 (0.08-0.17)	55.8%	
Europe	12		0.12 (0.07-0.19)	99.2%	
Australia	1		0.11 (0.05-0.21)	-	
**Streptococcus bacteraemia**	**16**	**3994**	**0.36 (0.28-0.44)**	**93.7%**	**-**
Dental procedures proportion					0.003
All patients had dental procedures	3		0.61 (0.43-0.76)	68.6%	
Not all patients had dental procedures	13		0.32 (0.25-0.39)	93.7%	
**Streptococcus viridans bacteraemia**	**23**	**3363**	**0.30 (0.22-0.40)**	**91.7%**	**-**
Dental procedures proportion					0.003

For peer review only

All patients had dental procedures	6		0.69 (0.46-0.85)	63.7%	
Not all patients had dental procedures	17		0.21 (0.17-0.26)	89.0%	
**Staphylococcus bacteraemia**	**25**	**5215**	**0.23 (0.18-0.30)**	**83.2%**	**-**
Dental procedures proportion					0.002
All patients had dental procedures	6		0.03 (0.01-0.14)	0%	
Not all patients had dental procedures	19		0.29 (0.25-0.34)	85.9%	
**Enterococcus bacteraemia**	**21**	**4767**	**0.07 (0.05-0.11)**	**76.6%**	**-**
Dental procedures proportion					0.355
All patients had dental procedures	4		0.003 (0.00-0.79)	0%	
Not all patients had dental procedures	17		0.08 (0.06-0.11)	79.0%	
**HACEK bacteraemia**	**24**	**5163**	**0.12 (0.10-0.16)**	**82.5%**	**-**
Dental procedures proportion					0.385
All patients had dental procedures	5		0.05 (0.004-0.36)	0.0%	
Not all patients had dental procedures	19		0.13 (0.10-0.16)	85.8%	
**Negative bacteraemia**	**23**	**5062**	**0.11 (0.07-0.16)**	**93.6%**	**-**
Dental procedures proportion					0.411

For peer review only

All patients had dental procedures	5		0.06 (0.01-0.25)	54.7%	
Not all patients had dental procedures	18		0.12 (0.08-0.18)	94.9%	
**IE in high-risk patients who underwent dental procedures**	**4**	**413**	**0.00 (0.00-1.00)**	**0%**	**-**

Table 1: Results of the meta-analyses performed with subgroup analysis

For peer review only

Author, Year, Country	Study type	Population	Sample size	Type of dental procedure	Dental procedure (%)	Prophylactic abx (%)	Study duration	Duration between dental procedure and IE
Takeda et al., 2005, Japan	Retrospective cohort	CHD with IE	183	Not specified	21.00%	18.40%	1971-1998	NR
Durack et al., 1983, US	Case series	Antibiotic prophylaxis failure, IE post dental procedures	52	Not specified	92.00%	100%; 12% had AHA recommended regimen	1979	5 weeks (median)
Porat Ben-Amy et al., 2009, Israel	Retrospective case-crossover	IE	170	Tooth extraction, periodontal treatment, root canal treatment, implant insertion, others	12.00%	50% eligible patients	2003-2005	3 months
Hricak et al., 2013, Slovakia	Prospective cohort	IE	606	Not specified	13.20%	NR	1984-2006	NR

For peer review only

Carmona et al., 2003, Spain	Retrospective cohort	IE	115	Tooth extraction, Fillings, Scaling, Professional cleaning	5.20%	NR	1997-2001	3 months
Delahaye et al., 2016, France	Prospective cohort	IE	318	Not specified	2.50%	NR	2005-2016	3 months
Strom et al., 1998, US	Population-based case-control study	Case - IE; control - no IE	273 case 273 control	Dental hygiene care, filling periodontal treatment, restorative dentistry, extraction, endodontic treatment, treatment of tooth abscess, mouth or gingival surgery, other	16.8% in cases; 14.3% in controls	Cases: 5.1%; Controls: 1.1%	1988-1990	2 months
Aziz et al., 2010, US	Retrospective cohort	IE undergoing heart surgery	50	Not specified	16.00%	NR	2000-2003	NR

For peer review only

Chu et al., 2004, New Zealand	Retrospective cohort	IE	65	Not specified	10.80%	NR	1997-2002	NR
Chen et al., 2015, Taiwan	Population-based case-control study	IE	713	Tooth extraction, surgery, dental scaling, periodontal treatment, and endodontic treatment	Cases: 12.2%; controls: 10.8%	2.80%	1999-2012	3 months
Tubiana et al., 2017, France	Population-based cohort and case-crossover study	Positioning or replacement of prosthetic heart valves, with IE	138,876	Invasive and non-invasive	49.9%; 26.0% of procedures were invasive	50.1% eligible patinets	2008-2014	3 months
Imperiale et al., 1990, US	Case-control study	Case - IE after dental procedure with high risk cardiac lesion; control - no IE after dental procedure	8 cases 24 controls	Cleaning, filing, extraction	NA	Cases: 12.5%; Controls 62.5%	1980-1986	3 months

For peer review only

Sett et al., 1993, Canada	Retrospective cohort	IE in procine bioprothesis patients	56	Not specified	NR	NR	1975-1988	NR
Cukingnan et al., 1983, US	Retrospective cohort	Early valve replacement for IE	42	Not specified	5.00%	NR	1969-1983	NR
Grover et al., 1991, India	Retrospective cohort	IE	19	tooth extraction	5.30%	0%	1982-1989	NR
Mudhumitha et al., 2018, India	Retrospective cohort	IE	120	Not specified	3.30%	NR	2010-2015	3 months
Lacassin et al., 1995, France	Case-control study	IE	171 cases, 171 controls	Not specified	Cases: 22%; controls: 19%	NR	1990-1991	3 months
Duval et al., 2006, France	Retrospective survey	General population	2805	Not specified	0.70%	26.30%	1998	1 month

For peer review only

Chirillo et al., 2016, Italy	Prospective cohort	IE	677	Not specified	4.70%	40.60%	2007-2010	2 months
Krcmery et al., 2018, Slovakia	Prospective cohort	IE	180	Not specified	20.50%	NR	1984-2017	NR
Duval et al., 2017, France	Case-control study	Case - IE caused by oral streptococci; Control - IE caused by nonoral pathogens	73 cases, 192 controls	Not specified	8.80%	NR	2008-2013	3 months
Weinberger et al., 1990, Israel	Retrospective cohort	IE patients with mitral valve prolapse	19	Not specified	78.90%	0%	1970-1987	2 months
Martin et al., 2007, UK	Case series	IE cases after dental procedure with successful litigation	83	Exodontia, scaling, endodontics and minor oral surgery	NA	16.90%	1983-2005	Mean 9 days (range 2-22 days)
Santinga et al., 1976, US	Case series	IE and prosthetic heart valve	11	Not specified	9.10%	NR	1976	NR

Siegman-Igra et al., 2010, Israel	Prospective cohort	Culture-positive IE	212	Not specified	8.00%	NR	1995-1998; 2003-2005	3 months
Carmona et al., 2002, Spain	Retrospective cohort	IE	103	extraction, scaling, filling	5.80%	0%	1997-1999	NR
Luk et al., 2014, Canada	Retrospective cohort	IE treated with surgical valve explant	209	Not specified	13.90%	NR	2001-2012	NR
Kim et al., 2019, Korea	Retrospective cohort	IE in cancer and non-cancer	170	Not specified	4.10%	NR	2011-2015	NR
Smith et al., 1976, UK	Retrospective cohort	IE	78	Not specified	5.10%	NR	1969-1972	NR
Ballesta et al., 2022, Spain	Retrospective cohort	IE	101	Not specified	21.00%	0%	2000-2017	NR

For peer review only

Dominguez et al., 2016, Spain	Prospective cohort	IE	1807	Not specified	7.00%	38.50%	2008-2013	NR
Loupa et al., 2004, Greece	Prospective cohort	IE	101	Not specified	13.00%	NR	1997-2000	3 months
Chen et al., 2018, Taiwan	Case series	IE	9120	dental cleaning, scaling and root planing, simple extraction, complicated extraction, odontectomy in both simple case and complicated case, and periodontal surgery	Cases: 2.7%; controls: 2.7%	NR	2004-2013	3 months

Table 2: Characteristics of included studies on patients with infective endocarditis

For peer review only

Author, year country	Study type	Population	Sample size	Type of dental procedure	IE (%)	Prophylactic antibiotics (%)	Study duration	Duration between dental procedure and IE
Russo et al., 2000, Italy	Prospective cohort	Prosthetic heart valve patients undergoing dental procedures	104	NR	0	100%	NR	3 months
Chang et al., 2017, Taiwan	Retrospective cohort	SLE patients	12102	NR	NR	NR	NR	30 days
Findler et al., 2014, Israel	Retrospective cohort	high risk for IE and underwent dental implant placement	13	All had dental implants	0	100%	1995-2012	NA
Tzukert et al., 1986, Israel	prospective cohort	Prosthetic heart valves, high risk IE	90	scaling, fillings, extraction, root canal, crowns, bridges	0	100%	1967-1982	NA
Littner et al., 1986, Israel	Retrospective cohort	Susceptible patients to IE	206	extraction, drainage of abscess, biopsy, filling, oral surgery, crown, bridge, scaling	0	100%	1982-1986	NA

Table 3: Characteristics of studies on patients undergoing dental procedures

For peer review only

References

1. Hsu CN, Wang JY, Tseng CD, Hwang JJ, Hsueh PR, Liau CS. Clinical features and predictors for mortality in patients with infective endocarditis at a university hospital in Taiwan from 1995 to 2003. Epidemiol Infect. 2006;134(3):589-97.
2. Muñoz P, Kestler M, De Alarcon A, Miro JM, Bermejo J, Rodríguez-Abella H, et al. Current Epidemiology and Outcome of Infective Endocarditis: A Multicenter, Prospective, Cohort Study. Medicine (Baltimore). 2015;94(43).
3. Rajani R, Klein JL. Infective endocarditis: A contemporary update. Clin Med (Lond). 2020;20(1):31-5.
4. Richey R, Wray D, Stokes T. Prophylaxis against infective endocarditis: summary of NICE guidance. BMJ. 2008;336(7647):770-1.
5. Lockhart PB. The risk for endocarditis in dental practice. Periodontol 2000. 2000;23:127-35.
6. Barbosa M, Prada-López I, Álvarez M, Amaral B, de los Angeles CD, Tomás I. Post-tooth extraction bacteraemia: a randomized clinical trial on the efficacy of chlorhexidine prophylaxis. PLoS One. 2015;10(5):e0124249.
7. Cahill TJ, Prendergast BD. Current controversies in infective endocarditis. F1000Res. 2015;4.
8. Embil JM, Chan KL. The American Heart Association 2007 endocarditis prophylaxis guidelines: a compromise between science and common sense. Can J Cardiol. 2008;24(9):673-5.
9. Habib G, Lancellotti P, Antunes MJ, Bongiorni MG, Casalta J-P, Del Zotti F, et al. 2015 ESC Guidelines for the management of infective endocarditis: The Task Force for the Management of Infective Endocarditis of the European Society of Cardiology (ESC)Endorsed by: European Association for Cardio-Thoracic Surgery (EACTS), the European Association of Nuclear Medicine (EANM). European Heart Journal. 2015;36(44):3075-128.
10. Daly CG. Antibiotic prophylaxis for dental procedures. Aust Prescr. 2017;40(5):184-8.
11. Rutherford SJ, Glenny AM, Roberts G, Hooper L, Worthington HV. Antibiotic prophylaxis for preventing bacterial endocarditis following dental procedures. Cochrane Database Syst Rev. 2022;5(5):Cd003813.
12. Cahill TJ, Harrison JL, Jewell P, Onakpoya I, Chambers JB, Dayer M, et al. Antibiotic prophylaxis for infective endocarditis: a systematic review and meta-analysis. Heart. 2017;103(12):937-44.
13. Barnett R. Infective endocarditis. Lancet. 2016;388(10050):1148.
14. Chambers ST, Murdoch D, Morris A, Holland D, Pappas P, Almela M, et al. HACEK infective endocarditis: characteristics and outcomes from a large, multi-national cohort. PLoS One. 2013;8(5):e63181.
15. Birlutiu V, Birlutiu RM, Costache VS. Viridans streptococcal infective endocarditis associated with fixed orthodontic appliance managed surgically by mitral valve plasty: A case report. Medicine (Baltimore). 2018;97(27):e11260.
16. Khaledi M, Sameni F, Afkhami H, Hemmati J, Asareh Zadegan Dezfuli A, Sanae M-J, et al. Infective endocarditis by HACEK: a review. Journal of Cardiothoracic Surgery. 2022;17(1):185.
17. Tubiana S, Blotière P-O, Hoen B, Lesclous P, Millot S, Rudant J, et al. Dental procedures, antibiotic prophylaxis, and endocarditis among people with prosthetic heart valves: nationwide population based cohort and a case crossover study. BMJ. 2017;358:j3776.
18. Santinga JT, Fekety RF, Bottomley WK, Else B, Willis PW. Antibiotic prophylaxis for endocarditis in patients with a prosthetic heart valve. The Journal of the American Dental Association. 1976;93(5):1001-5.
19. Chang YS, Chang CC, Chen YH, Chen WS, Chen JH. Risk of infective endocarditis in patients with systemic lupus erythematosus in Taiwan: a nationwide population-based study. Lupus. 2017;26(11):1149-56.

For peer review only

20. Russo G, Corso LD, Biasiolo A, Berengo M, Pengo V. Simple and safe method to prepare patients with prosthetic heart valves for surgical dental procedures. Clin Appl Thromb Hemost. 2000;6(2):90-3.
21. Tzukert AA, Leviner E, Sela M. Prevention of infective endocarditis: Not by antibiotics alone: A 7-year follow-up of 90 dental patients. Oral Surgery, Oral Medicine, Oral Pathology. 1986;62(4):385-8.
22. Findler M, Chackartchi T, Regev E. Dental implants in patients at high risk for infective endocarditis: a preliminary study. Int J Oral Maxillofac Surg. 2014;43(10):1282-5.
23. Littner MM, Kaffe I, Tamse A, Buchner A. New concept in chemoprophylaxis of bacterial endocarditis resulting from dental treatment. Oral Surgery, Oral Medicine, Oral Pathology. 1986;61(4):338-42.
24. Talha KM, Baddour LM, Thornhill MH, Arshad V, Tariq W, Tleyjeh IM, et al. Escalating incidence of infective endocarditis in Europe in the 21st century. Open Heart. 2021;8(2):e001846.
25. Tong SY, Davis JS, Eichenberger E, Holland TL, Fowler VG, Jr. Staphylococcus aureus infections: epidemiology, pathophysiology, clinical manifestations, and management. Clin Microbiol Rev. 2015;28(3):603-61.
26. Remadi JP, Habib G, Nadji G, Brahim A, Thuny F, Casalta JP, et al. Predictors of death and impact of surgery in Staphylococcus aureus infective endocarditis. Ann Thorac Surg. 2007;83(4):1295-302.
27. Fukushima S, Hagiya H, Mizuta Y, Nakano Y, Takahara M, Okamoto K, et al. Multiple Deep-seated Dentofacial Abscesses Caused by Multidrug-resistant Viridans Group Streptococci. Intern Med. 2020;59(16):2067-70.
28. Smith A, Jackson MS, Kennedy H. Antimicrobial susceptibility of viridans group streptococcal blood isolates to eight antimicrobial agents. Scand J Infect Dis. 2004;36(4):259-63.
29. Tuohy M, Washington JA. Antimicrobial susceptibility of viridans group streptococci. Diagn Microbiol Infect Dis. 1997;29(4):277-80.
30. Thornhill MH, Gibson TB, Cutler E, Dayer MJ, Chu VH, Lockhart PB, et al. Antibiotic Prophylaxis and Incidence of Endocarditis Before and After the 2007 AHA Recommendations. Journal of the American College of Cardiology. 2018;72(20):2443-54.
31. Edwards C, Lacey A, Anderson M, Bodger O. 98 Are we seeing more cases of Infective Endocarditis after Nice CG64 Recommended Cessation of the use of Antibiotic Prophylaxis? The Welsh Experience. Heart. 2015;101(Suppl 4):A56-A7.
32. Thornhill MH, Gibson TB, Yoon F, Dayer MJ, Prendergast BD, Lockhart PB, et al. Antibiotic Prophylaxis Against Infective Endocarditis Before Invasive Dental Procedures. J Am Coll Cardiol. 2022;80(11):1029-41.
33. Thornhill MH, Gibson TB, Yoon F, Dayer MJ, Prendergast BD, Lockhart PB, et al. Endocarditis, invasive dental procedures, and antibiotic prophylaxis efficacy in US Medicaid patients. Oral Dis. 2023.
34. Thornhill MH, Crum A, Campbell R, Stone T, Lee EC, Bradburn M, et al. Temporal association between invasive procedures and infective endocarditis. Heart. 2023;109(3):223-31.

For peer review only

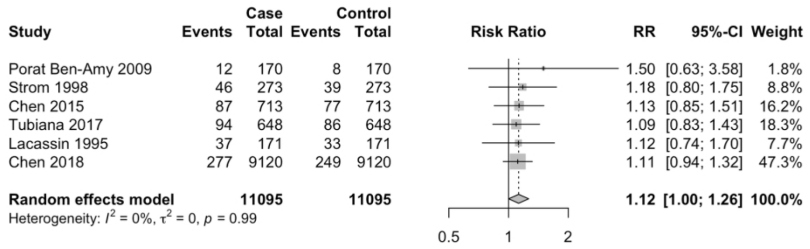

Figure 1. Proportion of dental procedures in case vs control in case-control or crossover studies

722x538mm (72 x 72 DPI)

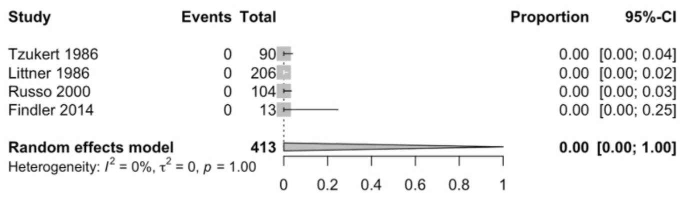

Figure 2. Proportion of IE in high-risk patients who underwent dental procedures

722x538mm (72 x 72 DPI)

Supplementary Materials

Supplementary Table 1: Search strategy by databases

Database	Search term
Medline	("dent*"[All Fields] OR "dontic*"[All Fields]) AND "endocarditis"[All Fields]
Embase	((“dent*” or “*dontic*”) and “endocarditis”).mp.
Scopus	TITLE-ABS-KEY (("dent*" OR "dontic") AND "endocarditis")
Clinicaltrials.gov	Endocarditis

For peer review only

Supplementary Table 2: Quality assessment of included studies using the Newcastle-Ottawa Scale

**Author, Year, Country**	**Selection**				**Comparability**	**Outcome**			**Total quality score**
	Cohort studies: Representativeness of the exposed cohort	Selection of non-exposed cohort	Ascertainment of exposure	Demonstration that the current outcome of interest was not present at the start of study	Comparability of the cohorts on the basis of design or analysis	Ascertainment of outcome	Was follow up long enough for outcomes to occur	Adequacy of follow up of cohorts	
	Case control studies: Case definition adequate	Representativeness of cases	Selection of controls	Definition of controls	Comparability of cases and controls on the basis of design or analysis	Ascertainment of exposure	Same method of ascertainment for cases and controls	Non-response rate	
Aziz et al., 2010, US	★	★		★		★			4
Ballesta et al., 2022, Spain	★	★	★	★	★★	★	★		8
Carmona et al., 2002, Spain	★	★	★	★		★	★		6
Carmona et al.,	★	★	★	★	★	★	★		7

2003, Spain									
Chang et al., 2017, Taiwan	★	★	★	★	★★	★	★	★	8
Chen et al., 2015, Taiwan	★	★	★	★	★	★	★	★	8
Chen et al., 2018, Taiwan	★	★	★	★	★★	★	★	★	9
Chirillo et al., 2016, Italy	★	★	★	★		★	★	★	7
Chu et al., 2004, New Zealand	★	★		★		★	★		5
Cukingnan et al., 1983, US	★	★	★	★		★	★	★	7
Delahaye et al., 2016, France	★	★	★	★		★	★	★	7
Dominguez et al., 2016, Spain	★	★	★	★	★★	★	★		8
Durack et al., 1983, US				★			★		2
Duval et al., 2006, France	★	★	★	★	★	★	★	★	8
Duval et al., 2017, France	★	★	★	★	★★		★	★	8

For peer review only

Findler et al., 2014, Israel	★	★	★	★		★	★		7
Grover et al., 1991, India	★	★	★	★		★	★	★	7
Hricak et al., 2013, Slovakia	★	★	★	★		★	★	★	7
Imperiale et al., 1990, US	★	★	★	★	★★	★	★		8
Kim et al., 2019, Korea	★	★	★	★		★	★	★	7
Krcmery et al., 2018, Slovakia	★	★	★	★		★	★	★	7
Lacassin et al., 1995, France	★	★		★	★★		★		6
Littner et al., 1986, Israel	★			★		★	★		4
Loupa et al., 2004, Greece	★	★	★	★		★	★		6
Luk et al., 2014, Canada	★	★	★	★		★	★		6
Martin et al., 2007, UK	★		★	★		★	★		5
Mudhumitha et al., 2018, India	★	★	★	★		★	★	★	7
Porat Ben-Amy et al.,	★	★	★	★	★★	★	★		8

For peer review only

2009, Israel									
Russo et al., 2000, Italy	★	★		★			★		4
Santinga et al., 1976, US			★	★			★		3
Sett et al., 1993, Canada	★	★	★	★	★★	★	★	★	9
Siegman-Igra et al., 2010, Israel	★	★	★	★		★	★		6
Smith et al., 1976, UK	★			★			★		3
Strom et al., 1998, US	★	★	★	★	★★	★	★		8
Takeda et al., 2005, Japan	★	★	★	★		★	★		6
Tubiana et al., 2017, France	★	★	★	★	★★	★	★	★	9
Tzukert et al., 1986, Israel				★			★		2
Weinberger et al., 1990, Israel	★	★	★	★		★	★		6

For peer review only

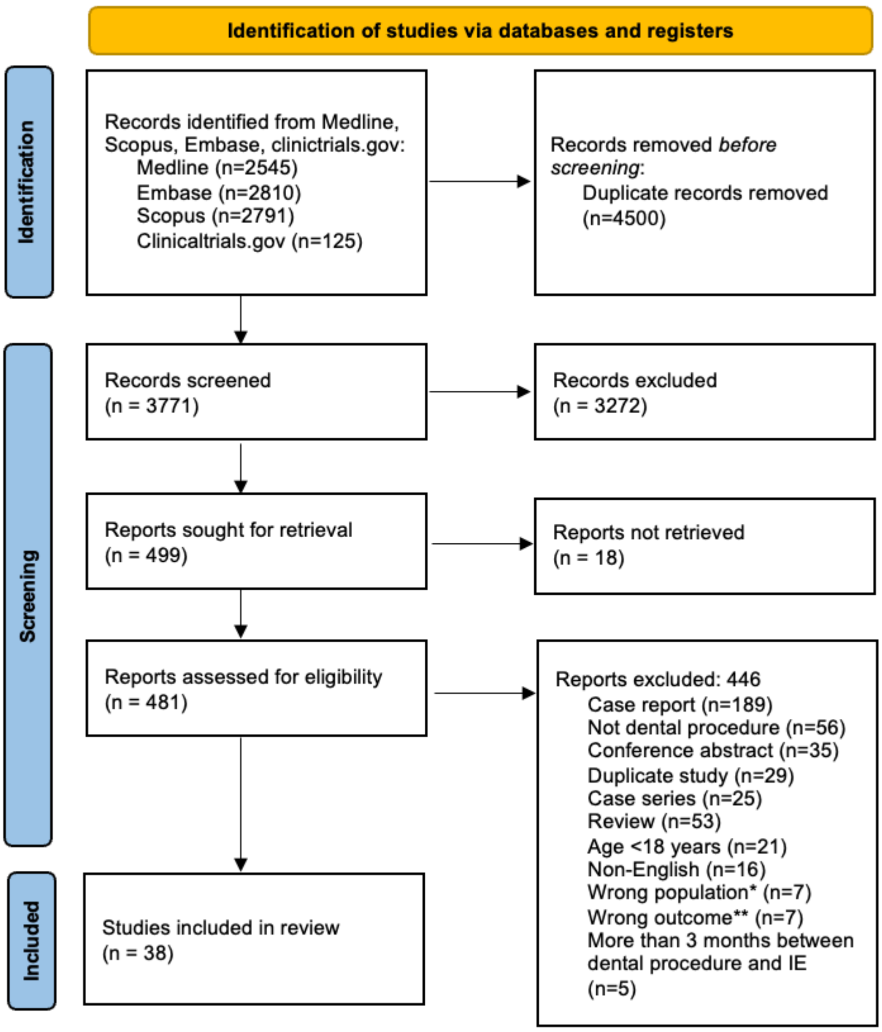

*"Wrong population" refers to studies that did not include patients with IE and dental procedures

**"wrong outcomes" refers to studies that did not report findings relevant to the conclusions of the present systematic review

Supplementary Figure 1. PRISMA flow diagram of the study selection process

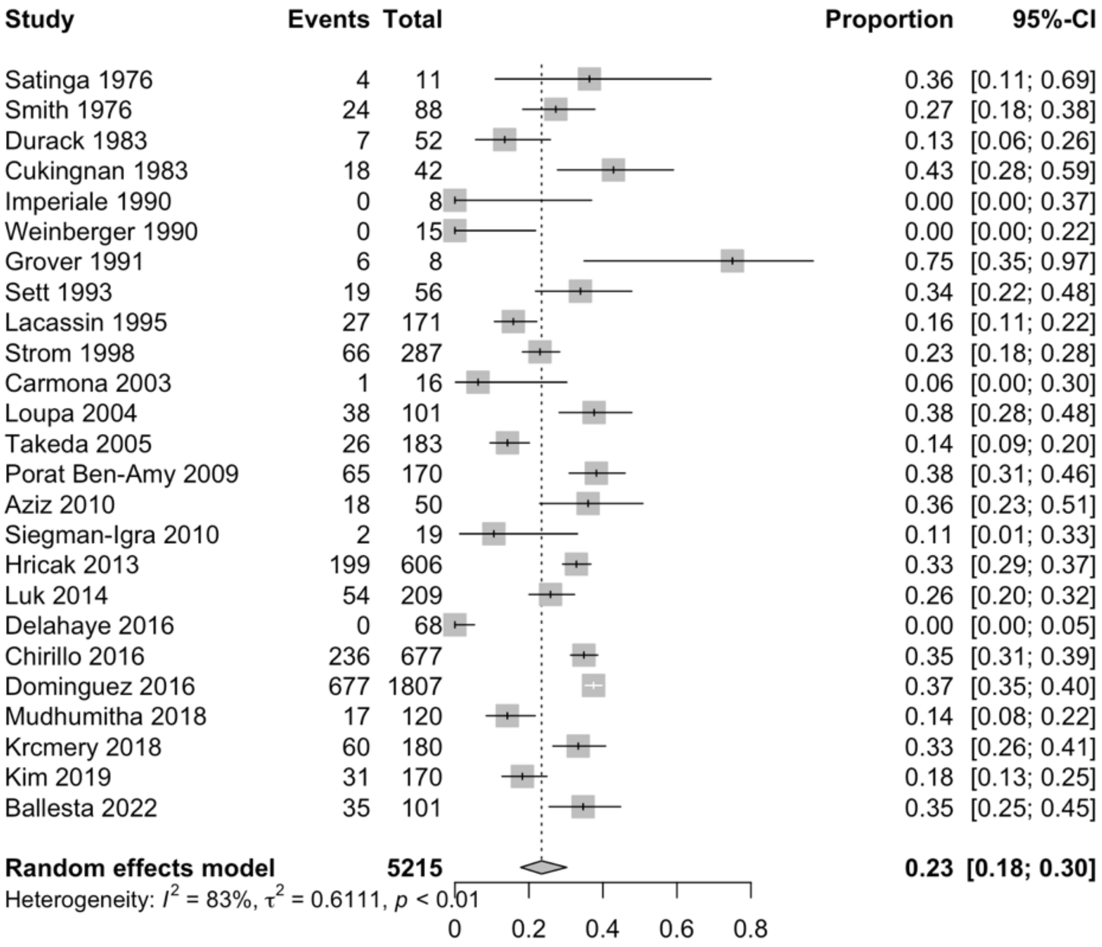

Supplementary Figure 2A. Proportion of *Staphylococcus* bacteraemia

iew only

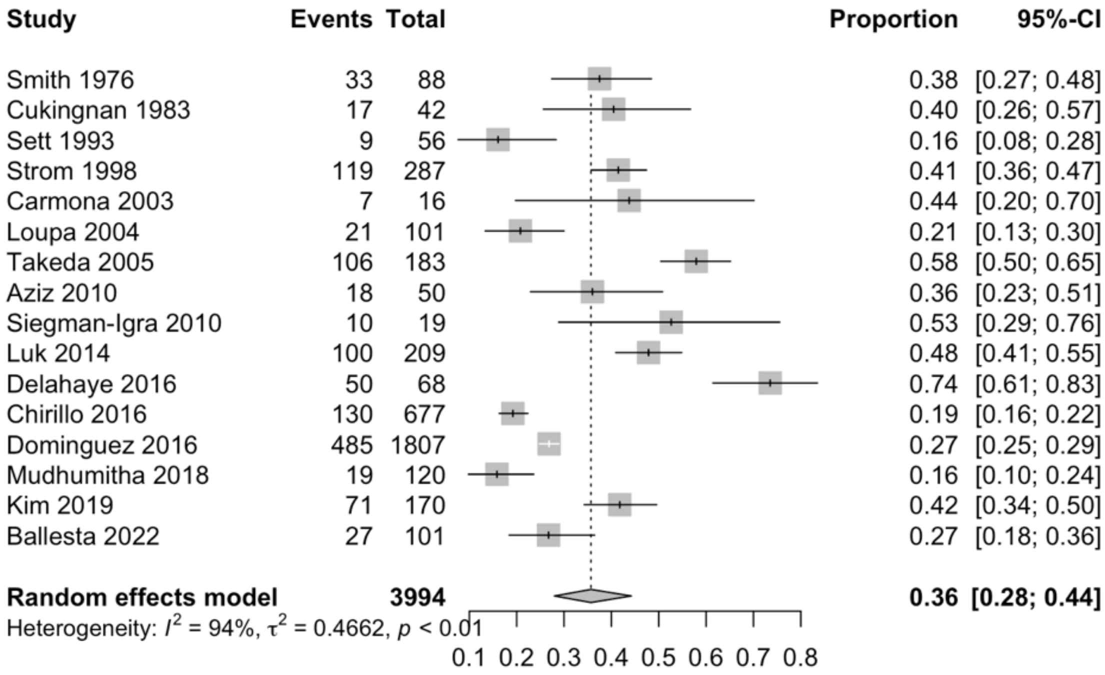

Supplementary Figure 2B. Proportion of *Streptococcus* bacteraemia

For peer review only

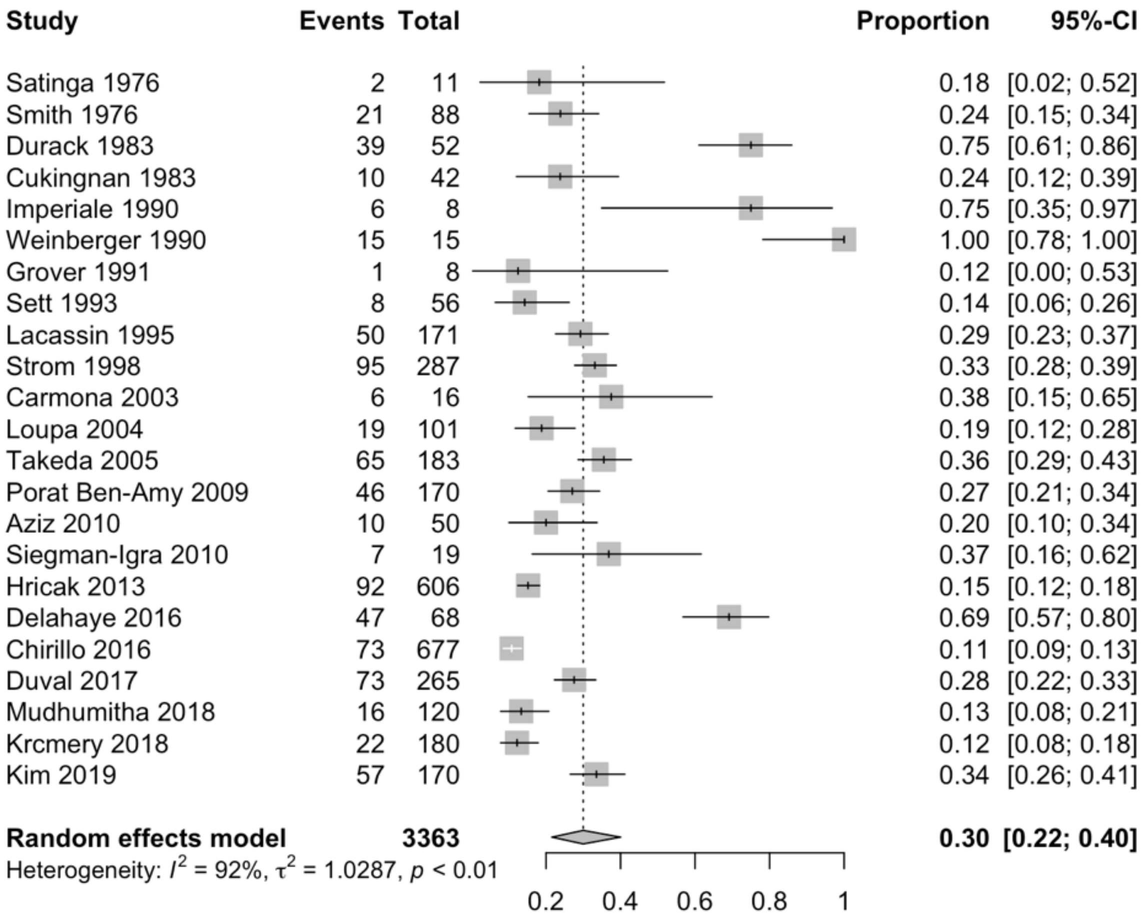

Supplementary Figure 2C. Proportion of *Streptococcus viridans* bacteraemia

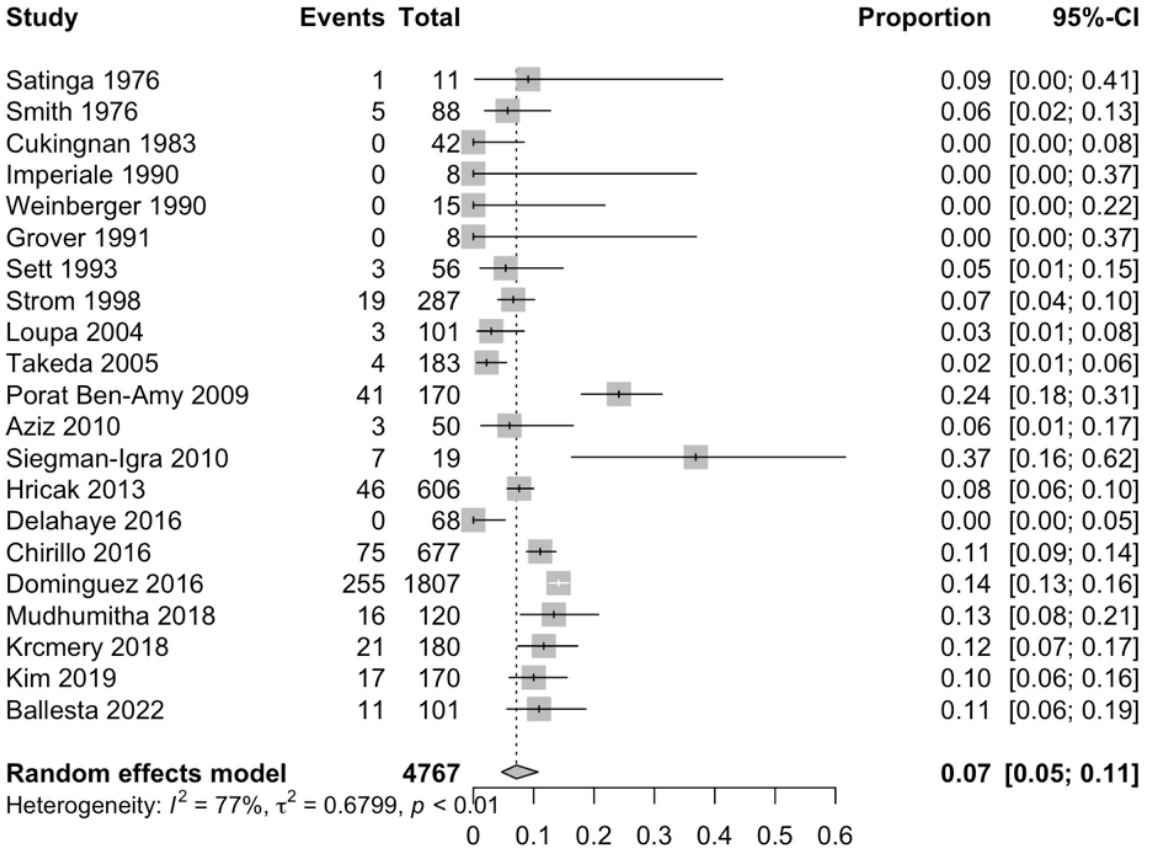

Supplementary Figure 2D. Proportion of *Enterococcus* bacteraemia

eview only

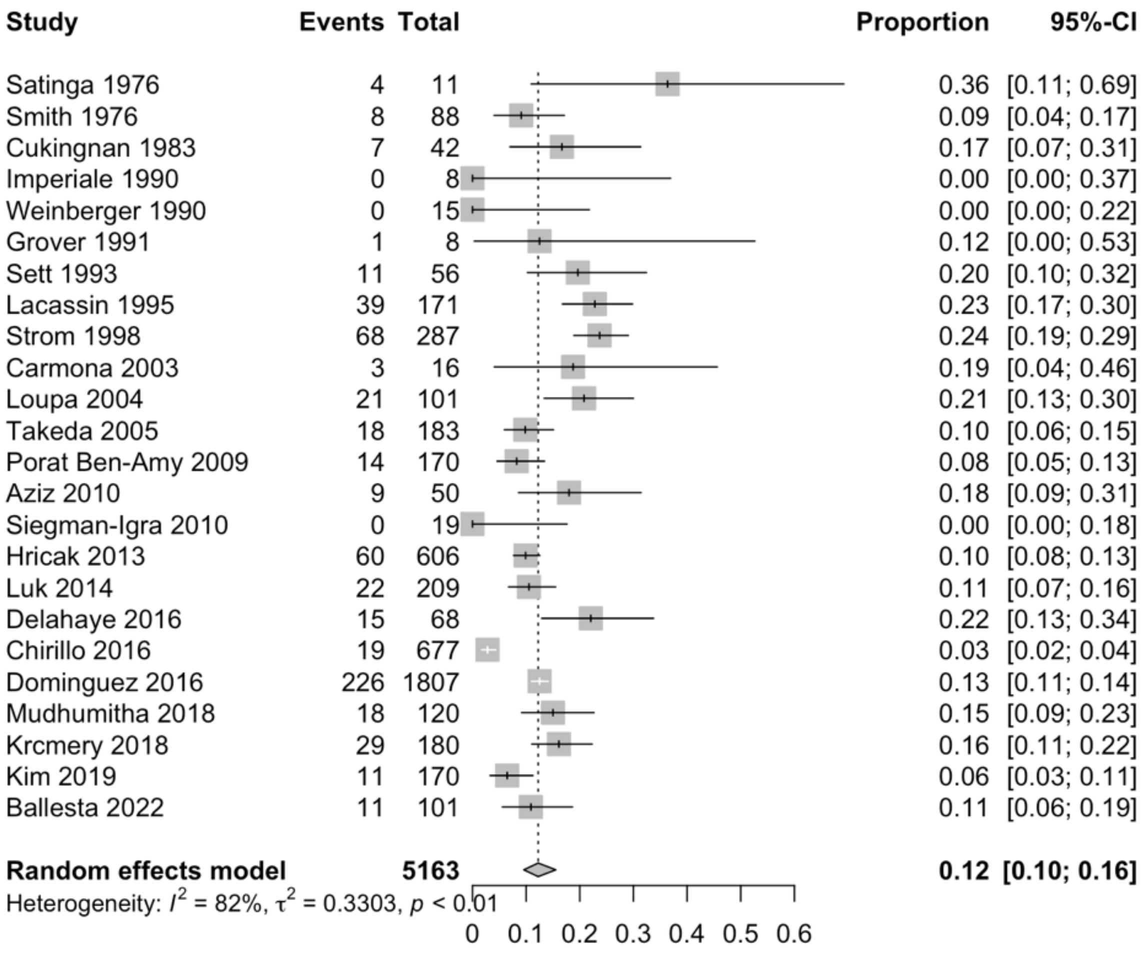

Supplementary Figure 2E. Proportion of HACEK and others bacteraemia

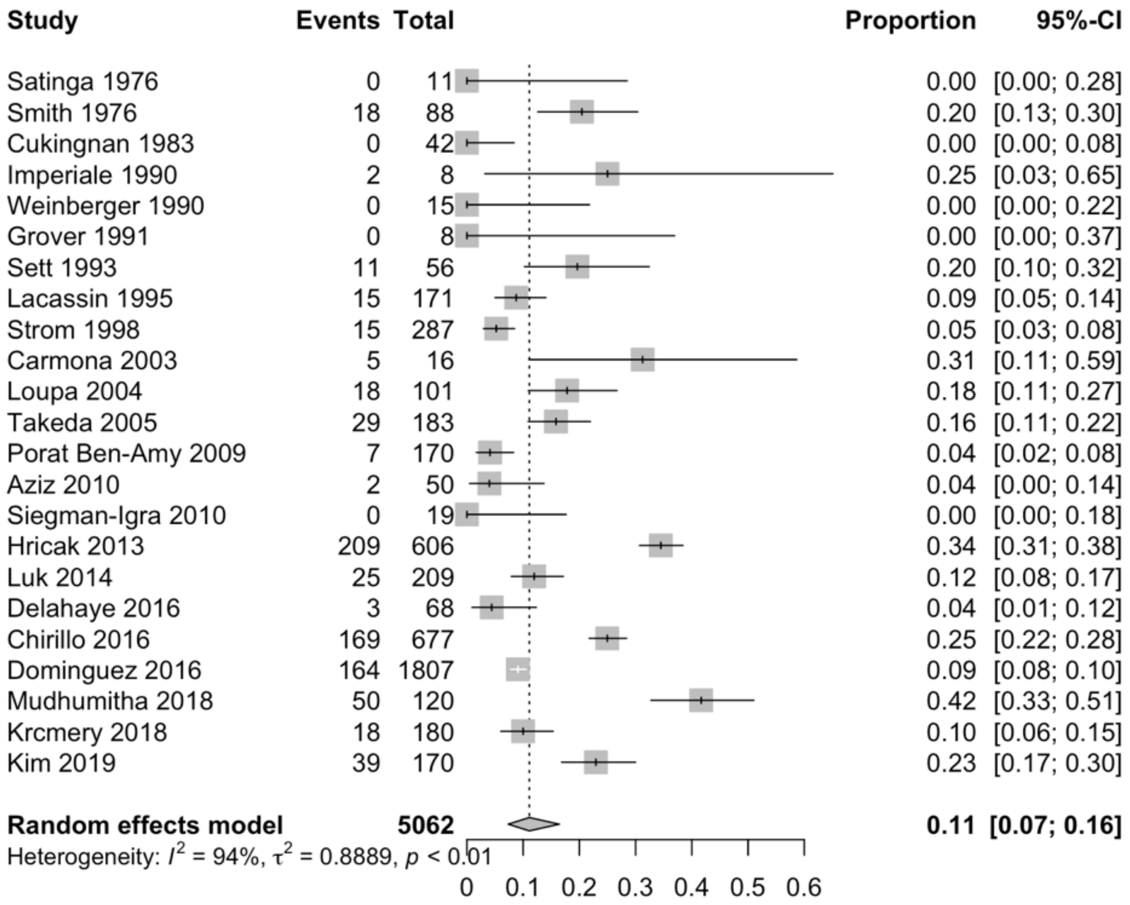

Supplementary Figure 2F. Proportion of culture negative infective endocarditis

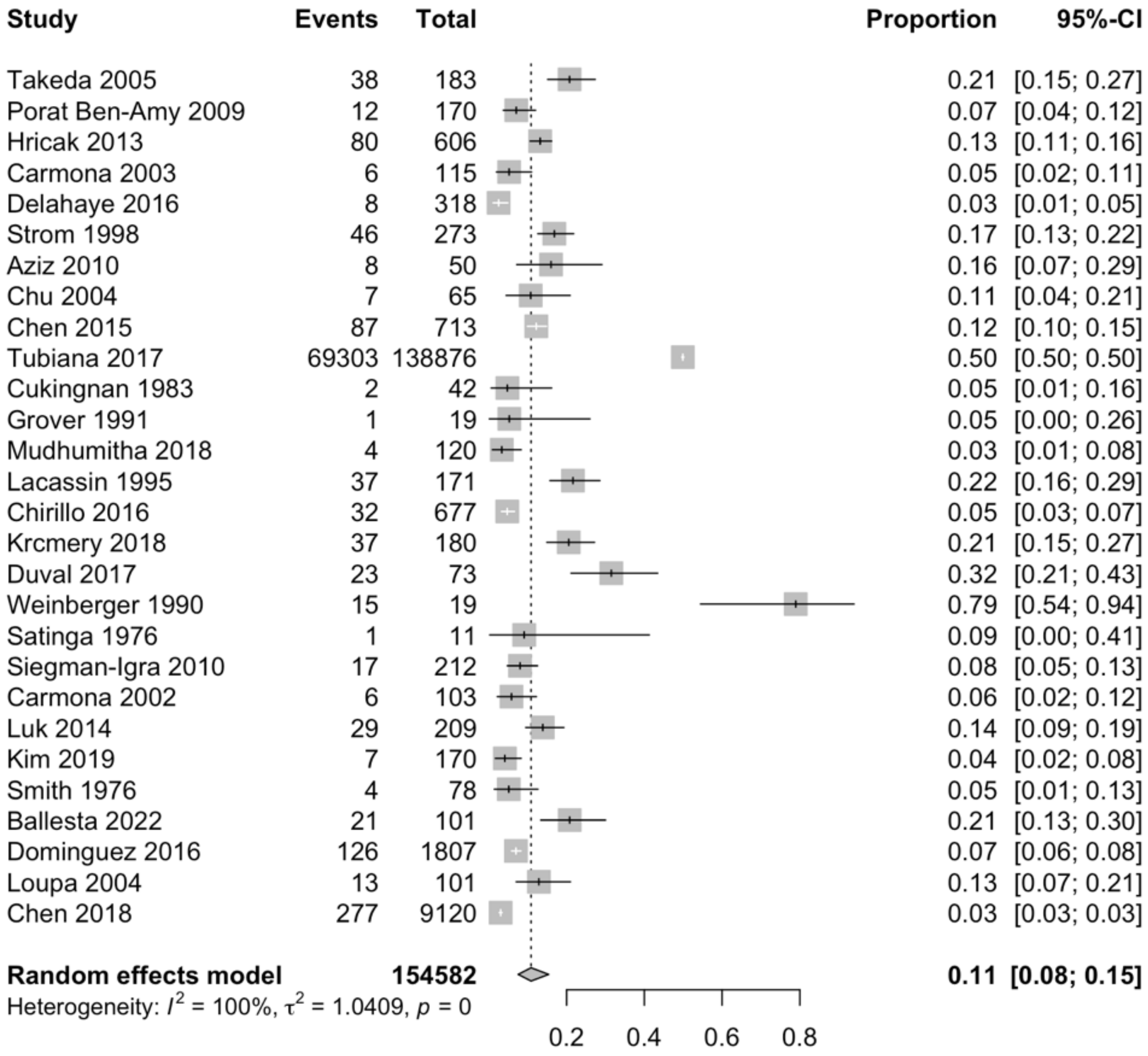

Supplementary Figure 3. Proportion of dental procedures in IE patients

For peer review only

PRISMA 2020 Checklist

Section and Topic	Item #	Checklist item	Location where item is reported
**TITLE**			
Title	1	Identify the report as a systematic review.	Yes
**ABSTRACT**			
Abstract	2	See the PRISMA 2020 for Abstracts checklist.	Yes
**INTRODUCTION**			
Rationale	3	Describe the rationale for the review in the context of existing knowledge.	Yes
Objectives	4	Provide an explicit statement of the objective(s) or question(s) the review addresses.	Yes
**METHODS**			
Eligibility criteria	5	Specify the inclusion and exclusion criteria for the review and how studies were grouped for the syntheses.	Yes
Information sources	6	Specify all databases, registers, websites, organisations, reference lists and other sources searched or consulted to identify studies. Specify the date when each source was last searched or consulted.	Yes
Search strategy	7	Present the full search strategies for all databases, registers and websites, including any filters and limits used.	Yes
Selection process	8	Specify the methods used to decide whether a study met the inclusion criteria of the review, including how many reviewers screened each record and each report retrieved, whether they worked independently, and if applicable, details of automation tools used in the process.	Yes
Data collection process	9	Specify the methods used to collect data from reports, including how many reviewers collected data from each report, whether they worked independently, any processes for obtaining or confirming data from study investigators, and if applicable, details of automation tools used in the process.	Yes
Data items	10a	List and define all outcomes for which data were sought. Specify whether all results that were compatible with each outcome domain in each study were sought (e.g. for all measures, time points, analyses), and if not, the methods used to decide which results to collect.	Yes
	10b	List and define all other variables for which data were sought (e.g. participant and intervention characteristics, funding sources). Describe any assumptions made about any missing or unclear information.	Yes
Study risk of bias assessment	11	Specify the methods used to assess risk of bias in the included studies, including details of the tool(s) used, how many reviewers assessed each study and whether they worked independently, and if applicable, details of automation tools used in the process.	Yes
Effect measures	12	Specify for each outcome the effect measure(s) (e.g. risk ratio, mean difference) used in the synthesis or presentation of results.	Yes
Synthesis methods	13a	Describe the processes used to decide which studies were eligible for each synthesis (e.g. tabulating the study intervention characteristics and comparing against the planned groups for each synthesis (item #5)).	Yes
	13b	Describe any methods required to prepare the data for presentation or synthesis, such as handling of missing summary statistics, or data conversions.	Yes
	13c	Describe any methods used to tabulate or visually display results of individual studies and syntheses.	Yes
	13d	Describe any methods used to synthesize results and provide a rationale for the choice(s). If meta-analysis was performed, describe the model(s), method(s) to identify the presence and extent of statistical heterogeneity, and software package(s) used.	Yes
	13e	Describe any methods used to explore possible causes of heterogeneity among study results (e.g. subgroup analysis, meta-regression).	Yes
	13f	Describe any sensitivity analyses conducted to assess robustness of the synthesized results.	Yes
Reporting bias assessment	14	Describe any methods used to assess risk of bias due to missing results in a synthesis (arising from reporting biases).	Yes
Certainty assessment	15	Describe any methods used to assess certainty (or confidence) in the body of evidence for an outcome.	Yes

For peer review only - http://bmjopen.bmj.com/site/about/guidelines.xhtml

PRISMA 2020 Checklist

Section and Topic	Item #	Checklist item	Location where item is reported
**RESULTS**			
Study selection	16a	Describe the results of the search and selection process, from the number of records identified in the search to the number of studies included in the review, ideally using a flow diagram.	Yes
	16b	Cite studies that might appear to meet the inclusion criteria, but which were excluded, and explain why they were excluded.	Yes
Study characteristics	17	Cite each included study and present its characteristics.	Yes
Risk of bias in studies	18	Present assessments of risk of bias for each included study.	Yes
Results of individual studies	19	For all outcomes, present, for each study: (a) summary statistics for each group (where appropriate) and (b) an effect estimate and its precision (e.g. confidence/credible interval), ideally using structured tables or plots.	Yes
Results of syntheses	20a	For each synthesis, briefly summarise the characteristics and risk of bias among contributing studies.	Yes
	20b	Present results of all statistical syntheses conducted. If meta-analysis was done, present for each the summary estimate and its precision (e.g. confidence/credible interval) and measures of statistical heterogeneity. If comparing groups, describe the direction of the effect.	Yes
	20c	Present results of all investigations of possible causes of heterogeneity among study results.	Yes
	20d	Present results of all sensitivity analyses conducted to assess the robustness of the synthesized results.	Yes
Reporting biases	21	Present assessments of risk of bias due to missing results (arising from reporting biases) for each synthesis assessed.	Yes
Certainty of evidence	22	Present assessments of certainty (or confidence) in the body of evidence for each outcome assessed.	Yes
**DISCUSSION**			
Discussion	23a	Provide a general interpretation of the results in the context of other evidence.	Yes
	23b	Discuss any limitations of the evidence included in the review.	Yes
	23c	Discuss any limitations of the review processes used.	Yes
	23d	Discuss implications of the results for practice, policy, and future research.	Yes
**OTHER INFORMATION**			
Registration and protocol	24a	Provide registration information for the review, including register name and registration number, or state that the review was not registered.	Yes,Prospero
	24b	Indicate where the review protocol can be accessed, or state that a protocol was not prepared.	Yes
	24c	Describe and explain any amendments to information provided at registration or in the protocol.	Yes
Support	25	Describe sources of financial or non-financial support for the review, and the role of the funders or sponsors in the review.	Yes
Competing interests	26	Declare any competing interests of review authors.	Yes
Availability of data, code and other materials	27	Report which of the following are publicly available and where they can be found: template data collection forms; data extracted from included studies; data used for all analyses; analytic code; any other materials used in the review.	Yes

From: Page MJ, McKenzie JE, Bossuyt PM, Boutron I, Hoffmann TC, Mulrow CD, et al. The PRISMA 2020 statement: an updated guideline for reporting systematic reviews. BMJ 2021;372:n71. doi: 10.1136/bmj.n71

For peer review only - http://bmjopen.bmj.com/site/about/guidelines.xhtml

For more information, visit: http://www.prisma-statement.org/

BMJ Open

Prophylactic antibiotic use for infective endocarditis - a systematic review and meta-analysis

Journal:	*BMJ Open*
Manuscript ID	bmjopen-2023-077026.R1
Article Type:	Original research
Date Submitted by the Author:	07-Aug-2023
Complete List of Authors:	Lean, Sue SH; Wei Gong Memorial Hospital<br>Jou, Eric; University of Cambridge<br>Ho, Jamie; National University Health System<br>Jou, Ernest GL; Wei Gong Memorial Hospital
**Primary Subject Heading**:	Cardiovascular medicine
Secondary Subject Heading:	Dentistry and oral medicine, Infectious diseases
Keywords:	INFECTIOUS DISEASES, Valvular heart disease < CARDIOLOGY, Cardiology < INTERNAL MEDICINE, IMMUNOLOGY

SCHOLARONE™
Manuscripts

I, the Submitting Author has the right to grant and does grant on behalf of all authors of the Work (as defined in the below author licence), an exclusive licence and/or a non-exclusive licence for contributions from authors who are: i) UK Crown employees; ii) where BMJ has agreed a CC-BY licence shall apply, and/or iii) in accordance with the terms applicable for US Federal Government officers or employees acting as part of their official duties; on a worldwide, perpetual, irrevocable, royalty-free basis to BMJ Publishing Group Ltd ("BMJ") its licensees and where the relevant Journal is co-owned by BMJ to the co-owners of the Journal, to publish the Work in this journal and any other BMJ products and to exploit all rights, as set out in our licence.

The Submitting Author accepts and understands that any supply made under these terms is made by BMJ to the Submitting Author unless you are acting as an employee on behalf of your employer or a postgraduate student of an affiliated institution which is paying any applicable article publishing charge ("APC") for Open Access articles. Where the Submitting Author wishes to make the Work available on an Open Access basis (and intends to pay the relevant APC), the terms of reuse of such Open Access shall be governed by a Creative Commons licence – details of these licences and which Creative Commons licence will apply to this Work are set out in our licence referred to above.

Other than as permitted in any relevant BMJ Author's Self Archiving Policies, I confirm this Work has not been accepted for publication elsewhere, is not being considered for publication elsewhere and does not duplicate material already published. I confirm all authors consent to publication of this Work and authorise the granting of this licence.

For peer review only

Prophylactic antibiotic use for infective endocarditis – a systematic review and meta-analysis

Authors: Sue SH Lean[*,1], Eric Jou[*,2], Jamie SY Ho[†,3], Ernest GL Jou[†,1]

[*] Both authors contributed equally to this manuscript as co-first authors

[†] Both authors contributed equally to this manuscript as co-senior authors

[1] Wei Gong Memorial Hospital, Taiwan

[2] Addenbrooke's Hospital, University of Cambridge, United Kingdom

[3] Alexandra Hospital, National University Health System, Singapore

Corresponding author

Dr Eric Jou, MB Bchir (Dist.), MA (Cantab), PhD

Email: ej290@cam.ac.uk

Address: Queens' College Cambridge, CB3 9ET, United Kingdom

For peer review only

Abstract

Objectives Infective endocarditis (IE) is a devastating disease with a 50% one-year mortality rate. In recent years, medical authorities across the globe advised stricter criteria for antibiotic prophylaxis in patients with high risk of IE undergoing dental procedures. Whether such recommendations may increase the risk of IE in at-risk patients must be investigated.

Design Prospectively registered systematic review and meta-analysis (CRD42022326664).

Data sources Medline, Embase, Scopus and Clinictrials.gov were searched through 23rd May 2022, together with an updated search on the 5th August 2023.

Eligibility criteria All primary studies reporting infective endocarditis within three months of dental procedures in adults > 18 years of age were included, while conference abstracts, reviews, case reports and case series involving fewer than 10 cases were excluded.

Data extraction and synthesis All studies were assessed by two reviewers independently, and any discrepancies were further resolved through a third researcher.

Results Of the 3771 articles screened, 38 observational studies fit the inclusion criteria and were included in the study for subsequent analysis. Overall, 11% (95% CI 0.08-0.16, I^2=100%) of IE are associated with recent dental procedures. *Streptococcus viridans* accounted for 69% (95% CI 0.46-0.85) of IE in patients who had undergone recent dental procedures, compared to only 21% (95% CI 0.17-0.26) in controls (P=0.003). None of the high risk patients developed IE across all studies where 100% of the patients were treated with prophylactic antibiotics, and IE patients are 12% more likely to have undergone recent dental manipulation compared to matched controls (95% CI 1.00-1.26, P=0.048).

Conclusions Although there is a lack of randomised control trials due to logistic difficulties in the literature on this topic, antibiotic prophylaxis are likely of benefit in reducing the

For peer review only

incidence of IE in high-risk patients after dental procedures. Further well-designed high-quality case-controlled studies are required.

Key Words: Infective endocarditis, dental, antibiotic prophylaxis, bacterial endocarditis

Strengths and limitations of this study

- A comprehensive systematic review and meta-analysis interrogating the complex question of the association between dental procedures, infective endocarditis and antibiotic prophylaxis through multiple angles
- The studies included in the present analysis originate from broad geographical locations spanning four continents including North America, Europe, Asia and Australia allowing accurate global representation
- No randomised-controlled trials were included in the present systematic review and meta-analysis due to the lack of such study type in the literature on this important topic

For peer review only

For peer review only - http://bmjopen.bmj.com/site/about/guidelines.xhtml

Introduction

Infective endocarditis (IE) is an infection of the inner linings or valves of the heart with a near 100% mortality rate before the advent of antibiotics (1). Mortality remains high despite modern advances in medicine, estimated to be around 25% during in-hospital admissions and almost 50% at one year follow-up (2). Symptoms of IE include pyrexia, chills, rigours, headaches, dyspnoea, cough, myalgia, arthralgia and general fatigue, and in addition to antibiotics up to 50% of patients may require surgical treatment (3).

Mechanistically, IE occurs due to injury to the heart lining or valve, allowing pathogens transiently travelling in the blood to lodge. Patients at elevated risk of IE include those with previous or active heart diseases which increases the susceptibility of pathogen colonisation on injured heart tissue, in accordance with the American Heart Association (AHA) and European Society of Cardiology (ESC) guidelines (Supplementary Table 1) (4-7). Importantly, dental procedures are associated with bacteraemia (8, 9), and antibiotic prophylaxis have long been used for dental procedures across the world in at-risk patients to reduce IE incidence. Nevertheless, there has been uncertainties on the efficacy of antibiotic prophylaxis (10), and in 2008 the United Kingdom (UK) National Institute for Health and Care Excellence (NICE) clinical guidelines were updated advising against their routine use in the prevention of IE in patients undergoing dental procedures. This is in contrast to most countries globally including the United States (US) and many European countries where antibiotic prophylaxis remains in-use, although there is a tendency towards stricter criteria over the years (7, 11). The updated 2007 AHA and 2009 ESC guidelines indicated that antibiotic prophylaxis is no longer recommended for moderate risk patients, and instead only for high risk patients (6, 7). On one hand, withholding antibiotic prophylaxis where there are

For peer review only

For peer review only - http://bmjopen.bmj.com/site/about/guidelines.xhtml

no foreseeable benefits is cost effective and can prevent overtreatment and side-effects. However, given that IE carries significant morbidity and mortality, it remains vital to ascertain whether measures to reduce or completely withhold antibiotic prophylaxis may lead to a higher incidence of IE secondary to dental procedures.

Despite the long history of antibiotic prophylaxis in IE prevention, there remains to be no clear evidence to support or refute their use due to the lack of high-quality study or trials (10, 12, 13). This is further complicated by most studies utilising bacteraemia rather than IE as an endpoint as highlighted in recent systematic reviews (14). In this article, we perform a systematic review and meta-analysis of the literature exploring the aetiology and underlying microbial causes of dental procedure-related IE, followed by assessing the association between IE incidence and dental manipulation. Focus is placed on analysing the prevalence of recent preceding dental manipulation in patients diagnosed with IE in relation to prophylactic antibiotic use. Furthermore, studies reporting IE incidence and outcome in high risk patients whom had undergone recent dental procedures are also interrogated. Finally, based on our findings we comment on the rationale and efficacy of prophylactic antibiotic use in high-risk patients in the prevention of IE.

Materials and Methods

The systematic review and meta-analysis protocol was prospectively registered on PROSPERO (CRD42022326664). Patients or the public were not involved in the design, or conduct, or reporting, or dissemination plans of our research. The main clinical question interrogated in this systematic review and meta-analysis is to assess the potential association

For peer review only - http://bmjopen.bmj.com/site/about/guidelines.xhtml

of dental procedures and infective endocarditis, and secondarily whether prophylactic antibiotic use may be of benefit in IE prevention relating to dental procedures. Due to the lack of randomised-controlled trials in the literature, we attempted to answer this pressing question through utilising a multifaceted approach.: Firstly, we assessed the potential association between IE and recent dental procedures through the inclusion of studies where incidence of recent dental procedures were reported in IE patients. Additionally, assessments comparing the causative organisms of IE associated with recent dental procedures to general IE cases were also performed in studies where such data is reported. Furthermore, studies where patients at risk of IE were treated with prophylactic antibiotics for dental procedures were also interrogated to assess for any association between antibiotic prophylaxis and IE prevention.

Study selection

A systematic search of four databases namely Medline, Embase, Scopus and Clinictrials.gov was performed from the date of inception to 23 May 2022, with the search terms “dent*” OR “*dontic*” AND “endocarditis” (Supplementary Table 2). References of included studies and relevant reviews were hand searched for additional articles. Inclusion criteria were primary studies involving adults, reporting infective endocarditis within three months of dental

For peer review only

procedures. The full inclusion criteria are shown in Supplementary Table 3. Randomized controlled trials, cohort studies, case-control studies, cross-sectional studies and case series with 10 or more cases were included. Studies that reported dental sources, but not specifically dental procedures were excluded. Conference abstracts, reviews, case reports and case series involving fewer than 10 cases and non-English studies were excluded. Title and abstracts were screened by two reviewers independently, and full texts were subsequently reviewed for included studies and those with discrepancies. Conflicts were resolved by discussion and involvement of a third researcher. An updated search was performed on the 5th of August 2023 using the same search strategy, of which a total of four studies satisfied the eligibility criteria (15-18). The four studies identified in the updated search is discussed in the discussion section and the findings compared to the meta-analysis from the original search.

Data extraction

Data were extracted onto a standardized form by two researchers independently, which included the country of publication, study design, population, type of dental procedure, proportion of infective endocarditis, organism identified on blood culture, use of prophylactic antibiotics and outcomes.

Exposure and Outcomes

In this study, we first investigated the causative organisms for infective endocarditis and compared this between patients who had recent dental procedures to those who did not. Infective endocarditis was diagnosed according to the Duke criteria. Dental procedures included tooth extraction, fillings, scaling, endodontal and periodontal treatment, implant

For peer review only

insertion and root canal treatment. Causative organisms were identified by microbiology investigations. We then analysed the proportion of patients with IE who underwent dental procedures within 3 months prior to the diagnosis of IE. The proportion of patients with exposure to recent dental procedures were compared between patients with IE and those without IE. To understand the risk of IE post-dental procedure, the proportion of high-risk patients who developed IE within 3-months post-dental procedure was analysed. High-risk patients were defined based on the AHA/ESC guidelines (Supplementary Table 1).

Quality assessment

The quality of the studies included in the systematic review was assessed using the Newcastle Ottawa Scale for cohort studies, case-control studies and case series.

Statistical analysis

Statistical tests were performed using the *meta* and *metafor* packages on R (R Core Team (2021)), based on the guidelines outlined in the Cochrane Handbook. Meta-analysis of IE and dental procedure proportions was performed using the random-effects inverse variance model with logit transformation and DerSimonian and Laird method, and subgroup analysis was performed based on the region of publication, year of publication and proportion of patients with antibiotic prophylaxis. The type of bacterial organisms isolated from blood cultures were also analysed by meta-analysis of proportions as detailed above. Random-effects meta-analysis of binary outcomes was performed for case-control studies using the inverse variance model, where proportion of IE cases with exposure to dental procedures was compared to controls without IE. Sensitivity analysis using the random-effects generalised linear mixed models was also performed. Between-study heterogeneity was presented using the I^2 statistic. Publication bias was assessed by funnel plot and Egger's test for funnel plot

For peer review only - http://bmjopen.bmj.com/site/about/guidelines.xhtml

asymmetry for analysis with more than 10 studies. A p value of <0.05 was considered statistically significant.

Results

A total of 3771 articles were identified after removal of duplicates, 3272 articles were excluded at the title and abstract screening stage as they were not relevant to the topic, and a final total of 38 articles satisfied the inclusion criteria and were included in this review (Supplemental Figure 1). The risk of bias assessment is shown in Supplementary Table 4.

Infective endocarditis aetiology and causative organisms

The development of infective endocarditis follows a two-step process whereby initial damage to the endocardium provides a nidus allowing pathogen settlement and colonisation following a period of transient bacteraemia or fungemia. Studies indicate that bacteria are the most common pathogen class responsible for IE (19, 20). Common oral commensals or pathogens that cause infective endocarditis include *Streptococcus viridans* (21) and those of the HACEK (*Haemophilus*, *Aggregatibacter*, *Cardiobacterium*, *Eikenella*, and *Kingella*) group (22).

In this systematic review, 26 studies reported the microbial species responsible for the IE cases (Table 1). Pooled analysis revealed that *Staphylococcus* species accounted for 27% (95% CI 0.23 to 0.31, $I^2 = 85\%$) of IE cases overall (Supplementary Figure 2A), while *Streptococcus* species accounted for 36% (95% CI 0.29 to 0.44, $I^2 = 94\%$) (Supplementary Figure 2B). *Streptococcus viridans* and *Enterococcus* are responsible for 28% (95% CI 0.22

For peer review only - http://bmjopen.bmj.com/site/about/guidelines.xhtml

to 0.36, I^2 = 92%) (Supplementary Figure 2C) and 9% (95% CI 0.07 to 0.12, I^2 = 79%) (Supplementary Figure 2D) of total IE cases respectively. 13% (95% CI 0.10 to 0.16, I^2 = 83%) of all IE cases are due to HACEK and other organisms (Supplementary Figure 2E), while in another 13% (95% CI 0.09 to 0.18, I^2 = 94%) (Supplementary Figure 2F) the causative organism could not be identified.

20 of the 26 studies presented pooled data from all IE patients regardless of cause, while the other six reported exclusively the causative microorganism in IE patients who received recent dental procedures (Table 1). The majority of the 20 studies with pooled data (12 out of 20; 60%) found *Staphylococcus* to be the most common cause of IE, ranging from 14% to 75% of patients. This is closely followed by *Streptococcus* species collectively being the dominant cause in seven out of the 20 pooled studies (35%), ranging from 27.5% to 57%. Of the *Streptococcus* species found in these studies, *Streptococcus viridans* are the most common and are likely associated with the subgroup that received prior dental treatment. Other *Streptococcus* species identified include *Streptococcus bovis* and *Streptococcus pneumonia* which account for the remaining cases. Of the six studies where the causative organisms of IE were portrayed specifically in patients whom received prior dental procedures, *Streptococcus* species were the most common (Table 1). *Streptococcus viridans* accounted for the majority, ranging from 23.9% and up to 100% of the total IE cases. HACEK and other organisms tend to be the next most common, while *Staphylococcus* is rare unlike in the general population. Subgroup meta-analysis comparing studies reporting microbial causes of IE exclusively in patients who had undergone recent dental procedures compared to general IE cases revealed important differences in the underlying cause of IE (Table 1). In those with prior dental procedures, *Streptococcus viridans* are responsible for a significantly higher proportion at 69% (95% CI 0.46 to 0.85; I^2 = 63.7%) of cases, compared to 21% (95% CI 0.17 to 0.26; I^2 =

For peer review only - http://bmjopen.bmj.com/site/about/guidelines.xhtml

89.0%) in general IE patients (P = 0.003). Conversely, those with recent dental procedures are less likely (P = 0.002) to suffer from *Staphylococcus* IE at only 3% (95% CI 0.01 to 0.14, I^2 = 0%) compared to 29% in general IE patients (95% CI 0.25 to 0.34, I^2 = 85.9%).

Proportion of infective endocarditis cases associated with dental procedures

33 studies assessed the proportion of IE patients who had recently undergone dental procedures within three months (Table 2), with cohort sizes ranging from 11 to 138,876 patients (23, 24). The most common dental procedures reported include tooth extraction, fillings, scaling and oral surgeries, although most studies did not specify (21 out of 33, 64%). The proportion of patients whom had undergone recent dental procedures, which are considered to be the most likely cause of the IE in these patients, varied widely between studies (2.5% to 49.9%). This amounted to an overall 11% (95% CI 0.08 to 0.16, I^2 = 100%) (Supplementary Figure 3) incidence of IE patients whom had received recent dental procedures. Subgroup analysis by continent including North America, Europe, Asia and Australia revealed no differences in the proportion of IE patients with recent dental procedures (P = 0.99) (Table 1). Meta-regression for percentage of antibiotic use (intercept co-efficient -0.809; p=0.653) and year of publication (intercept co-efficient -0.005; p=0.772) revealed no significant relationship with the proportion of IE patients who underwent recent dental procedures (Supplementary Figure 4).

Proportion of patients that received dental procedures in case control studies

We further analysed the included case-control studies to assess for incidence of recent dental treatment in IE patients compared to controls without IE, which amounted to a total of six eligible studies including both conventional case-control along with case-crossover studies.

For peer review only

For peer review only - http://bmjopen.bmj.com/site/about/guidelines.xhtml

Four studies were case-crossover studies, where cases were their own controls, thus minimising differences in baseline characteristics, while the other two studies used matched control populations to reduce confounding factors. Strom et al. used age-, sex- and residence-matched controls (25), while Lacassin et al. matched the age, sex and underlying cardiac conditions between cases and controls (26). Intriguingly, whilst none of the individual studies showed a statistically significant difference in the proportion of recent dental procedures comparing IE cases to controls, pooled meta-analysis revealed that IE patients are more likely to have received recent (within three months) dental treatment compared to matched controls (Relative Risk (RR) = 1.12; 95% CI 1.00 to 1.26, $I^2 = 0\%$, $P = 0.048$) (Figure 1). These findings support the notion that recent dental procedures may be associated with higher IE incidence.

Infective endocarditis incidence in patients who underwent dental procedures

Five eligible studies investigated IE incidence in patients after receiving recent dental procedures (Table 3). All of these are cohort studies, with three being retrospective while two prospective. One retrospective study with 12,102 systemic lupus erythematosus (SLE) patients from Taiwan did not mention the proportion of patients that received prophylactic antibiotics (27). In that study, Cox multivariate proportional hazard analysis showed that recent dental procedures is an independent risk factor for IE (HR = 36.8, $P < 0.001$). The remaining four studies each reported that 100% of their patients received prophylactic antibiotics, consistent with their study design as all patients were at high risk of IE (Figure 2), except in one study by Littner et al published in 1986 which also included moderate risk patients treated with antibiotic prophylaxis as this was before the 2007 AHA and 2009 ESC guideline update (28). In an Italian prospective cohort study of 104 patients with prosthetic

For peer review only - http://bmjopen.bmj.com/site/about/guidelines.xhtml

heart valves, no patients developed IE three months after the dental surgery (29). Another prospective cohort study (Israel) involving 90 patients with prosthetic heart valve or rheumatic heart disease similarly found no cases of IE after dental procedures (scaling, fillings, extraction, root canal, crowns and bridges) (30). In that study, the striking zero IE cases observed following an optimal protocol of administering appropriate prophylactic antibiotics proven to be efficacious against *Streptococcus viridans*, is significantly lower than what would be expected statistically. This suggests that appropriate antibiotic prophylaxis may reduce IE incidence. Similarly, the remaining two retrospective cohort studies both found no cases of IE following dental procedures in high-risk patients (28, 31). In one study, 13 patients received dental implants with no cases of IE at two-year follow-up (31). In the Littner et al study where 206 patients were assessed which included both moderate risk in addition to high risk patients, none of them developed endocarditis. Of note, a minority (13.1%) developed mild side effects from prophylactic amoxicillin administration for dental procedures (extraction, abscess drainage, oral surgery, root canal, scaling, crowns and bridges) (28). In that study, the most common side effects were gastrointestinal, including abdominal discomfort (11 patients; 5.3%), nausea (6 patients; 2.9%) and constipation (5 patients; 2.4%). Overall, the severity of these side effects was classified as very mild, and patients showed a high degree of compliance to the prophylactic treatment. Collectively, these studies suggest that whilst recent dental procedures are independently associated with IE risk, prophylactic antibiotic use may be beneficial in reducing IE incidence.

Publication bias

For the outcome of proportion of IE patients who underwent recent dental procedures, the funnel plot showed asymmetry, confirmed on Egger's test (t = -4.46, p=0.0001)

For peer review only - http://bmjopen.bmj.com/site/about/guidelines.xhtml

284 (Supplementary Figure 5). The meta-analysis of the proportion of Staphylococcus and
285 Enterococcus IE showed asymmetrical funnel plots while the proportions of Streptococcus,
286 Streptococcus viridans, HACEK and culture negative IE did not show asymmetrical funnel
287 plots (Supplementary Figure 5). Therefore, there may potentially be publication bias and
288 small study effects, and further research is needed to confirm the findings of this study.

289

290 **Discussion**

291 In this systematic review and meta-analysis, we address the critical question of whether
292 current antibiotic prophylaxis regimes are effective and of benefit in reducing IE incidence in
293 high-risk patients. This is particularly crucial in an era where infective endocarditis incidence
294 continues to rise globally (32).

295

296 We found *Streptococcus* species to be the most common cause of IE followed by
297 *Staphylococcus* upon pooled analysis of 28 eligible studies with suitable data. This differs
298 from the current consensus where modern studies found *Staphylococcus* to be the leading
299 cause of IE in the general population (33). The overrepresentation of *Streptococcus* IE in our
300 systematic review is likely a reflection of the included studies typically involving dental
301 procedures. Accordingly, our meta-analysis found that microbial causes of IE is significantly
302 different in IE patients who had undergone recent dental procedures compared to general IE
303 cases, with a striking almost 70% prevalence of *Streptococcus viridans* IE in the former
304 group. Conversely, we found that patients who received prior dental manipulation are much

For peer review only

less likely to suffer from *Staphylococcus* IE at only 3% compared to 29% of the general IE patients. Other studies have similar found *Staphylococcus aureus* to account for approximately 30% of overall IE cases (34). The association of *Streptococcus viridans* with post dental procedure IE observed here is consistent with the former being a common cause of odontogenic diseases (35). These findings suggest that any prophylactic antibiotic use for dental procedures should be tailored to best protect against *Streptococcus viridans* to ensure maximal efficacy. Importantly, whilst amoxicillin is commonly used for antibiotic prophylaxis prior to dental procedures in at-risk patients, studies have shown *Streptococcus viridans* to be resistant to amoxicillin to differing degrees, and may contribute to the lack of efficacy of prophylactic antibiotics found in some studies (36, 37).

Gram negative organisms, in particular the oral commensals of the HACEK group, remain as important causes of IE despite accounting for only a small proportion of cases compared to gram positive cocci *Streptococcus* and *Staphylococcus* (22). Our systematic review showed that many studies in the literature do not report HACEK organisms as a separate categorical cause of IE to other miscellaneous pathogens. Future studies segregating HACEK organisms from other bacterial causes of IE will shed insight on whether prophylactic antibiotics use is efficacious in preventing HACEK IE specifically.

We found that segregating studies into two groups defined as above or below average use of prophylactic antibiotics for dental procedures revealed that increased prophylactic antibiotic use may reduce IE from 18% to 11.5% although this was not statistically significant (P = 0.49). Our findings cannot rule out antibiotics as important prophylactic treatment in reducing IE incidence in high-risk patients, as only a total of 11 eligible studies had suitable data for

For peer review only

analysis, and as such our meta-analysis is likely underpowered. Furthermore, most studies did not report the proportion of patients that are deemed high-risk in their respective cohorts, and therefore studies with low prophylactic antibiotic usage may indeed have covered for all high-risk patients. In fact, our meta-analysis on all eligible studies investigating IE incidence after dental treatment with appropriate antibiotic cover in 100% of patients revealed a striking 0% incidence of IE. More studies are required to increase the sample size which will allow future meta-analyses to reach sufficient power to draw further conclusions.

Our meta-analysis of the six eligible studies with suitable data eliciting a pooled total of 11,095 cases and the same number of matched controls showed that IE patients are significantly more likely to have undergone recent dental procedures (RR = 1.12, 95% CI 1,00 to 1.26, $I^2 = 0\%$). We have selected three months as the cut off for recent dental procedures due to the relatively long incubation period and prolonged duration of symptoms of IE, in accordance with the majority of previously published studies (Table 2). These findings support the notion that dental procedures may increase the risk of IE and argue for antibiotic prophylaxis. As many of these studies utilised a case-crossover design (self-controls), this is unlikely to be due to other existing comorbidities. Indeed, studies assessing the incidence of IE before and after the 2007 American Heart Association (AHA) guideline changes found the sharp decline in prophylactic antibiotic use to be associated with a significant increase in IE incidence post dental procedures (38). Dental procedures such as tooth extraction have been shown to cause bacteraemia in 100% of adults (8, 9). Studies in the UK interrogating the incidence of IE after the 2008 NICE guideline changes which advised against routine prophylactic antibiotic use for dental procedures in at-risk patients similarly found an increase in incidence of oral streptococcus IE but not overall IE (39). This is consistent with our findings where oral streptococcus, mainly consisting of *Streptococcus*

For peer review only - http://bmjopen.bmj.com/site/about/guidelines.xhtml

viridans group, are the major cause of IE after dental procedures whereas *Staphylococcus* species are the more common cause in general IE patients.

Recently, four important studies investigating IE, dental procedures and antibiotic prophylaxis were published (15-18) after the inclusion deadline of the present systematic review and meta-analysis as per prospectively registered on PROSPERO. In a US case-crossover study of 7,951,972 patients with employer-provided health cover, a strong temporal association was found between invasive dental procedures in the preceding four weeks and IE in high-risk patients, with an odds ratio (OR) of 2.00 (95% CI 1.59 to 2.52, P = 0.002) (16). The association was strongest with certain dental procedures including dental extractions (OR = 11.08, 95% CI 7.34 to 16.74, P < 0.0001) and oral-surgical procedures (OR = 50.77, 95% CI 20.79 to 123.98, P < 0.0001). Strikingly, in that study the author also found that prophylactic antibiotic administration reduced IE incidence after dental procedures (OR = 0.49, 95% CI 0.29 to 0.85, P = 0.01) including dental extractions (OR = 0.13, 95% CI 0.03 to 0.34, P < 0.0001) and oral-surgical procedures (OR = 0.09, 95% CI 0.01 to 0.35, P = 0.002), particularly in high-risk as opposed to moderate or low-risk patients (16). In another elegant case-crossover study of 1,678,190 Medicaid patients (basic medical and dental cover), similar findings were observed where recent dental extractions (OR = 14.17, 95% CI 5.40 to 52.11, P < 0.0001) or oral surgery (OR = 29.98, 95% CI 9.62 to 119.34, P < 0.0001) within 30 days is associated with increased IE incidence, and that prophylactic antibiotic administration for dental procedure reduced IE incidence (OR = 0.20, 95% CI 0.06 to 0.53, P < 0.0001) (17). The number needed to treat with antibiotic prophylaxis to prevent one case of IE was estimated to range between 71 to 244 depending on the type of dental procedure. Finally, in a UK-based study of 14,731 patients by the same group investigating various different procedures and IE, dental procedures including extraction and surgical tooth removal were

For peer review only - http://bmjopen.bmj.com/site/about/guidelines.xhtml

found to be associated with increased IE risk (OR = 2.14, 95% CI 1.22 to 3.76, P = 0.047) (15). In a South Korean study of 62,019 patients with cardiac implantable electronic devices, invasive dental procedures were found to be associated with increased risk of IE (OR = 1.75, 95% CI 1.48 to 2.05, P < 0.001), with a mean time interval of around 60 days between receiving dental procedures and subsequently developing IE (18). Overall, these findings are largely consistent with our systematic review and meta-analysis, where IE patients are associated with increased risk of having undergone recent dental procedures in the preceding three months (RR = 1.12; 95% CI 1.00 to 1.26, I^2 = 0%, P = 0.048), and that across all the studies where 100% of the patients received antibiotic prophylaxis, none of the high risk patients developed IE. Importantly, the four new studies were not included in the results of our systematic review and meta-analysis due to being published after the pre-established search date as registered on PROSPERO. This is ideal as these studies have a very large sample size, one of which included 7,951,972 patients which is larger than all previous studies published in the last 50 years included in our meta-analysis combined. The striking concordance of our meta-analysis of all previous evidence to the recent large cohort studies indicate that there is an association between dental procedures and IE, and support the aforementioned AHA and ESC guidelines advocating the use of antibiotic prophylaxis in high-risk patients for dental procedures.

For peer review only

Limitations

Whilst our systematic review and meta-analysis provide an important appraisal and synthesis of all the available evidence on the association of dental procedures and IE in the context of prophylactic antibiotic use, there are important limitations. These include the differing patient characteristics between studies which may contribute to inter-study heterogeneity. Due to the

For peer review only - http://bmjopen.bmj.com/site/about/guidelines.xhtml

update in AHA and ESC guidelines in 2007 and 2009 respectively, the use of prophylactic antibiotics may differ between studies performed in different years, which may confound the association of invasive dental procedures and IE. Further limitations include the lack of randomised controlled trials available in the literature and therefore studies included in this systematic review and meta-analysis mainly consist of observational cohort studies and surveys. In the comparison of IE patients with controls, case-control studies are susceptible to confounding factors, thus causality between dental procedures and IE cannot be definitively established. Due to the rarity of IE, a large sample size is required to allow sufficient statistical power to detect any potential association, if any, between prophylactic antibiotic use for recent dental procedures and IE incidence. Indeed, whilst none of the individual included studies found a statistically significant difference in the incidence of recent dental procedures in IE patients compared to controls likely due to being underpowered, our meta-analysis demonstrated that IE patients are more likely to have undergone recent dental manipulation. The efficacy of prophylactic antibiotics, shown by some studies to be 100% in our meta-analysis, may further hide the association between IE and recent dental procedures. Another potential limitation is that studies performed in different times may record the casual organism differently, due to changes in classification of some types of bacteria, particularly enterococci and HACEK organisms. Furthermore, whilst some studies directly assessed dental records when gathering data on dental procedures, others relied on self-report by patients which is prone to recall bias. Similarly for data on prophylactic antibiotic use, some studies utilised patient records while others may rely on patient recollection or simply assume that all high-risk patients would have been offered antibiotic prophylaxis, therefore may be difficult to accurately verify.

Conclusions

For peer review only

Overall, our findings in this systematic review and meta-analysis indicate that prophylactic antibiotics for dental procedures may be of benefit in preventing IE in high-risk patients. Given the logistic difficulties of conducting randomised control trials for prophylactic antibiotic use and IE, further well-designed cohort studies will be invaluable to address this very important issue.

For peer review only

For peer review only - http://bmjopen.bmj.com/site/about/guidelines.xhtml

Declarations

Funding

Not applicable.

Conflicts of interest/Competing interests

The authors declare no conflicting interest.

Ethics approval

Not applicable.

Consent to Participate

Not applicable.

Consent for publication

Not applicable.

Author contributions

S.S.H.L, E.J, J.S.Y.H, and E.G.L.J were involved in the conceptualization of the study, designed the study, collected and analysed the data. All authors contributed to writing the manuscript, and approved for the final version of the paper to be published.

Data availability statement

All data relevant to the study are included in the article or uploaded as supplementary information. No additional data available.

Patient and Public Involvement

None.

For peer review only

For peer review only - http://bmjopen.bmj.com/site/about/guidelines.xhtml

Outcome	Number of studies	N	Proportion (95% CI)	I^2 value	P-value
**Dental procedures in IE patients**	**28**	**154582**	**0.11 (0.08-0.15)**	**99.5%**	**-**
Year of patient recruitment					0.372
Before 2007	19		0.12 (0.09-0.17)	80.1%	
After 2007	9		0.09 (0.04-0.18)	99.8%	
Continent of publication					0.989
Asia	9		0.10 (0.04-0.22)	97.7%	
North America	6		0.12 (0.08-0.17)	55.8%	
Europe	12		0.12 (0.07-0.19)	99.2%	
Australia	1		0.11 (0.05-0.21)	-	
**Streptococcus bacteraemia**	**16**	**3994**	**0.36 (0.28-0.44)**	**93.7%**	**-**
Dental procedures proportion					0.003
All patients had dental procedures	3		0.61 (0.43-0.76)	68.6%	
Not all patients had dental procedures	13		0.32 (0.25-0.39)	93.7%	

For peer review only - http://bmjopen.bmj.com/site/about/guidelines.xhtml

**Streptococcus viridans bacteraemia**	**23**	**3363**	**0.30 (0.22-0.40)**	**91.7%**	**-**
Dental procedures proportion					0.003
All patients had dental procedures	6		0.69 (0.46-0.85)	63.7%	
Not all patients had dental procedures	17		0.21 (0.17-0.26)	89.0%	
**Staphylococcus bacteraemia**	**25**	**5215**	**0.23 (0.18-0.30)**	**83.2%**	**-**
Dental procedures proportion					0.002
All patients had dental procedures	6		0.03 (0.01-0.14)	0%	
Not all patients had dental procedures	19		0.29 (0.25-0.34)	85.9%	
**Enterococcus bacteraemia**	**21**	**4767**	**0.07 (0.05-0.11)**	**76.6%**	**-**
Dental procedures proportion					0.355
All patients had dental procedures	4		0.003 (0.00-0.79)	0%	
Not all patients had dental procedures	17		0.08 (0.06-0.11)	79.0%	
**HACEK bacteraemia**	**24**	**5163**	**0.12 (0.10-0.16)**	**82.5%**	**-**
Dental procedures proportion					0.385
All patients had dental procedures	5		0.05 (0.004-0.36)	0.0%	
Not all patients had dental procedures	19		0.13 (0.10-0.16)	85.8%	

For peer review only

**Negative bacteraemia**	**23**	**5062**	**0.11 (0.07-0.16)**	**93.6%**	**-**
Dental procedures proportion					0.411
All patients had dental procedures	5		0.06 (0.01-0.25)	54.7%	
Not all patients had dental procedures	18		0.12 (0.08-0.18)	94.9%	
**IE in high-risk patients who underwent dental procedures**	**4**	**413**	**0.00 (0.00-1.00)**	**0%**	**-**

Table 1: Results of the meta-analyses performed with subgroup analysis

For peer review only

**Author, Year, Country**	**Study type**	**Population**	**Sample size**	**Type of dental procedure**	**Dental procedure (%)**	**Prophylactic abx (%)**	**Study duration**	**Duration between dental procedure and IE**
Takeda et al., 2005, Japan	Retrospective cohort	CHD with IE	183	Not specified	21.00%	18.40%	1971-1998	NR
Durack et al., 1983, US	Case series	Antibiotic prophylaxis failure, IE post dental procedures	52	Not specified	92.00%	100%; 12% had AHA recommended regimen	1979	5 weeks (median)
Porat Ben-Amy et al., 2009, Israel	Retrospective case-crossover	IE	170	Tooth extraction, periodontal treatment, root canal treatment, implant insertion, others	12.00%	50% eligible patients	2003-2005	3 months
Hricak et al., 2013, Slovakia	Prospective cohort	IE	606	Not specified	13.20%	NR	1984-2006	NR

For peer review only - http://bmjopen.bmj.com/site/about/guidelines.xhtml

Carmona et al., 2003, Spain	Retrospective cohort	IE	115	Tooth extraction, Fillings, Scaling, Professional cleaning	5.20%	NR	1997-2001	3 months
Delahaye et al., 2016, France	Prospective cohort	IE	318	Not specified	2.50%	NR	2005-2016	3 months
Strom et al., 1998, US	Population-based case-control study	Case - IE; control - no IE	273 case 273 control	Dental hygiene care, filling periodontal treatment, restorative dentistry, extraction, endodontic treatment, treatment of tooth abscess, mouth or gingival surgery, other	16.8% in cases; 14.3% in controls	Cases: 5.1%; Controls: 1.1%	1988-1990	2 months
Aziz et al., 2010, US	Retrospective cohort	IE undergoing heart surgery	50	Not specified	16.00%	NR	2000-2003	NR

For peer review only - http://bmjopen.bmj.com/site/about/guidelines.xhtml

Chu et al., 2004, New Zealand	Retrospective cohort	IE	65	Not specified	10.80%	NR	1997-2002	NR
Chen et al., 2015, Taiwan	Population-based case-control study	IE	713	Tooth extraction, surgery, dental scaling, periodontal treatment, and endodontic treatment	Cases: 12.2%; controls: 10.8%	2.80%	1999-2012	3 months
Tubiana et al., 2017, France	Population-based cohort and case-crossover study	Positioning or replacement of prosthetic heart valves, with IE	138,876	Invasive and non-invasive	49.9%; 26.0% of procedures were invasive	50.1% eligible patinets	2008-2014	3 months
Imperiale et al., 1990, US	Case-control study	Case - IE after dental procedure with high risk cardiac lesion; control - no IE after dental procedure	8 cases 24 controls	Cleaning, filing, extraction	NA	Cases: 12.5%; Controls 62.5%	1980-1986	3 months

For peer review only - http://bmjopen.bmj.com/site/about/guidelines.xhtml

Sett et al., 1993, Canada	Retrospective cohort	IE in procine bioprothesis patients	56	Not specified	NR	NR	1975-1988	NR
Cukingnan et al., 1983, US	Retrospective cohort	Early valve replacement for IE	42	Not specified	5.00%	NR	1969-1983	NR
Grover et al., 1991, India	Retrospective cohort	IE	19	tooth extraction	5.30%	0%	1982-1989	NR
Mudhumitha et al., 2018, India	Retrospective cohort	IE	120	Not specified	3.30%	NR	2010-2015	3 months
Lacassin et al., 1995, France	Case-control study	IE	171 cases, 171 controls	Not specified	Cases: 22%; controls: 19%	NR	1990-1991	3 months
Duval et al., 2006, France	Retrospective survey	General population	2805	Not specified	0.70%	26.30%	1998	1 month

For peer review only - http://bmjopen.bmj.com/site/about/guidelines.xhtml

Chirillo et al., 2016, Italy	Prospective cohort	IE	677	Not specified	4.70%	40.60%	2007-2010	2 months
Krcmery et al., 2018, Slovakia	Prospective cohort	IE	180	Not specified	20.50%	NR	1984-2017	NR
Duval et al., 2017, France	Case-control study	Case - IE caused by oral streptococci; Control - IE caused by nonoral pathogens	73 cases, 192 controls	Not specified	8.80%	NR	2008-2013	3 months
Weinberger et al., 1990, Israel	Retrospective cohort	IE patients with mitral valve prolapse	19	Not specified	78.90%	0%	1970-1987	2 months
Martin et al., 2007, UK	Case series	IE cases after dental procedure with successful litigation	83	Exodontia, scaling, endodontics and minor oral surgery	NA	16.90%	1983-2005	Mean 9 days (range 2-22 days)
Santinga et al., 1976, US	Case series	IE and prosthetic heart valve	11	Not specified	9.10%	NR	1976	NR

For peer review only

Siegman-Igra et al., 2010, Israel	Prospective cohort	Culture-positive IE	212	Not specified	8.00%	NR	1995-1998; 2003-2005	3 months
Carmona et al., 2002, Spain	Retrospective cohort	IE	103	extraction, scaling, filling	5.80%	0%	1997-1999	NR
Luk et al., 2014, Canada	Retrospective cohort	IE treated with surgical valve explant	209	Not specified	13.90%	NR	2001-2012	NR
Kim et al., 2019, Korea	Retrospective cohort	IE in cancer and non-cancer	170	Not specified	4.10%	NR	2011-2015	NR
Smith et al., 1976, UK	Retrospective cohort	IE	78	Not specified	5.10%	NR	1969-1972	NR
Ballesta et al., 2022, Spain	Retrospective cohort	IE	101	Not specified	21.00%	0%	2000-2017	NR

For peer review only - http://bmjopen.bmj.com/site/about/guidelines.xhtml

Dominguez et al., 2016, Spain	Prospective cohort	IE	1807	Not specified	7.00%	38.50%	2008-2013	NR
Loupa et al., 2004, Greece	Prospective cohort	IE	101	Not specified	13.00%	NR	1997-2000	3 months
Chen et al., 2018, Taiwan	Case series	IE	9120	dental cleaning, scaling and root planing, simple extraction, complicated extraction, odontectomy in both simple case and complicated case, and periodontal surgery	Cases: 2.7%; controls: 2.7%	NR	2004-2013	3 months

Table 2: Characteristics of included studies on patients with infective endocarditis

For peer review only

Author, year country	Study type	Population	Sample size	Type of dental procedure	IE (%)	Prophylactic antibiotics (%)	Study duration	Duration between dental procedure and IE
Russo et al., 2000, Italy	Prospective cohort	Prosthetic heart valve patients undergoing dental procedures	104	NR	0	100%	NR	3 months
Chang et al., 2017, Taiwan	Retrospective cohort	SLE patients	12102	NR	NR	NR	NR	30 days
Findler et al., 2014, Israel	Retrospective cohort	high risk for IE and underwent dental implant placement	13	All had dental implants	0	100%	1995-2012	NA
Tzukert et al., 1986, Israel	prospective cohort	Prosthetic heart valves, high risk IE	90	scaling, fillings, extraction, root canal, crowns, bridges	0	100%	1967-1982	NA
Littner et al., 1986, Israel	Retrospective cohort	Susceptible patients to IE	206	extraction, drainage of abscess, biopsy, filling, oral surgery, crown, bridge, scaling	0	100%	1982-1986	NA

Table 3: Characteristics of studies on patients undergoing dental procedures

For peer review only - http://bmjopen.bmj.com/site/about/guidelines.xhtml

Figure legends:

Figure 1. Proportion of dental procedures in case vs control in case-control or crossover studies.

Figure 2. Proportion of IE in moderate and high risk patients who underwent dental procedures.

For peer review only

References

1. Hsu CN, Wang JY, Tseng CD, Hwang JJ, Hsueh PR, Liau CS. Clinical features and predictors for mortality in patients with infective endocarditis at a university hospital in Taiwan from 1995 to 2003. Epidemiol Infect. 2006;134(3):589-97.
2. Muñoz P, Kestler M, De Alarcon A, Miro JM, Bermejo J, Rodríguez-Abella H, et al. Current Epidemiology and Outcome of Infective Endocarditis: A Multicenter, Prospective, Cohort Study. Medicine (Baltimore). 2015;94(43).
3. Rajani R, Klein JL. Infective endocarditis: A contemporary update. Clin Med (Lond). 2020;20(1):31-5.
4. Richey R, Wray D, Stokes T. Prophylaxis against infective endocarditis: summary of NICE guidance. Bmj. 2008;336(7647):770-1.
5. Dajani AS, Taubert KA, Wilson W, Bolger AF, Bayer A, Ferrieri P, et al. Prevention of bacterial endocarditis. Recommendations by the American Heart Association. Circulation. 1997;96(1):358-66.
6. Wilson W, Taubert KA, Gewitz M, Lockhart PB, Baddour LM, Levison M, et al. Prevention of infective endocarditis: guidelines from the American Heart Association: a guideline from the American Heart Association Rheumatic Fever, Endocarditis, and Kawasaki Disease Committee, Council on Cardiovascular Disease in the Young, and the Council on Clinical Cardiology, Council on Cardiovascular Surgery and Anesthesia, and the Quality of Care and Outcomes Research Interdisciplinary Working Group. Circulation. 2007;116(15):1736-54.
7. Habib G, Lancellotti P, Antunes MJ, Bongiorni MG, Casalta J-P, Del Zotti F, et al. 2015 ESC Guidelines for the management of infective endocarditis: The Task Force for the Management of Infective Endocarditis of the European Society of Cardiology (ESC)Endorsed by: European Association for Cardio-Thoracic Surgery (EACTS), the European Association of Nuclear Medicine (EANM). European Heart Journal. 2015;36(44):3075-128.
8. Lockhart PB. The risk for endocarditis in dental practice. Periodontol 2000. 2000;23:127-35.
9. Barbosa M, Prada-López I, Álvarez M, Amaral B, de los Angeles CD, Tomás I. Post-tooth extraction bacteraemia: a randomized clinical trial on the efficacy of chlorhexidine prophylaxis. PLoS One. 2015;10(5):e0124249.
10. Cahill TJ, Prendergast BD. Current controversies in infective endocarditis. F1000Res. 2015;4.
11. Embil JM, Chan KL. The American Heart Association 2007 endocarditis prophylaxis guidelines: a compromise between science and common sense. Can J Cardiol. 2008;24(9):673-5.
12. Daly CG. Antibiotic prophylaxis for dental procedures. Aust Prescr. 2017;40(5):184-8.
13. Rutherford SJ, Glenny AM, Roberts G, Hooper L, Worthington HV. Antibiotic prophylaxis for preventing bacterial endocarditis following dental procedures. Cochrane Database Syst Rev. 2022;5(5):Cd003813.
14. Cahill TJ, Harrison JL, Jewell P, Onakpoya I, Chambers JB, Dayer M, et al. Antibiotic prophylaxis for infective endocarditis: a systematic review and meta-analysis. Heart. 2017;103(12):937-44.
15. Thornhill MH, Crum A, Campbell R, Stone T, Lee EC, Bradburn M, et al. Temporal association between invasive procedures and infective endocarditis. Heart. 2023;109(3):223-31.
16. Thornhill MH, Gibson TB, Yoon F, Dayer MJ, Prendergast BD, Lockhart PB, et al. Antibiotic Prophylaxis Against Infective Endocarditis Before Invasive Dental Procedures. J Am Coll Cardiol. 2022;80(11):1029-41.
17. Thornhill MH, Gibson TB, Yoon F, Dayer MJ, Prendergast BD, Lockhart PB, et al. Endocarditis, invasive dental procedures, and antibiotic prophylaxis efficacy in US Medicaid patients. Oral Dis. 2023.
18. Kim JY, Park SJ, Lee SH, Seo GH, Jang SW. Risk of infective endocarditis associated with invasive dental procedures in patients with cardiac rhythm devices. Europace. 2022;24(12):1967-72.
19. Barnett R. Infective endocarditis. Lancet. 2016;388(10050):1148.

For peer review only

20. Chambers ST, Murdoch D, Morris A, Holland D, Pappas P, Almela M, et al. HACEK infective endocarditis: characteristics and outcomes from a large, multi-national cohort. PLoS One. 2013;8(5):e63181.
21. Birlutiu V, Birlutiu RM, Costache VS. Viridans streptococcal infective endocarditis associated with fixed orthodontic appliance managed surgically by mitral valve plasty: A case report. Medicine (Baltimore). 2018;97(27):e11260.
22. Khaledi M, Sameni F, Afkhami H, Hemmati J, Asareh Zadegan Dezfuli A, Sanae M-J, et al. Infective endocarditis by HACEK: a review. Journal of Cardiothoracic Surgery. 2022;17(1):185.
23. Tubiana S, Blotière P-O, Hoen B, Lesclous P, Millot S, Rudant J, et al. Dental procedures, antibiotic prophylaxis, and endocarditis among people with prosthetic heart valves: nationwide population based cohort and a case crossover study. BMJ. 2017;358:j3776.
24. Santinga JT, Fekety RF, Bottomley WK, Else B, Willis PW. Antibiotic prophylaxis for endocarditis in patients with a prosthetic heart valve. The Journal of the American Dental Association. 1976;93(5):1001-5.
25. Strom BL, Abrutyn E, Berlin JA, Kinman JL, Feldman RS, Stolley PD, et al. Dental and cardiac risk factors for infective endocarditis. A population-based, case-control study. Ann Intern Med. 1998;129(10):761-9.
26. Lacassin F, Hoen B, Leport C, Selton-Suty C, Delahaye F, Goulet V, et al. Procedures associated with infective endocarditis in adults. A case control study. Eur Heart J. 1995;16(12):1968-74.
27. Chang YS, Chang CC, Chen YH, Chen WS, Chen JH. Risk of infective endocarditis in patients with systemic lupus erythematosus in Taiwan: a nationwide population-based study. Lupus. 2017;26(11):1149-56.
28. Littner MM, Kaffe I, Tamse A, Buchner A. New concept in chemoprophylaxis of bacterial endocarditis resulting from dental treatment. Oral Surgery, Oral Medicine, Oral Pathology. 1986;61(4):338-42.
29. Russo G, Corso LD, Biasiolo A, Berengo M, Pengo V. Simple and safe method to prepare patients with prosthetic heart valves for surgical dental procedures. Clin Appl Thromb Hemost. 2000;6(2):90-3.
30. Tzukert AA, Leviner E, Sela M. Prevention of infective endocarditis: Not by antibiotics alone: A 7-year follow-up of 90 dental patients. Oral Surgery, Oral Medicine, Oral Pathology. 1986;62(4):385-8.
31. Findler M, Chackartchi T, Regev E. Dental implants in patients at high risk for infective endocarditis: a preliminary study. Int J Oral Maxillofac Surg. 2014;43(10):1282-5.
32. Talha KM, Baddour LM, Thornhill MH, Arshad V, Tariq W, Tleyjeh IM, et al. Escalating incidence of infective endocarditis in Europe in the 21st century. Open Heart. 2021;8(2):e001846.
33. Tong SY, Davis JS, Eichenberger E, Holland TL, Fowler VG, Jr. Staphylococcus aureus infections: epidemiology, pathophysiology, clinical manifestations, and management. Clin Microbiol Rev. 2015;28(3):603-61.
34. Remadi JP, Habib G, Nadji G, Brahim A, Thuny F, Casalta JP, et al. Predictors of death and impact of surgery in Staphylococcus aureus infective endocarditis. Ann Thorac Surg. 2007;83(4):1295-302.
35. Fukushima S, Hagiya H, Mizuta Y, Nakano Y, Takahara M, Okamoto K, et al. Multiple Deep-seated Dentofacial Abscesses Caused by Multidrug-resistant Viridans Group Streptococci. Intern Med. 2020;59(16):2067-70.
36. Smith A, Jackson MS, Kennedy H. Antimicrobial susceptibility of viridans group streptococcal blood isolates to eight antimicrobial agents. Scand J Infect Dis. 2004;36(4):259-63.
37. Tuohy M, Washington JA. Antimicrobial susceptibility of viridans group streptococci. Diagn Microbiol Infect Dis. 1997;29(4):277-80.

For peer review only

38. Thornhill MH, Gibson TB, Cutler E, Dayer MJ, Chu VH, Lockhart PB, et al. Antibiotic Prophylaxis and Incidence of Endocarditis Before and After the 2007 AHA Recommendations. Journal of the American College of Cardiology. 2018;72(20):2443-54.
39. Edwards C, Lacey A, Anderson M, Bodger O. 98 Are we seeing more cases of Infective Endocarditis after Nice CG64 Recommended Cessation of the use of Antibiotic Prophylaxis? The Welsh Experience. Heart. 2015;101(Suppl 4):A56-A7.

For peer review only

For peer review only - http://bmjopen.bmj.com/site/about/guidelines.xhtml

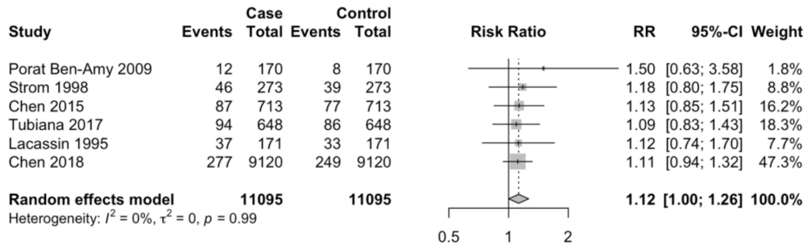

Figure 1. Proportion of dental procedures in case vs control in case-control or crossover studies

722x538mm (72 x 72 DPI)

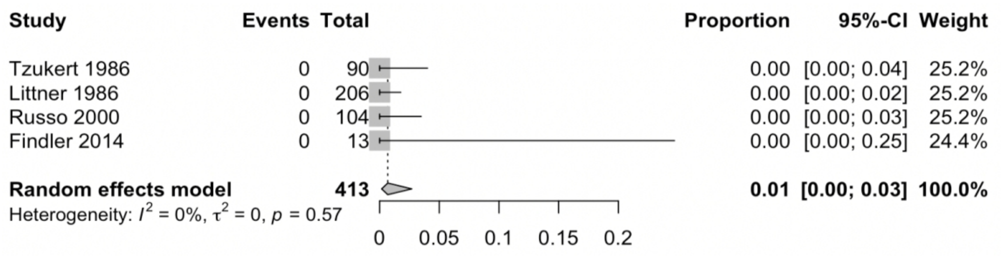

Figure 2. Proportion of IE in moderate and high risk patients who underwent dental procedures

542x136mm (72 x 72 DPI)

Supplementary Materials

Supplementary Table 1: Patient characteristics with moderate or high risk of infective endocarditis according to the AHA and ESC guidelines

**Moderate risk**	**High risk**
Uncorrected congenital cardiac conditions e.g. bicuspid aortic valve, coarctation of the aorta, patent ductus arteriosus, atrial or ventricular septal defects	Prosthetic heart valves or repaired valves using prosthetic material
Acquired valvular diseases e.g. Rheumatic heart disease	Previous history of endocarditis
Hypertrophic cardiomyopathy	Complex cyanotic congenital heart disease
	Systemic pulmonary shunts or conduits constructed via surgery

For peer review only

Supplementary Table 2: Search strategy by databases

**Database**	**Search term**
Medline	("dent*"[All Fields] OR "dontic*"[All Fields]) AND "endocarditis"[All Fields]
Embase	((“dent*” or “*dontic*”) and “endocarditis”).mp.
Scopus	TITLE-ABS-KEY (("dent*" OR "dontic") AND "endocarditis")
Clinicaltrials.gov	Endocarditis

For peer review only

Supplementary Table 3: Inclusion criteria

	**Inclusion criteria**
Population	• Adults age >18 years
Exposure/Intervention	• Any dental procedures 3 months prior to diagnosis of infective endocarditis such as tooth extraction, fillings, scaling, endodontal and periodontal treatment, implant insertion, root canal treatment
Comparison (if available)	• No dental procedure within 3 months of infective endocarditis
Outcome	• Diagnosis of infective endocarditis within 3 months of dental procedure

For peer review only

Supplementary Table 4: Quality assessment of included studies using the Newcastle-Ottawa Scale

**Author, Year, Country**	**Selection**				**Comparability**	**Outcome**			**Total quality score**
	Cohort studies: Representativeness of the exposed cohort	Selection of non-exposed cohort	Ascertainment of exposure	Demonstration that the current outcome of interest was not present at the start of study	Comparability of the cohorts on the basis of design or analysis	Ascertainment of outcome	Was follow up long enough for outcomes to occur	Adequacy of follow up of cohorts	
	Case control studies: Case definition adequate	Representativeness of cases	Selection of controls	Definition of controls	Comparability of cases and controls on the basis of design or analysis	Ascertainment of exposure	Same method of ascertainment for cases and controls	Non-response rate	
Aziz et al., 2010, US	★	★		★		★			4
Ballesta et al., 2022, Spain	★	★	★	★	★★	★	★		8
Carmona et al., 2002, Spain	★	★	★	★		★	★		6
Carmona et al., 2003, Spain	★	★	★	★	★	★	★		7
Chang et al., 2017, Taiwan	★	★	★	★	★★	★	★	★	8
Chen et al., 2015, Taiwan	★	★	★	★	★	★	★	★	8
Chen et al., 2018, Taiwan	★	★	★	★	★★	★	★	★	9

For peer review only

Chirillo et al., 2016, Italy	★	★	★	★		★	★	★	7
Chu et al., 2004, New Zealand	★	★		★		★	★		5
Cukingnan et al., 1983, US	★	★	★	★		★	★	★	7
Delahaye et al., 2016, France	★	★	★	★		★	★	★	7
Domingue z et al., 2016, Spain	★	★	★	★	★★	★	★		8
Durack et al., 1983, US				★			★		2
Duval et al., 2006, France	★	★	★	★	★	★	★	★	8
Duval et al., 2017, France	★	★	★	★	★★		★	★	8
Findler et al., 2014, Israel	★	★	★	★		★	★		7
Grover et al., 1991, India	★	★	★	★		★	★	★	7
Hricak et al., 2013, Slovakia	★	★	★	★		★	★	★	7
Imperiale et al., 1990, US	★	★	★	★	★★	★	★		8

For peer review only

Kim et al., 2019, Korea	★	★	★	★		★	★	★	7
Krcmery et al., 2018, Slovakia	★	★	★	★		★	★	★	7
Lacassin et al., 1995, France	★	★		★	★★		★		6
Littner et al., 1986, Israel	★			★		★	★		4
Loupa et al., 2004, Greece	★	★	★	★		★	★		6
Luk et al., 2014, Canada	★	★	★	★		★	★		6
Martin et al., 2007, UK	★		★	★		★	★		5
Mudhumitha et al., 2018, India	★	★	★	★		★	★	★	7
Porat Ben-Amy et al., 2009, Israel	★	★	★	★	★★	★	★		8
Russo et al., 2000, Italy	★	★		★			★		4
Santinga et al., 1976, US			★	★			★		3
Sett et al., 1993, Canada	★	★	★	★	★★	★	★	★	9

For peer review only

Siegman-Igra et al., 2010, Israel	★	★	★	★		★	★		6
Smith et al., 1976, UK	★			★			★		3
Strom et al., 1998, US	★	★	★	★	★★	★	★		8
Takeda et al., 2005, Japan	★	★	★	★		★	★		6
Tubiana et al., 2017, France	★	★	★	★	★★	★	★	★	9
Tzukert et al., 1986, Israel				★			★		2
Weinberger et al., 1990, Israel	★	★	★	★		★	★		6

For peer review only

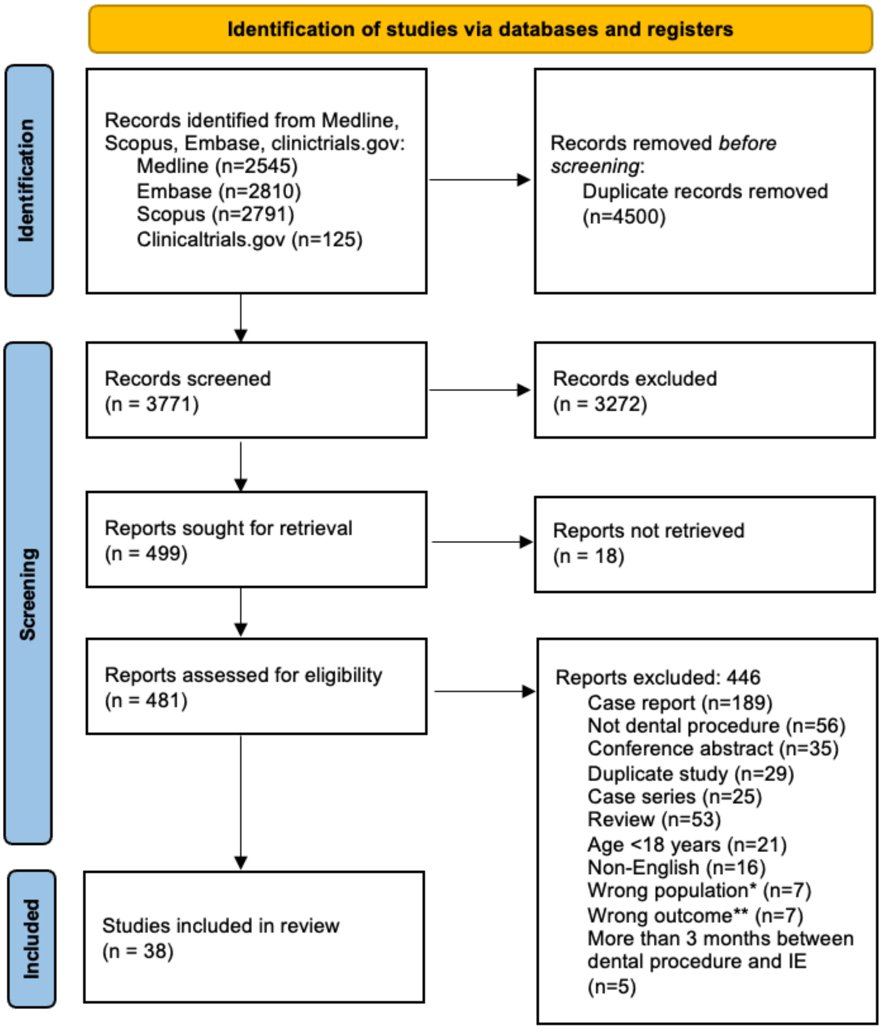

*"Wrong population" refers to studies that did not include patients with IE and dental procedures

**"wrong outcomes" refers to studies that did not report findings relevant to the conclusions of the present systematic review

Supplementary Figure 1. PRISMA flow diagram of the study selection process

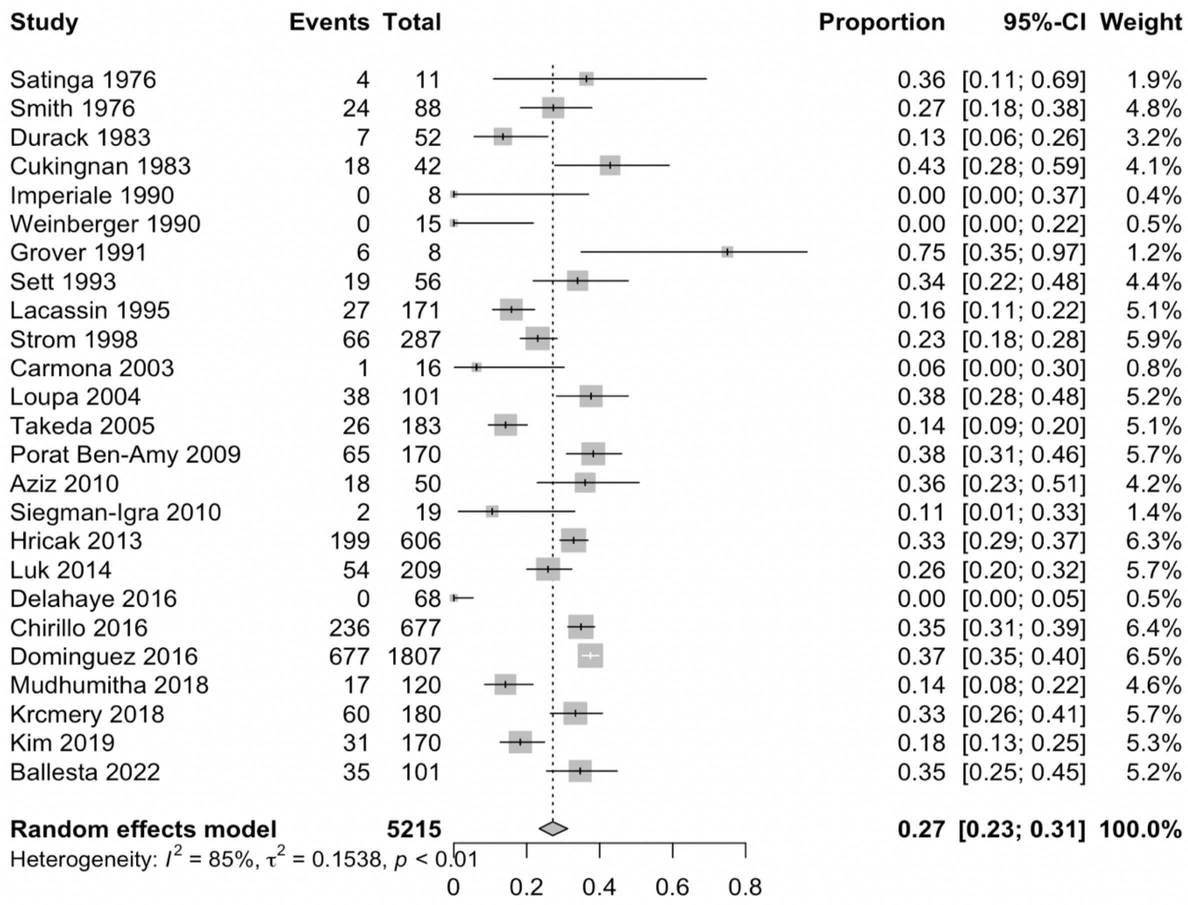

Supplementary Figure 2A. Proportion of *Staphylococcus* bacteraemia

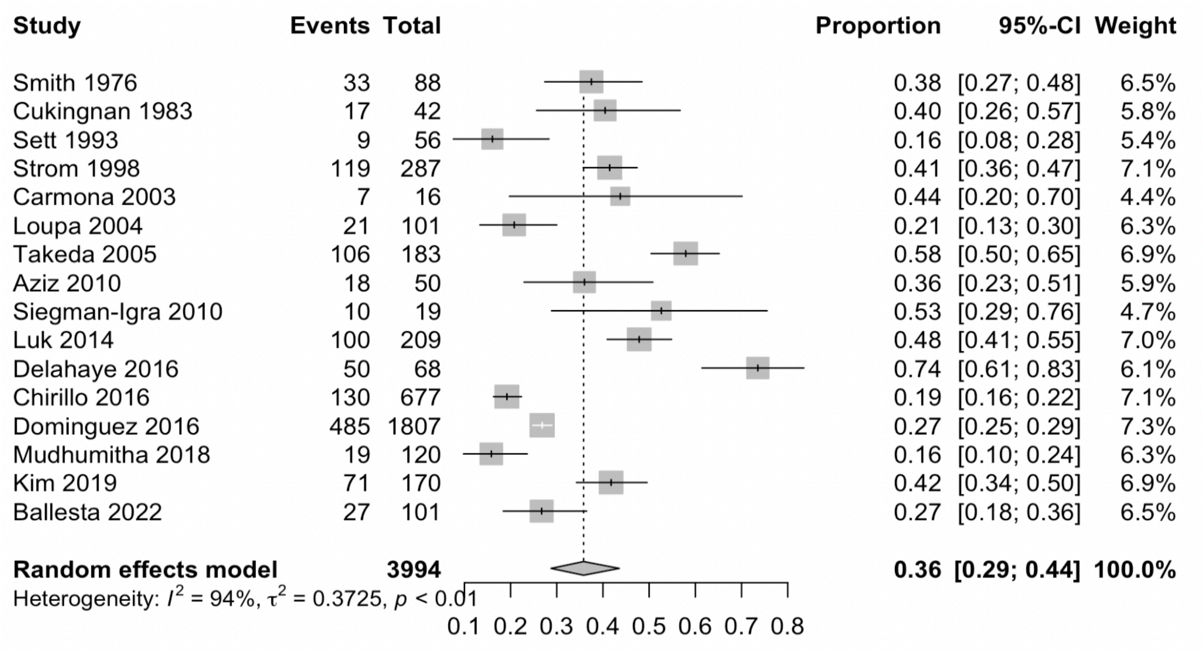

Supplementary Figure 2B. Proportion of *Streptococcus* bacteraemia

For peer review only

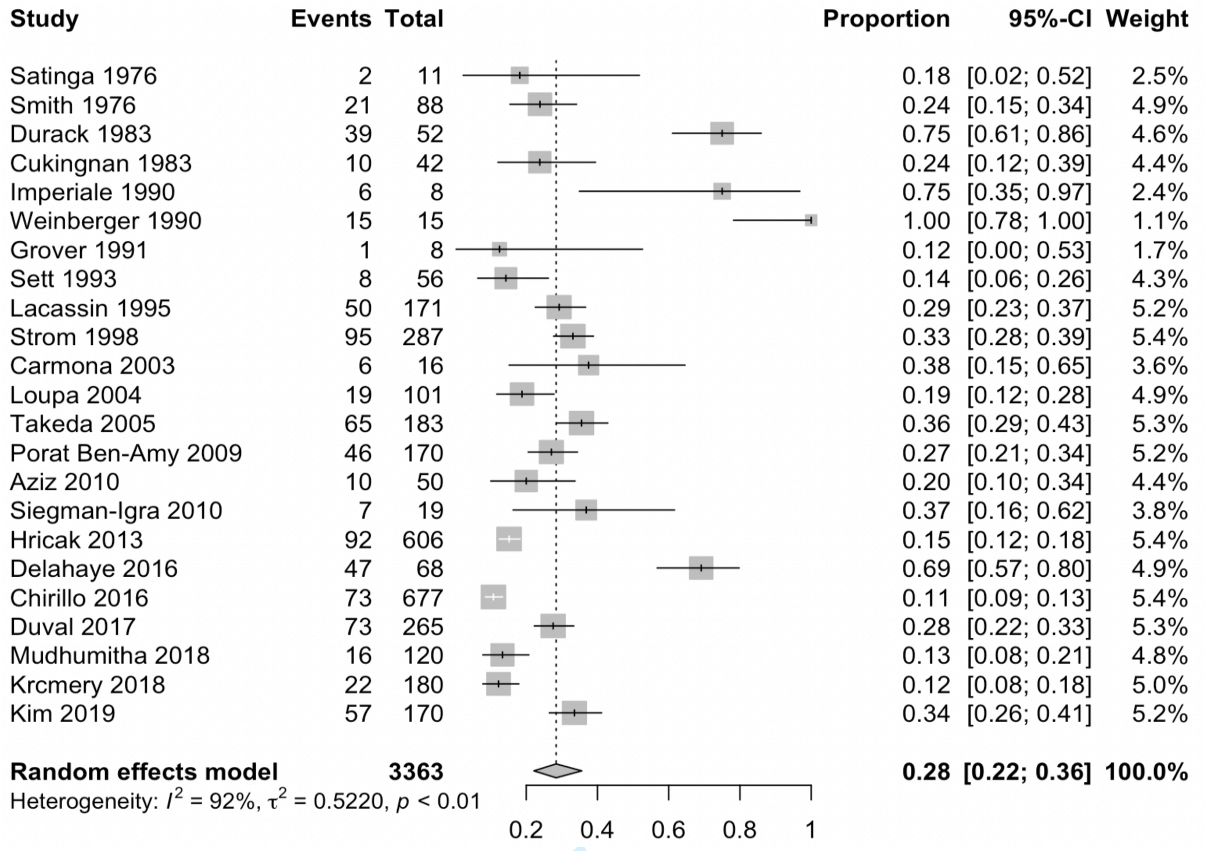

Supplementary Figure 2C. Proportion of *Streptococcus viridans* bacteraemia

review only

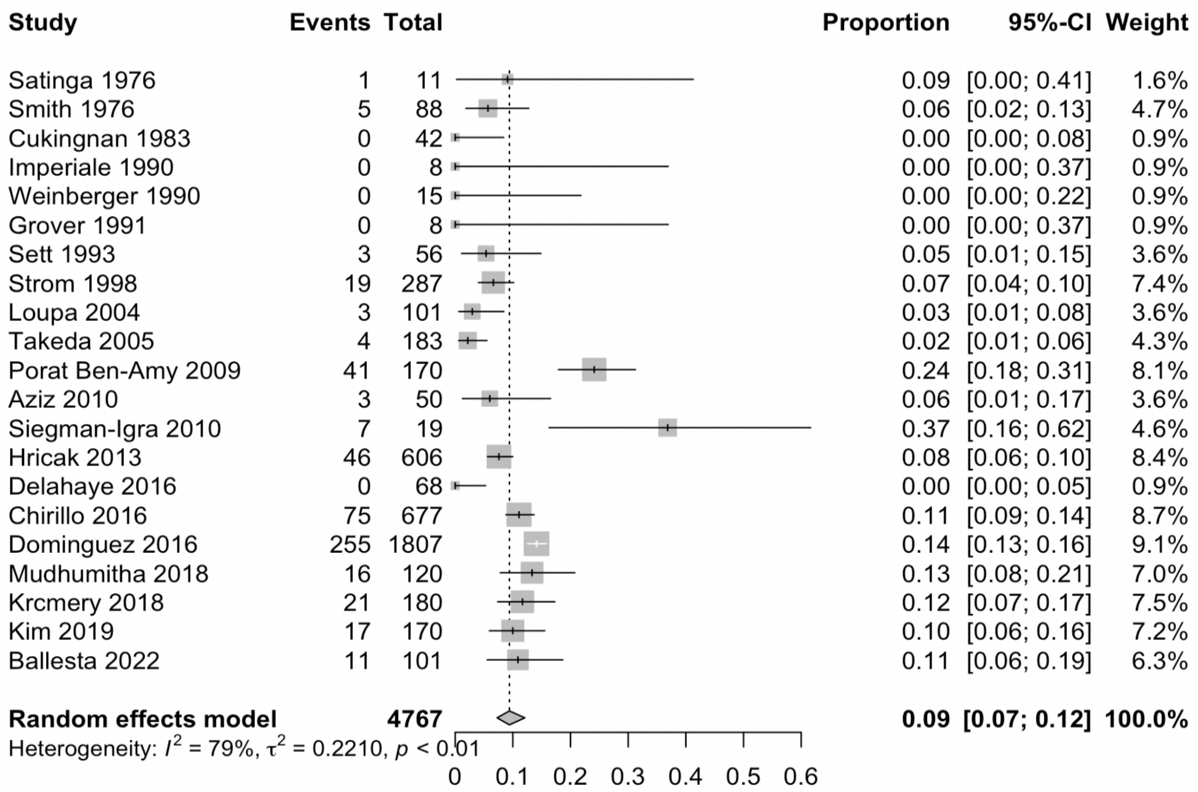

Supplementary Figure 2D. Proportion of *Enterococcus* bacteraemia

For peer review only

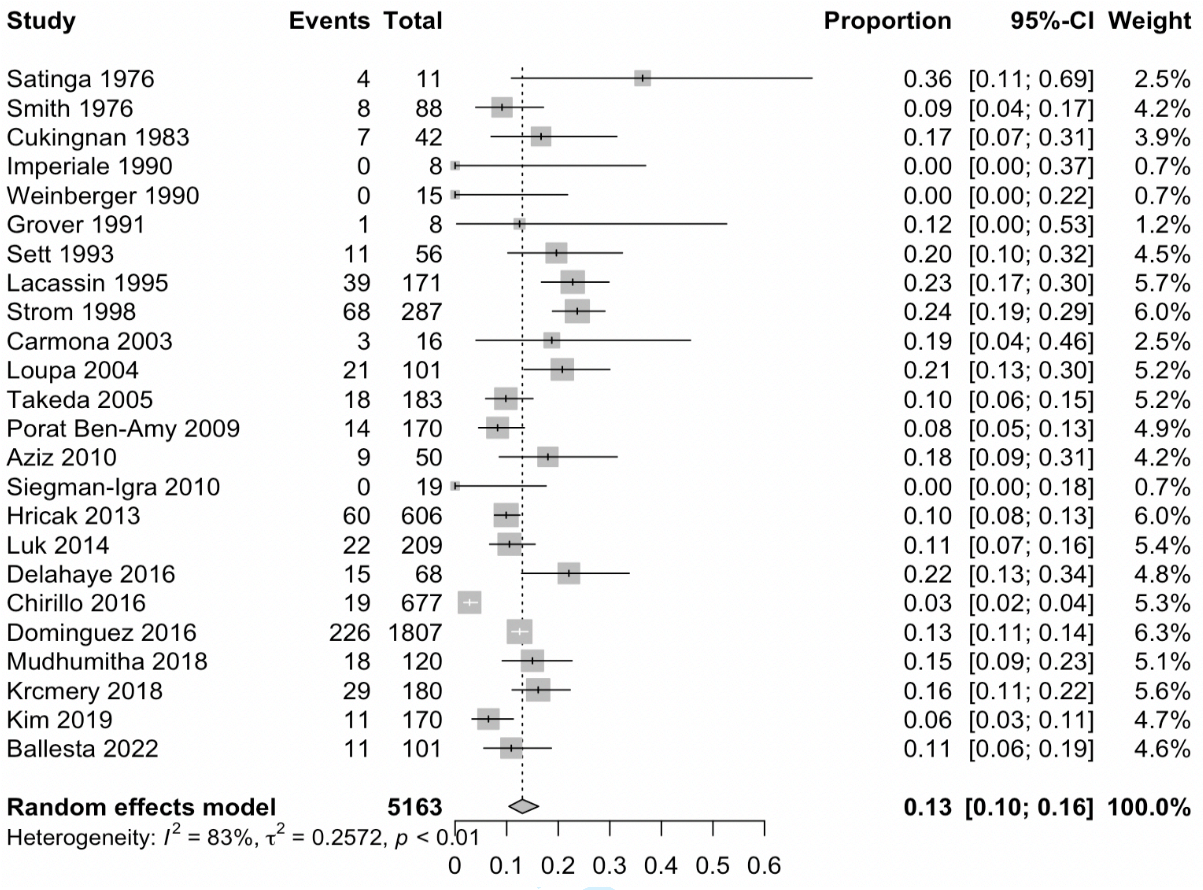

Supplementary Figure 2E. Proportion of HACEK and others bacteraemia

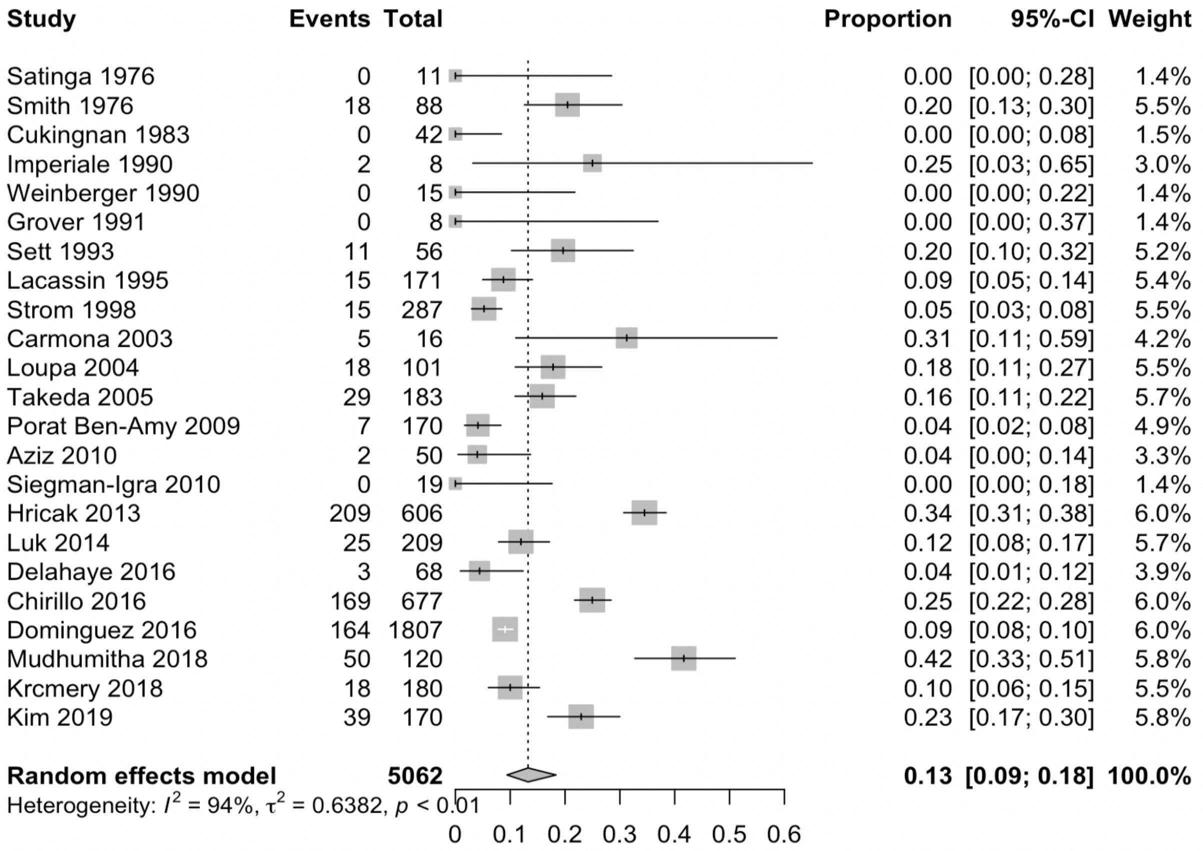

Supplementary Figure 2F. Proportion of culture negative infective endocarditis

review only

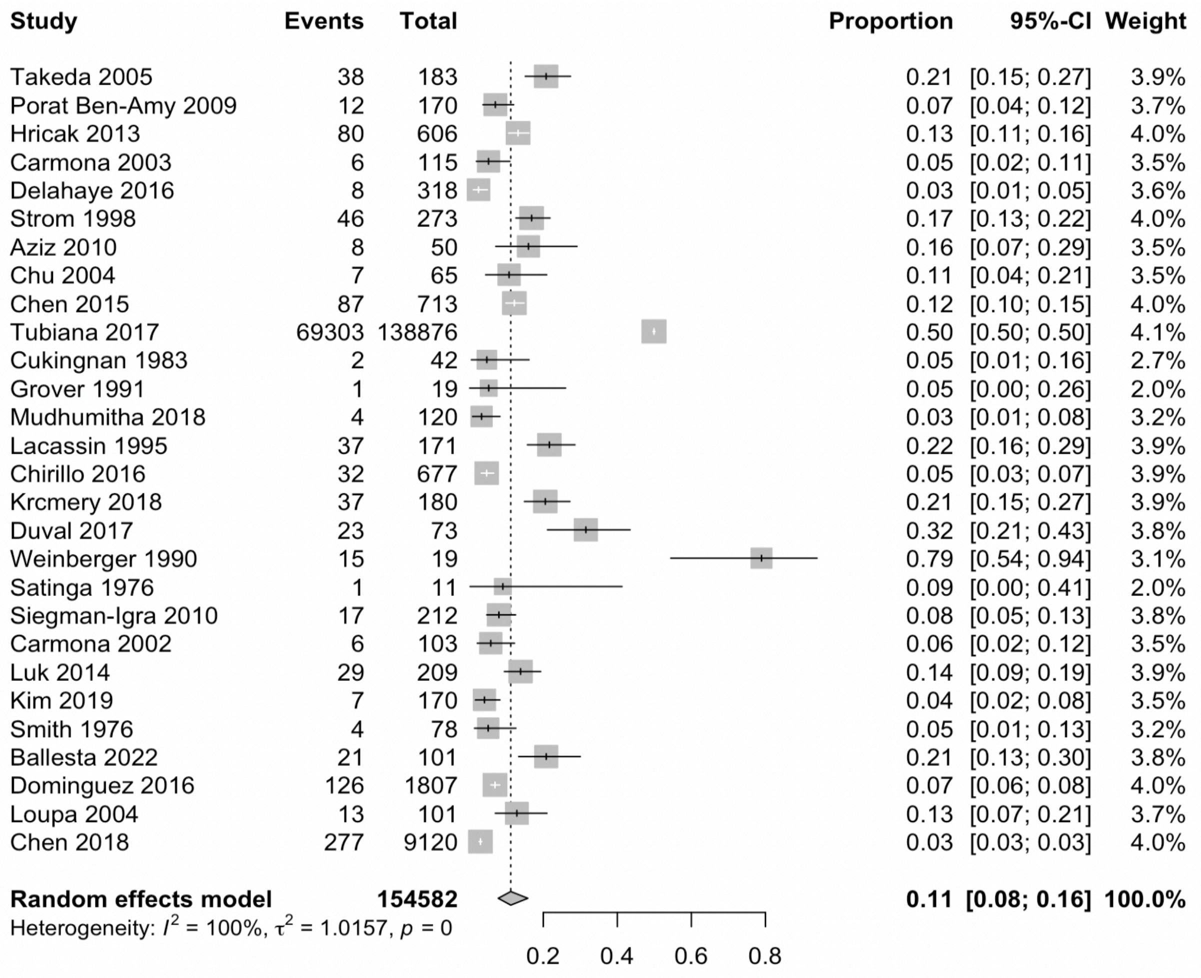

Supplementary Figure 3. Proportion of dental procedures in IE patients

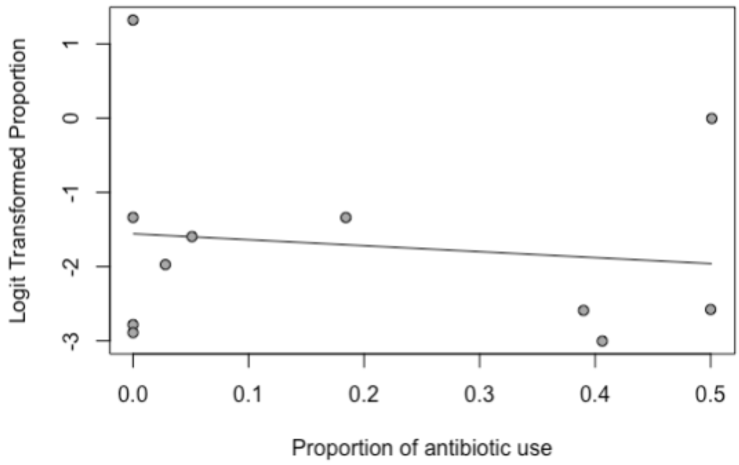

Supplementary Figure 4A: Meta-regression of proportion of infective endocarditis patients with recent dental procedure against proportion of antibiotic use (Intercept coefficient: -0.8091, p=0.6529)

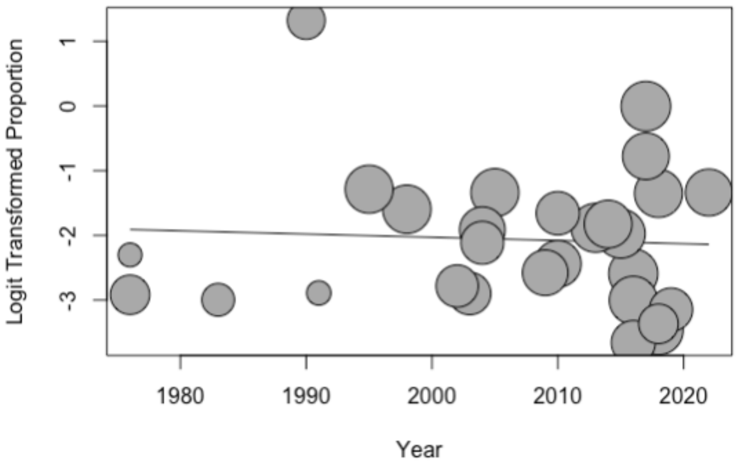

Supplementary Figure 4B: Meta-regression of proportion of infective endocarditis patients with recent dental procedure against the year of publication (Intercept coefficient = -0.005, p=0.7717)

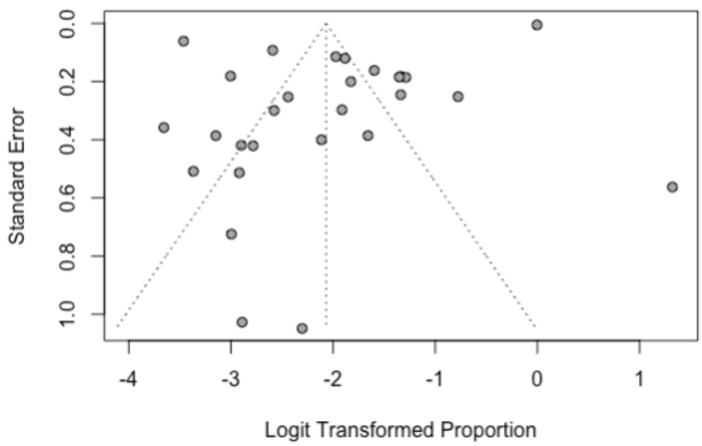

Supplementary Figure 5A: Funnel plot for proportion of IE patients who underwent recent dental procedures (Egger's test: t = -4.46, p = 0.0001)

For peer review only

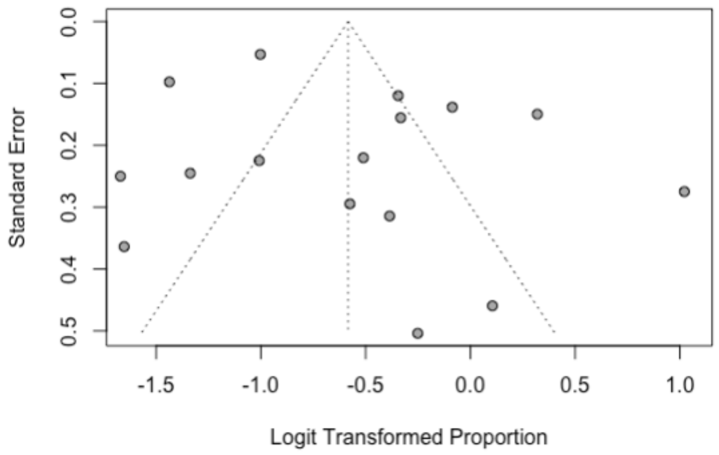

Supplementary Figure 5B: Funnel plot for proportion of Streptococcus (Egger's test: t = 1.36, p = 0.1968)

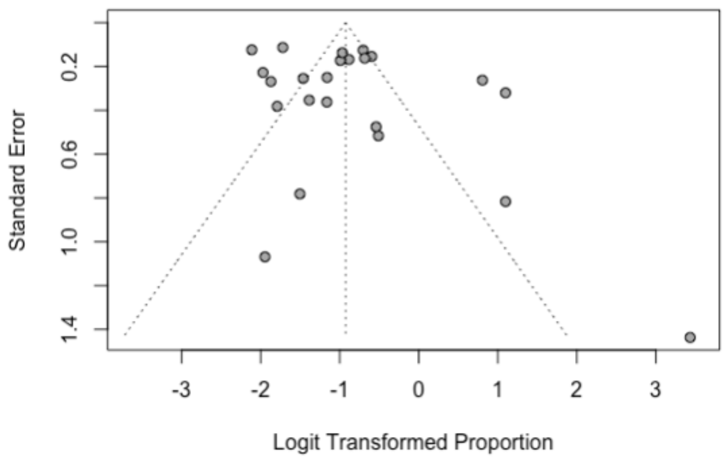

Supplementary Figure 5C: Funnel plot for proportion of Streptococcus Viridans

(Egger's test: t = 1.35, p = 0.1921)

For peer review only

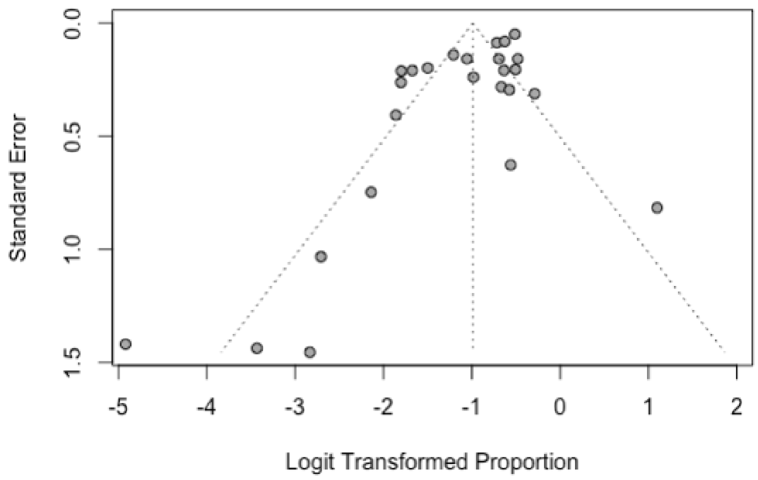

Supplementary Figure 5D: Funnel plot for proportion of Staphylococcus (Egger's test: t = -3.19, p = 0.0040)

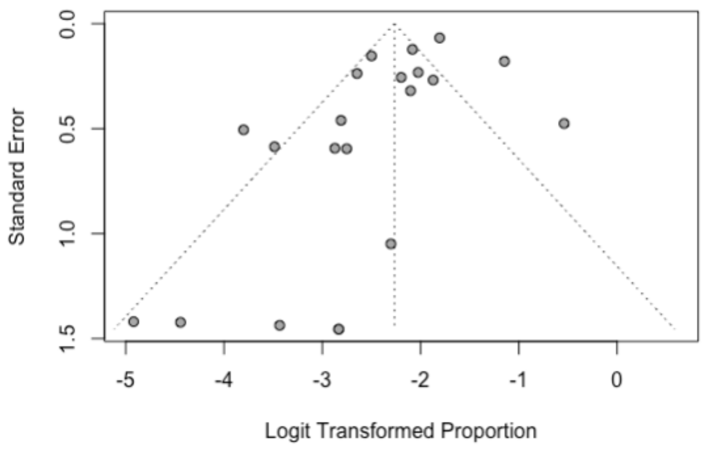

Supplementary Figure 5E: Funnel plot for proportion of Enterococcus (Egger's test: t = -2.40, p-value = 0.0268)

For peer review only

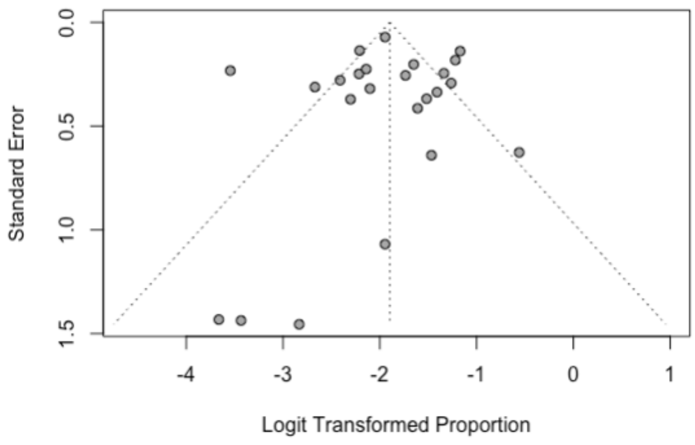

Supplementary Figure 5F: Funnel plot for proportion of HACEK (Egger's test: t = -0.23, p = 0.8239)

For peer review only

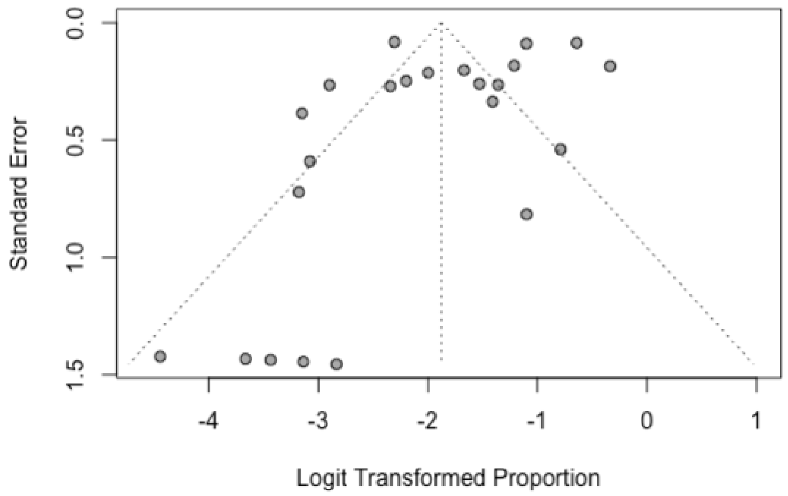

Supplementary Figure 5G: Funnel plot for proportion of culture negative infective endocarditis (Egger's test: t = -1.43, p = 0.1673)

For peer review only

PRISMA 2020 Checklist

Section and Topic	Item #	Checklist item	Location where item is reported
**TITLE**			
Title	1	Identify the report as a systematic review.	Page 1
**ABSTRACT**			
Abstract	2	See the PRISMA 2020 for Abstracts checklist.	Page 2-3
**INTRODUCTION**			
Rationale	3	Describe the rationale for the review in the context of existing knowledge.	Page 4-5
Objectives	4	Provide an explicit statement of the objective(s) or question(s) the review addresses.	Page 5
**METHODS**			
Eligibility criteria	5	Specify the inclusion and exclusion criteria for the review and how studies were grouped for the syntheses.	Page 6
Information sources	6	Specify all databases, registers, websites, organisations, reference lists and other sources searched or consulted to identify studies. Specify the date when each source was last searched or consulted.	Page 6-7
Search strategy	7	Present the full search strategies for all databases, registers and websites, including any filters and limits used.	Page 6-7
Selection process	8	Specify the methods used to decide whether a study met the inclusion criteria of the review, including how many reviewers screened each record and each report retrieved, whether they worked independently, and if applicable, details of automation tools used in the process.	Supplementary
Data collection process	9	Specify the methods used to collect data from reports, including how many reviewers collected data from each report, whether they worked independently, any processes for obtaining or confirming data from study investigators, and if applicable, details of automation tools used in the process.	Page 7
Data items	10a	List and define all outcomes for which data were sought. Specify whether all results that were compatible with each outcome domain in each study were sought (e.g. for all measures, time points, analyses), and if not, the methods used to decide which results to collect.	Page 7-8
	10b	List and define all other variables for which data were sought (e.g. participant and intervention characteristics, funding sources). Describe any assumptions made about any missing or unclear information.	Page 7-8
Study risk of bias assessment	11	Specify the methods used to assess risk of bias in the included studies, including details of the tool(s) used, how many reviewers assessed each study and whether they worked independently, and if applicable, details of automation tools used in the process.	Supplementary
Effect measures	12	Specify for each outcome the effect measure(s) (e.g. risk ratio, mean difference) used in the synthesis or presentation of results.	Page 7-8
Synthesis methods	13a	Describe the processes used to decide which studies were eligible for each synthesis (e.g. tabulating the study intervention characteristics and comparing against the planned groups for each synthesis (item #5)).	Page 6-8
	13b	Describe any methods required to prepare the data for presentation or synthesis, such as handling of missing summary statistics, or data conversions.	Page 6-8
	13c	Describe any methods used to tabulate or visually display results of individual studies and syntheses.	Page 6-8
	13d	Describe any methods used to synthesize results and provide a rationale for the choice(s). If meta-analysis was performed, describe the model(s), method(s) to identify the presence and extent of statistical heterogeneity, and software package(s) used.	Page 6-8
	13e	Describe any methods used to explore possible causes of heterogeneity among study results (e.g. subgroup analysis, meta-regression).	Page 8
	13f	Describe any sensitivity analyses conducted to assess robustness of the synthesized results.	Page 8
Reporting bias assessment	14	Describe any methods used to assess risk of bias due to missing results in a synthesis (arising from reporting biases).	N/A
Certainty assessment	15	Describe any methods used to assess certainty (or confidence) in the body of evidence for an outcome.	N/A

For peer review only - http://bmjopen.bmj.com/site/about/guidelines.xhtml

PRISMA 2020 Checklist

Section and Topic	Item #	Checklist item	Location where item is reported
**RESULTS**			
Study selection	16a	Describe the results of the search and selection process, from the number of records identified in the search to the number of studies included in the review, ideally using a flow diagram.	Page 9
	16b	Cite studies that might appear to meet the inclusion criteria, but which were excluded, and explain why they were excluded.	N/A
Study characteristics	17	Cite each included study and present its characteristics.	Table 1,2
Risk of bias in studies	18	Present assessments of risk of bias for each included study.	Supplementary
Results of individual studies	19	For all outcomes, present, for each study: (a) summary statistics for each group (where appropriate) and (b) an effect estimate and its precision (e.g. confidence/credible interval), ideally using structured tables or plots.	Table 1,2,3
Results of syntheses	20a	For each synthesis, briefly summarise the characteristics and risk of bias among contributing studies.	Page 9-14
	20b	Present results of all statistical syntheses conducted. If meta-analysis was done, present for each the summary estimate and its precision (e.g. confidence/credible interval) and measures of statistical heterogeneity. If comparing groups, describe the direction of the effect.	Figure 1,2 +supplementary
	20c	Present results of all investigations of possible causes of heterogeneity among study results.	Figure 1,2 +supplementary
	20d	Present results of all sensitivity analyses conducted to assess the robustness of the synthesized results.	Page 8
Reporting biases	21	Present assessments of risk of bias due to missing results (arising from reporting biases) for each synthesis assessed.	N/A
Certainty of evidence	22	Present assessments of certainty (or confidence) in the body of evidence for each outcome assessed.	N/A
**DISCUSSION**			
Discussion	23a	Provide a general interpretation of the results in the context of other evidence.	Page 14-19
	23b	Discuss any limitations of the evidence included in the review.	Page 14, 18-19
	23c	Discuss any limitations of the review processes used.	Page 18-19
	23d	Discuss implications of the results for practice, policy, and future research.	Page 14-19
**OTHER INFORMATION**			
Registration and protocol	24a	Provide registration information for the review, including register name and registration number, or state that the review was not registered.	Page 5
	24b	Indicate where the review protocol can be accessed, or state that a protocol was not prepared.	Page 5
	24c	Describe and explain any amendments to information provided at registration or in the protocol.	N/A
Support	25	Describe sources of financial or non-financial support for the review, and the role of the funders or sponsors in the review.	Page 21
Competing interests	26	Declare any competing interests of review authors.	Page 21
Availability of data, code and other materials	27	Report which of the following are publicly available and where they can be found: template data collection forms; data extracted from included studies; data used for all analyses; analytic code; any other materials used in the review.	N/A

From: Page MJ, McKenzie JE, Bossuyt PM, Boutron I, Hoffmann TC, Mulrow CD, et al. The PRISMA 2020 statement: an updated guideline for reporting systematic reviews. BMJ 2021;372:n71. doi: 10.1136/bmj.n71

For peer review only - http://bmjopen.bmj.com/site/about/guidelines.xhtml

For more information, visit: http://www.prisma-statement.org/

For peer review only
